# Randomized Controlled Trial of Integrated Kidney Supportive Care in End-Stage Kidney Disease

**DOI:** 10.1016/j.ekir.2026.106696

**Published:** 2026-07-08

**Authors:** Bharathi Naik, Shankar Prasad Nagaraju, Anuja Damani, Arun Ghoshal, Pankaj Singhai, Naveen Salins, Ravindra Prabhu Attur, Swathi Nayak Ammunje, Ajith M. Nayak, Mohan V. Bhojaraja, Srinivas Vinayak Shenoy, Dharshan Rangaswamy, Indu Ramachandra Rao

**Affiliations:** 1 Department of Renal Replacement Therapy and Dialysis Technology, Manipal College of Health Professions, Manipal Academy of Higher Education, Manipal, India; 2 Department of Nephrology, Kasturba Medical College, Manipal Academy of Higher Education, Manipal, India; 3 Department of Palliative Medicine and Supportive Care, Kasturba Medical College, Manipal Academy of Higher Education, Manipal, India; 4 Department of Palliative Medicine, Sri Aurobindo Medical College and PG Institute, Sri Aurobindo University, Indore, Madhya Pradesh, India

**Keywords:** advance care planning, kidney failure, kidney supportive care, quality of life, noncommunicable diseases, symptom burden

## Abstract

**Introduction:**

Kidney failure is linked to a substantial symptom burden and diminished quality of life (QoL). The incorporation of kidney supportive care (KSC) and advance care planning (ACP) into routine nephrology practices could enhance patient-centered outcomes; however, data from developing countries are scarce. This study evaluated the effects of KSC versus standard care on QoL, symptom burden, and ACP engagement in kidney failure.

**Methods:**

This randomized controlled trial was conducted at a tertiary care hospital in India from June 2023 to June 2025. Adult patients with kidney failure were randomized using block randomization to receive either integrated KSC or conventional care. Outcomes, including QoL, symptom burden, and ACP engagement, were assessed at baseline and at 2, 4, and 6 months using the Edmonton Symptom Assessment System-revised (ESAS-r) Renal tool, EuroQol 5-Dimensions, 5-Levels (EQ-5D-5L), and an ACP questionnaire.

**Results:**

Patients in the intervention group showed significant improvements across multiple domains, with QoL scores increasing markedly (*P* < 0.01) and symptom burden decreasing significantly (*P* < 0.01) compared with baseline and the control group. Additionally, the willingness of patients to engage in ACP discussions and documentation increased substantially in the KSC group, which was accompanied by a significantly higher rate of Cardiopulmonary Resuscitation refusal and a reduced willingness for ventilator or intensive care unit (ICU) admission (*P* < 0.01).

**Conclusion:**

Integrated KSC improves QoL, reduces symptom burden, and enhances ACP engagement. These findings support integrating structured KSC into routine kidney care in developing settings.

## Graphical abstract

**Figure:**
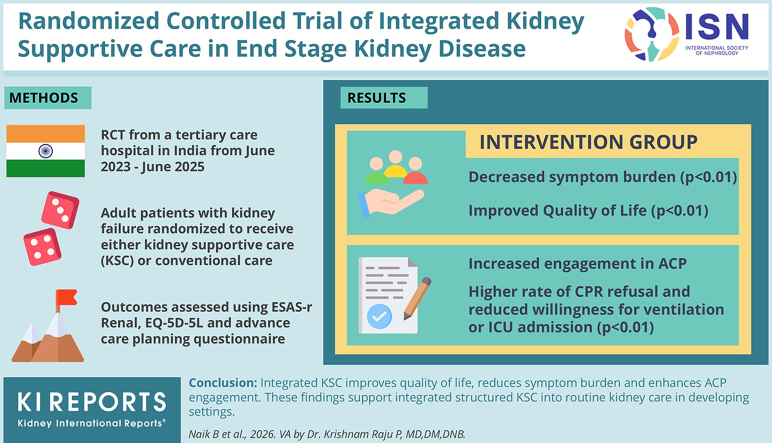

Kidney failure is a life-threatening condition.^1^ According to the Global Burden of Disease 2023 study, approximately 1.48 million people died from chronic kidney disease globally.^2^ Patients with kidney failure report a median number of symptoms ranging from 5.7 to 7.5^3^ Research indicates that the symptom burden in chronic kidney disease is not determined solely by disease severity, but also by the number of coexisting symptoms, both of which can significantly affect an individual’s QoL.^4^ Patients with kidney failure often experience a symptom burden comparable to, or even exceeding, that of individuals with advanced cancer.^5^

Dialysis can prolong a patient’s life, but it often compromises the quality of their remaining time, primarily because of a high symptom burden encompassing both physical and psychological aspects, which is particularly pronounced among individuals with low socioeconomic status.^6^, ^7^, ^8^, ^9^ Therefore, incorporating supportive care and ACP in patients with kidney failure has been shown to significantly improve QoL and patient care. A randomized controlled trial conducted in cardiology patients demonstrated its effectiveness, and these findings may also be applicable to individuals with other chronic illnesses, including kidney failure.^10^^,^^11^

KSC is an approach to care that aims to improve the QoL for people for whom kidney disease, either directly or indirectly, substantially impacts their well-being, treatment options, or access to care.^12^ KSC involves applying palliative care principles and practices to patients with kidney disease. The primary aim is to alleviate suffering by addressing symptoms, fostering empathetic communication, and providing support for individuals dealing with psychosocial distress. Ideally, KSC is delivered through a collaborative effort involving nephrologists and palliative care professionals, typically using an interdisciplinary team consisting of nurses, social workers, dietitians and, in some settings, chaplains.^13^^,^^14^

ACP enables individuals to define goals and preferences for future medical treatment and care, to discuss these goals and preferences with family and health-care providers, and to record and review these preferences if appropriate.^15^ It helps improve patients’ QoL, reduces hospital admissions and unnecessary treatments, enhances patient and family satisfaction with care, and improves end-of-life outcomes by reducing the financial burden associated with unwanted aggressive treatments near death.^16^^,^^17^ However, several barriers to ACP include perceptions of being healthy, limited awareness, emotional distress, and family-dominated decision-making. Additional barriers such as inadequate training, lack of clinical time, and cultural factors influencing communication patterns further hinder ACP implementation.^16^^,^^18^^,^^19^

In India, KSC remains an evolving and underdeveloped component of KSC, with variable awareness and acceptance among patients and health care providers. Our previous work highlighted a substantial lack of awareness and utilization of KSC among patients with kidney failure, although many patients expressed willingness to engage in such care when needed. Patients and caregivers prioritized detailed information about illness, treatment options, disease progression, and complications. A collaborative approach to decision-making was preferred by most, involving patients, their families, and health care providers. However, only 9% were willing to formally document ACP.^19^ Among nephrology health care providers, although the importance of ACP is widely recognized, key barriers include limited training and cultural challenges. Providers emphasized the need for structured protocols, educational initiatives, multidisciplinary approaches, and institutional support to enable effective implementation of ACP.^20^

The study hypothesis is that the combination of nephrology and supportive care may reduce the total symptom burden and enhance QoL. Despite the significant burden of kidney failure in developing countries, the potential benefits of focused integrated KSC remain largely unexplored.^16^ Family discussion and ACP may help in achieving a better QoL, care, and family satisfaction.^21^

The Western literature indicates that randomized controlled trials have demonstrated that the integration of palliative care in chronic illnesses effectively reduces symptom burden and improves QoL.^11^^,^^22^ Additionally, 1 pilot randomized control trial reported that the integration of palliative care and nephrology care improved symptom burden, ACP documentation, and the overall disease experience.^23^

The dearth of literature on the impact of focused QoL interventions, such as KSC, on patients of kidney failure in developing countries necessitates further exploration. To the best of our knowledge, our study is the first of its kind in India to assess the effect of integrated KSC on QoL, symptom burden, and ACP. Therefore, this study aimed to evaluate the effect of KSC, compared with conventional care, on QoL, symptom burden, and patients’ willingness to engage in ACP discussions and documentation among individuals with kidney failure.

## Methods

### Trial Design and Participants

This study was a randomized controlled trial in which participants were allocated to the experimental and control groups using block randomization to ensure balanced group distribution, and it was conducted in the Hemodialysis Unit, Nephrology Outpatient Department, and KSC Clinic at a tertiary care hospital in India. Ethical approval was obtained from the institutional ethics committee (IEC-214/2021), and the study was registered with the Clinical Trials Registry of India (CTRI/2021/06/034179; Supplementary 1). The study was carried out between June 2023 and June 2025. KSC services were provided through a dedicated clinic integrated within the nephrology department and colocated with the dialysis unit, delivered by a multidisciplinary team consisting of palliative care physicians, nephrologists, a trained KSC coordinator, and a social worker with experience in nephrology and palliative care. The palliative care physicians had formal specialist training, whereas the nephrologists and KSC coordinator underwent in-house training, including participation in a KSC workshop and the Impact Nephrocare program, along with supervised clinical training. Before inclusion, participants were assessed by a trained research coordinator using the Australia-modified Karnofsky Performance Status scale for functional status.^24^ Symptoms were assessed using the ESAS-r Renal tool,^25^^,^^26^ and QoL was measured using the EQ-5D-5L questionnaire.^27^ Patients who fulfilled the following criteria were eligible: adult patients of kidney failure aged > 18 years with an estimated glomerular filtration rate < 15 ml/min per 1.73 m^2^ and a Karnofsky Performance Status score < 70, indicating greater functional impairment, and presenting with mild to moderate symptoms (≥ 4 on any ESAS-r Renal symptom) and impaired QoL (≥ 3 on any EQ-5D-5L dimension) were included. Patients who were unable to complete the questionnaires because of known psychological or cognitive disorders, as identified from medical records, were excluded.

### Study Description

Patients of kidney failure who visited the outpatient, inpatient, or dialysis unit were screened for the study. During the initial screening visit (visit 0), the investigator verified that patient enrollment met the following requirements:

• The patient met the inclusion criteria. The patient signed and dated the informed consent forms.
• The patients underwent all necessary baseline assessments, which were recorded in their medical records by the research coordinator.
• The patient completed the case record form (s) for eligibility and registration, which were signed and dated by the investigator. Additionally, the patients completed the baseline EQ-5D-5L and ESAS-r Renal questionnaires.

**Figure 1:**
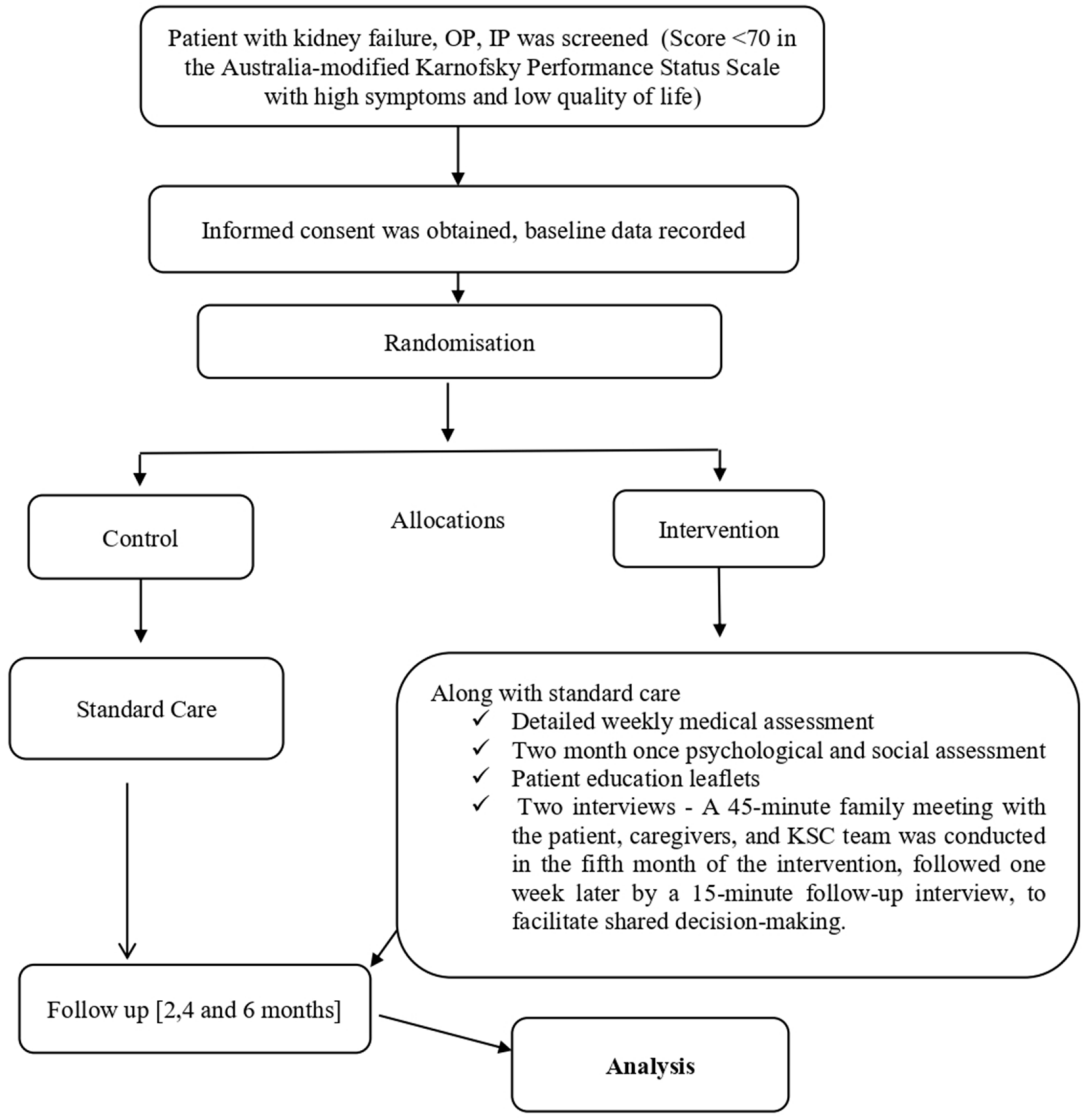
Flow diagram of the study process.

Validated translated versions of the questionnaires in Kannada or English languages were used for data collection according to patient preference^24^, ^25^, ^26^, ^27^, ^28^, ^29^, ^30^, ^31^, ^32^ (Supplementary 2,^3^,^4^,^5^). Immediately after randomization, patients were assigned to either the control or intervention group. Patients in the intervention group were referred to the KSU unit for further care. Both patient groups were evaluated at baseline (visit 0) and every 2, 4, and 6 months thereafter for 6 months (Figure 1). Each evaluation consisted of completing the ESAS-r Renal and EQ-5D-5L questionnaires and documenting the patient’s willingness to engage in ACP discussions and documentation through the ACP questionnaire.

### Intervention

The focused KSC intervention was delivered by a multidisciplinary team including nephrologists, palliative care physicians, a KSC coordinator, and medical social workers. Before the start of the intervention, eligible participants were assessed at baseline for QoL using the EQ-5D-5L and for symptom burden using the ESAS-r Renal tool. Patients then underwent weekly symptom burden assessments using the self-administered ESAS-r Renal tool, with assistance from a KSC coordinator if required, along with medical assessments by a palliative care physician, including review of comorbidities and ongoing treatments. Those with moderate to severe symptoms received continued weekly follow-up until adequate symptom control was achieved, guided by an evidence-based symptom management algorithm and appropriate medical treatment. The nephrologist supervised kidney-related management, whereas the palliative care physician led symptom management. The KSC coordinator conducted weekly assessments, monitored patients, and coordinated care.

Psychological and social assessments were conducted every 2 months by a medical social worker using validated tools (Patient Health Questionnaire-9, Multidimensional Scale of Perceived Social Support, and Generalized Anxiety Disorder-7), along with psychosocial support and group sessions. Nonpharmacological support included a symptom management algorithm that was developed based on existing evidence from published palliative care and nephrology guidelines, along with recommendations from previous studies and expert consensus (Supplementary 6).^33^ It was adapted to our clinical setting and translated into Kannada to ensure feasibility and patient understanding. These educational leaflets were distributed by the KSC coordinator. Follow-up assessments were conducted at 2 and 4 months, during which participants completed the ESAS-r, Renal, EQ-5D-5L, and ACP questionnaires by the research coordinator.

In the fifth month, the entire KSC team met with the patient and caregivers, and communication among KSC team members was structured through joint discussions. Two semistructured interviews were conducted using an interview guide to discuss goals of care and ACP, including preferences regarding place of care, treatment interventions, resuscitation, mechanical ventilation, ICU admission, and identification of a surrogate decision-maker (Supplementary 7). The first session lasted approximately 45 minutes and was followed by a shorter 15-minute follow-up meeting conducted within 1 to 2 weeks. After 6 months of intervention, QoL, symptom burden, and patient’s willingness to engage in ACP discussions and documentation were reassessed using the same questionnaires.

**Figure 2:**
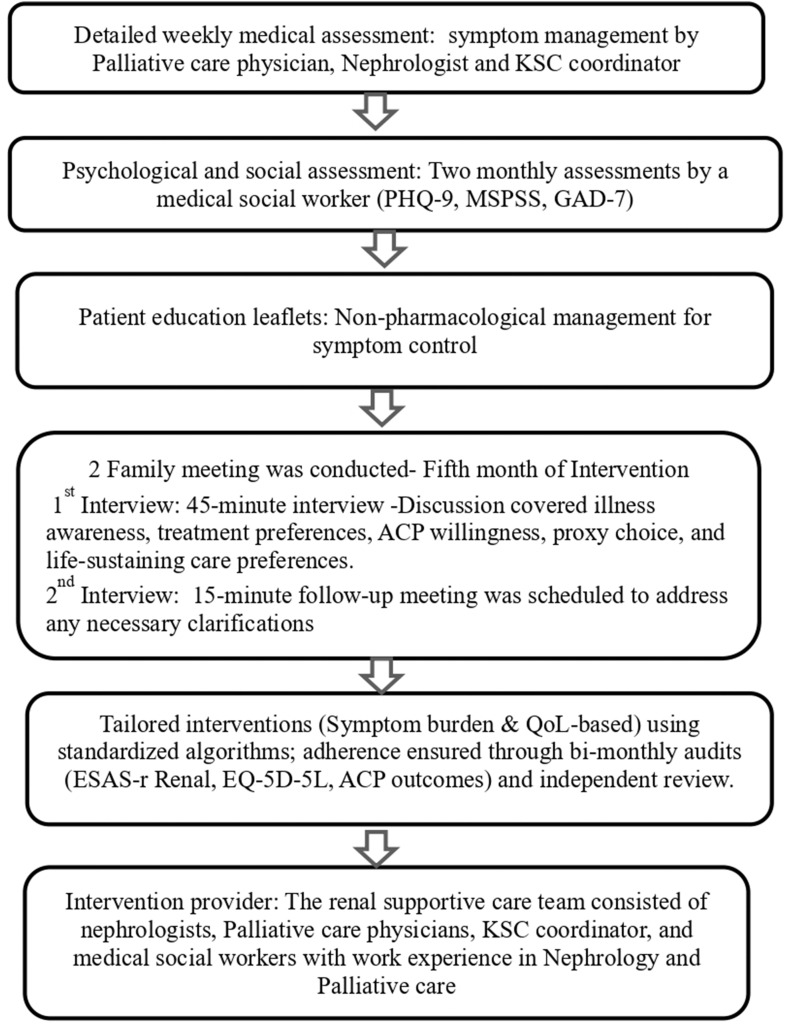
Flow diagram of the focused kidney supportive care intervention process.

Interventions were delivered through face-to-face consultations, printed materials, and telephone follow-up, primarily in dialysis units and supportive care clinics, and were tailored based on ESAS-r scores and QoL domains using standardized symptom management algorithms and medical interventions. Adherence to the intervention was monitored through bimonthly audits conducted by independent team members (Figure 2).

### Control Arm

Patients in the control group received standard kidney care, with dialysis schedules determined by the treating nephrologists. Symptom management and supportive care were provided by the primary nephrologists. Referral to a palliative care physician was permitted if required; however, no participants opted for referral during the study period. QoL and symptom burden in the control group were assessed at baseline during inclusion and subsequently at 2, 4, and 6 months, similar to the intervention group, using the EQ-5D-5L and ESAS-r Renal tools. Patient’s willingness to engage in ACP discussions and documentation was also assessed using an ACP questionnaire.

### Follow-Up Assessments

Patients in both groups had their QoL, symptom burden, and understanding of their condition, treatment preferences, and willingness to engage in ACP assessed at 2, 4, and 6 months using the self-administered EQ-5D-5L, ESAS-r Renal, and an ACP questionnaire in their preferred language to ensure feasibility and understanding. The assessments were facilitated by a research coordinator who was blinded to group allocation. For patients unable to return to the hospital, efforts were made to contact them by telephone to complete the questionnaires.

### Outcomes

The primary outcome was QoL, assessed using the EQ-5D-5L questionnaire at 6 months. The secondary outcomes included symptom burden, measured using the ESAS-r Renal scale, and engagement in ACP, assessed using an ACP questionnaire, including patients’ willingness to engage in ACP discussions and documentation.

### Determination of Sample Size

The sample size was calculated based on the primary outcome of change in QoL measured using the EQ-5D-5L at 6 months postrandomization. An effect size assumption of Cohen’s *d* = 0.5, representing a generic moderate effect, was used, with a 2-sided alpha of 0.05 and 80% power, yielding 63 participants per group. After adjusting for a 30% anticipated attrition rate, the final sample size was increased to 90 participants per group, providing a conservative estimate.

### Randomization

Participants were randomized using a computer-generated block randomization sequence (block size of 10) to ensure balanced group allocation. The allocation sequence was prepared and securely stored in an Excel sheet by an independent statistician with no affiliation to the clinical team, ensuring allocation concealment. Participants were enrolled by a KSC coordinator, and interventions were assigned by the KSC coordinator and KSC team members, based on the group allocation provided by the independent statistician according to the concealed sequence. A total of 90 patients were randomized to each group, with no stratification applied.

The study was unblinded, as both participants and investigators were aware of group allocation. Blinding was not feasible because of the nature of the intervention (interviews); however, outcome assessments were conducted by a research coordinator blinded to group allocation. Data analysis was performed by a statistician who was also blinded to allocation, with group codes masked to minimize bias.

### Data Entry and Statistical Analysis

Data were entered in Microsoft Excel 2010 and analyzed using Jamovi (version 2.3.28). Descriptive statistics included frequencies and percentages for categorical variables and means with SDs for continuous variables. For ESAS-r Renal and EQ-5D-5L, the minimally clinically important difference was defined as a ≥ 1-level change from baseline to 6 months^34^^,^^35^ and between-group comparisons were expressed as relative risk (RR) with 95% confidence intervals (CI). The primary analysis followed a per-protocol approach, including participants with complete data, with no imputation for missing values; longitudinal changes were assessed using the Friedman test with *post hoc* Wilcoxon signed-rank tests, without adjustment for multiple comparisons. A sensitivity analysis using the intention-to-treat population applied last observation carried forward^36^ with significance set at α = 0.05. Binomial logistic regression identified factors associated with symptom burden, QoL, and patients’ willingness for ACP, including age, gender, education, financial status, family support, and dialysis vintage, reported as coefficients (B), adjusted odds ratios (ORs), 95% CIs, and *P*-values. ACP interview data collected at 2 time points were analyzed descriptively using frequencies, and themes were derived by grouping similar responses rather than formal qualitative analysis.

## Results

### Recruitment and Baseline Assessment

**Figure 3:**
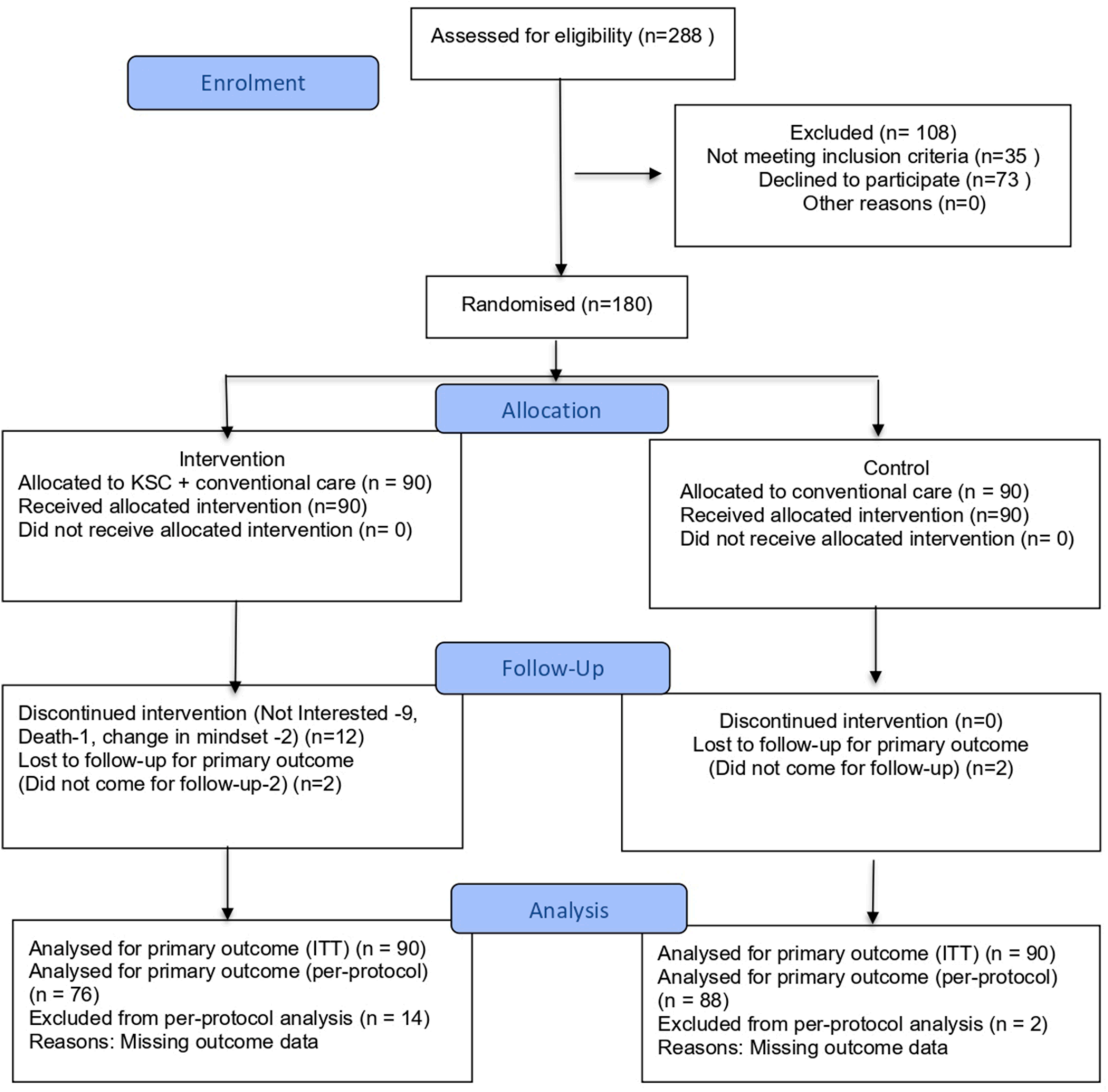
CONSORT flow diagram of the study.

Among the 288 patients of kidney failure screened, 180 were enrolled and randomized (90 per arm). Participants were recruited over 2 years, and each patient was followed for up to 6 months. The trial was completed as planned, with no early stopping. The intervention was delivered by the KSC team, including the KSC coordinator and multidisciplinary members, as per the study protocol, whereas standard care was provided to the control group. Overall, the intervention was delivered as intended, and adherence was monitored throughout the study period. Participants in both groups continued to receive routine clinical care as per institutional protocols during the trial. Fourteen participants (15.5%) in the intervention group and 2 (2.2%) in the control group were lost to follow-up, yielding an overall attrition rate of 8.8%. Missing data were primarily because of lack of interest (*n* = 9), loss to follow-up (*n* = 4), death (*n* = 1), and change in mindset (*n* = 2), as shown in Figure 3; although dropout was higher in the intervention group, an attrition rate of up to 30% had been anticipated. The intention-to-treat analysis included all randomized patients (*n* = 180) with last observation carried forward applied for missing data, whereas the per-protocol population comprised 76 intervention and 88 control participants. Primary outcomes were consistent across intention-to-treat and per-protocol analyses (Supplementary 8 Tables S1–S4). The baseline characteristics were well balanced between groups. As shown in Table 1, participants in both groups were comparable, with a median age of 58 years in the intervention group and 53 years in the control group. The relatively younger age distribution reflects the typical patient population at our center and was not because of selection bias. Similar KPS scores (50), and median dialysis vintage of 5 (3–8) months in the intervention group and 3 (1.54–6.46) months in the control group. The majority were male, had a high prevalence of hypertension and diabetes, belonged mainly to middle- and low-income groups, and demonstrated strong family support, indicating a representative patients of kidney failure.

**Table 1:** Demographic characteristics of the intervention and control group

Demographic variables	Intervention (*N* = 90)	Control (*N* = 90)
Age, median (IQR)	58 (50.5–65.5)	53 (43.6–62.4)
KPS score, median (IQR)	50 (40–60)	50 (40–60)
Dialysis vintage, median (IQR)	5 (3–8)	3 (1.54–6.46)
Gender		
Male, *n* (%)	63 (70.0%)	66 (73.3%)
Female	27 (30.0%)	24 (26.7%)
Comorbidity		
Diabetes Mellitus	38 (39.18%)	31 (31.96%)
Hypertension	75 (77.32%)	80 (82.47%)
Ischemic heart disease	21 (21.65%)	15 (15.46%)
Asthma	5 (3.3%)	2 (2.2%)
Chronic obstructive pulmonary disease	0 (0%)	1 (1.03%)
Chronic liver disease	3 (3.09%)	1 (1.03%)
Stroke	5 (5.15%)	2 (2.06%)
Epilepsy	2 (2.06%)	0 (0%)
Allergy	0 (0%)	3 (3.09%)
Obesity	0 (0%)	0 (0%)
Human immunodeficiency virus infection	0 (0%)	0 (0%)
Tuberculosis	2 (2.06%)	0 (0%)
Cancer	0 (0%)	0 (0%)
CKD etiology		
Diabetic	38 (39.58%)	30 (31.25%)
Hypertension	10 (10.42%)	23 (23.96%)
Chronic Glomerulonephritis (CGN)	24 (25.00%)	18 (18.75%)
Chronic Interstitial Nephritis (CITN)	7 (7.29%)	11 (11.46%)
ADPKD	2 (2.08%)	2 (2.08%)
Others: Lupus Nephritis, Hypothyroidism, etc.	9 (9.38%)	6 (6.25%)
Financial status		
Affluent	14 (16%)	5 (5%)
Middle-income	39 (43%)	44 (49%)
Below Poverty Line (BPL)	37 (41%)	41 (46%)
Educational qualification		
Graduate or above	21 (28%)	28 (32%)
Secondary education (high school)	33 (43%)	26 (30%)
Primary education	4 (4%)	4 (4%)
No formal education	18 (25%)	30 (34%)
Patient category		
Patients undergoing hemodialysis	81 (90%)	73 (82%)
Patients not on dialysis	9 (10%)	17 (18%)
Family support		
Good family support	86 (96%)	89 (99%)
Poor family support	4 (4%)	1 (1%)

ADPKD, autosomal dominant polycystic kidney disease; CKD, chronic kidney disease; IQR, interquartile range.

### Effect of Integrated KSC on Symptom Burden

Using the ESAS-r Renal at baseline, and at 2, 4, and 6 months, the intervention group showed significant reductions in overall symptom burden over time (*P* < 0.01), whereas the control group showed minimal change and increased nausea (*P* < 0.01; Table 2).

**Table 2:** Changes in symptom burden among the intervention and control groups in different timepoints

	Intervention					Control				
	Baseline (*N* = 90)	2 mo (*N* = 81)	4th mo (*N* = 79)	6th mo (*N* = 76)	*P*-value	Baseline (*N* = 90)	2 mo (*N* = 89)	4th mo (*N* = 88)	6th mo (*N* = 88)	*P* value
ESAS-r symptoms score distribution	Mean ± SD	Mean ± SD	Mean ± SD	Mean ± SD		Mean ±SD	Mean ± SD	Mean ± SD	Mean ± SD	
Pain	3.03 ± 2.87	1.92 ± 2.05	1.59± 1.99	1.33 + 1.84	< 0.01^a^	2.49 + 2.83	2.2 + 2.57	2.44 + 2.5	2.8 + 2.44	0.15
Tiredness (Fatigue)	5.0 ±2.50	4.79 ± 1.55	3.88 ± 1.88	3.25 + 1.49	< 0.01^a^	4.68 + 2.55	4.96 + 2.04	4.53 + 1.84	4.86 + 1.99	0.99
Drowsiness	1.13 ± 1.9	0.68 ±1.22	0.56 ± 1.43	0.36 + 0.84	< 0.01^a^	1.25 + 2.03	1.97 + 2.44	1.66 + 2.0	1.63 + 2.0	0.08
Nausea	1.19 ±1.75	1.03± 1.59	0.59 ± 1.10	0.59 +0.89	< 0.01^a^	1.17 + 1.77	1.7 + 1.82	1.88 + 1.85	2 + 2.12	<0.01^a^
Lack of appetite	2.06 ± 2.45	1.38 ± 1.80	0.83 ± 1.32	0.95 + 1.28	< 0.01^a^	1.55 + 2.03	1.83 + 1.99	1.66 + 1.79	1.94 + 1.95	0.28
Shortness of breath	1.37 ± 2.10	0.97 ± 1.37	0.84 ± 1.23	0.43 + 0.99	0.05^a^	1.34 + 1.89	1.59 + 2.16	1.53 + 1.77	1.57 + 1.95	0.55
Depression	1.34 ± 1.8	1.64 ± 1.75	1.09 ± 1.83	0.89 + 1.35	0.03^a^	1.66 + 2.3	1.88 + 2.39	2 + 2.34	2 + 2.18	0.07
Anxiety	1.33 ± 1.94	1.36 ± 1.68	1.0 ± 1.47	0.6 + 1.1	< 0.01^a^	1.29 + 2	1.26 + 1.79	1.66 + 1.96	1.77 + 1.92	0.06
Poor Wellbeing	3.73 ± 2.28	3.83 ± 1.69	3.23 ± 1.96	2.73 + 1.29	< 0.01^a^	4.27 + 1.65	4.47 + 1.83	4.26 + 1.95	4.35 + 1.62	0.30
Itching	1.3 ± 2.03	0.92 ± 1.57	0.69 ± 1.47	0.65 + 0.98	0.03^a^	1.34 + 2.12	1.47 + 2.03	1.98 + 2.15	2.02 + 2.38	0.06
Sleeplessness	3.08 ± 2.83	1.99 ± 1.96	1.88 ± 1.98	1.22 + 1.38	< 0.01^a^	1.92 + 2.52	2.62 + 2.57	2.45 + 2.26	2.69 + 2.27	0.06
Others	2.12 ± 2.79	0.86 ± 1.39	0.67 ± 1.22	0.44 + 1.09	< 0.01^a^	1.32 + 2.03	1.92 + 2.26	1.29 + 1.98	1.36 + 2.23	0.15
Overall	2.21 ± 1.14	1.85 ± 0.87	1.44 ± 0.89	1.23 ± 0.79	< 0.01^a^	1.97 ± 1.11	2.24 ± 1.11	2.27 ± 1.06	2.41 ± 1.13	0.07

ESAS-r, Edmonton Symptom Assessment System-revised.

a *P* < 0.05 was considered statistically significant.

Patients in the intervention arm demonstrated markedly greater improvements across most symptoms. Improvements were strongest for fatigue (RR = 1.47), appetite loss (RR = 1.55), and poor well-being (RR = 1.84). Worsening was lower in the intervention group for pain (18% vs. 44%), nausea (19% vs. 48%), anxiety (11% vs. 40%), shortness of breath (15% vs. 35%), drowsiness (11% vs. 41%), and sleeplessness (18% vs. 50%). Overall, 75% improved in the intervention group versus 35% in the control group, whereas 24% worsened versus 61% in the control group, indicating a strong clinical benefit (Table 3).

**Table 3:** ESAS-r Renal: Clinically meaningful symptom change— intervention versus control in patients of kidney failure

ESAS-r symptoms score distribution	Intervention *N* = 76			Control *N* = 88			Symptoms improved vs. no changes risk ratio 95% CI	Symptom worsen vs. no changes risk ratio, 95% CI
	MCID achieved with Improved symptoms	MCID with worsening symptoms	No symptom changes	MCID achieved with Improved symptoms	MCID with worsening symptoms	No symptom changes		
Pain	40 (53%)	14 (18%)	22 (29%)	28 (32%)	39 (44%)	21 (24%)	1.13 (0.84–1.54)	0.59 (0.38–0.94)
Tiredness (Fatigue)	55 (72%)	17 (23%)	4 (5%)	33 (37%)	36 (41%)	19 (22%)	1.47 (1.19–1.83)	1.23 (0.93–1.65)
Drowsiness	25 (33%)	8 (11%)	43 (56%)	20 (23%)	36 (41%)	32 (36%)	0.98 (0.62–1.56)	0.30 (0.15–0.59)
Nausea	26 (34%)	14 (19%)	36 (47%)	15 (17%)	42 (48%)	31 (35%)	1.29 (0.78–2.14)	0.49 (0.29–0.79)
Lack of appetite	35 (46%)	17 (22%)	24 (32%)	20 (23%)	36 (41%)	32 (36%)	1.55 (1.03–2.3)	0.79 (0.52–1.20)
Shortness of breath	24 (32%)	12 (15%)	40 (53%)	24 (27%)	31 (35%)	33 (38%)	0.9 (0.58–1.39)	0.48 (0.28–0.84)
Depression	25 (33%)	21 (28%)	30 (39%)	21 (24%)	39 (44%)	28 (32%)	1.06 (0.69–1.64)	0.70 (0.49–1.05)
Anxiety	23 (30%)	8 (11%)	45 (59%)	17 (19%)	35 (40%)	36 (41%)	1.05 (0.64–1.77)	0.31 (0.15–0.60)
Poor wellbeing	47 (62%)	19 (25%)	10 (13%)	26 (30%)	30 (34%)	32 (36%)	1.84 (1.35–2.5)	1.36 (0.94–1.96)
Itching	26 (34%)	16 (21%)	34 (45%)	16 (18%)	34 (39%)	38 (43%)	1.47 (0.88–2.42)	0.68 (0.43–1.08)
Sleeplessness	41 (54%)	14 (18%)	21 (28%)	22 (25%)	44 (50%)	22 (25%)	1.33 (0.94–1.87)	0.6 (0.39–0.94)
Others	28 (37%)	9 (12%)	39 (51%)	26 (30%)	20 (22%)	42 (48%)	1.09 (0.73–1.66)	0.59 (0.29–1.16)
Overall	57 (75%)	18 (24%)	1 (1%)	31 (35%)	54 (61%)	3 (3%)	1.08 (0.97–1.20)	1 (0.89–1.14)

ESAS-r, Edmonton Symptom Assessment System-revised; MCID, minimally clinically important difference.

The control group was used as the reference for calculating risk ratios.

### Effect on QoL

The QoL (EQ-5D-5L)score improved significantly across all domains in the intervention group , whereas no improvements were observed in the control group. The intervention substantially increased the proportion of individuals who achieved minimally clinically important difference in mobility, self-care, usual activities, pain and/or discomfort, anxiety and/or depression, overall QoL (78% vs. 31%), and overall health (70% vs. 25%) (Table 4).

**Table 4:** QoL among the intervention and control groups at different timepoints

	Intervention					Control				
	Baseline (*N* = 90)	2 mo (*N* = 81)	4 mo (*N* = 79)	6 mo (*N* = 76)	*P*-value	Baseline (*N* = 90)	2 mo (*N* = 89)	4 mo (*N* = 88)	6 mo (*N* = 88)	*P* value
EQ-5D-5L score distribution	Mean ±SD	Mean ±SD	Mean ±SD	Mean ±SD		Mean ±SD	Mean ±SD	Mean ±SD	Mean ±SD	
Mobility	1.85 ± 1.23	1.47 ± 1.16	1.34 ± 1.23	0.99 ± 1.06	< 0.01^a^	1.54 ± 1.17	1.49 ± 1.05	1.46 ± 0.94	1.57 ± 1.03	0.99
Self-care	0.94 ± 1.12	0.89 ± 1.12	0.68 ± 1.01	0.58 ± 0.86	< 0.01^a^	0.94 ± 1	1.13 ± 1.07	1.03 ± 0.99	1.29 ± 0.93	0.07
Usual activities	1.65 ± 1.35	1.29 ± 1.18	0.92 ± 1.03	0.54 ± 0.74	< 0.01^a^	1.34 ± 1.3	1.38 ± 0.96	1.39 ± 0.84	1.5 ± 0.89	0.29
Pain/ discomfort	1.48 ± 0.99	1.09 ± 0.85	0.88 ± 0.83	0.58 ± 0.74	< 0.01^a^	1.23 ± 0.97	1.42 ± 0.79	1.49 ± 0.74	1.44 ± 0.79	0.10
Anxiety/depression	1.23 ± 1.07	0.99 ± 0.96	0.94 ± 0.93	0.4 ± 0.55	< 0.01^a^	0.978 ± 1.05	1.02 ± 1.02	1 ± 0.95	1.2 ± 1.07	0.134
Overall QoL	1.03 ± 0.97	0.744 ± 0.855	0.716 ± 0.840	0.338 ± 0.553	< 0.01^a^	1.2 ± 0.839	1.27 ± 0.744	1.31 ± 0.635	1.40 ± 0.750	0.06
Health	57.39 ± 15.32	59.45 ± 11.2	62.84 ± 12.33	69.5 ± 11.75	< 0.01^a^	56.73 ± 14.96	51.13 ± 13.96	53.17 ± 14.0	52.9 ± 11.9	0.06

EQ_5D-5L, EuroQol 5-Dimensions 5-Levels; QoL, quality of life.

a *P <* 0.05 was considered statistically significant.

Significant adjusted risk ratios favored the intervention for improvements in mobility (RR = 1.53), anxiety/depression (RR = 1.74), and overall health (RR = 1.62), with reduced risk of worsening in pain and/or discomfort, usual activities, and self-care domains (Table 5).

**Table 5:** EQ-5D-5L: Clinically meaningful QoL change— Intervention versus Control in patients with kidney failure

EQ-5D-5L score distribution	Intervention N=76			Control N=88			Symptoms improved vs. no changes risk ratio 95% CI	Symptom worsen vs. no changes risk ratio, 95% CI
	MCID achieved with Improved symptoms	MCID with worsening symptoms	No symptom changes	MCID achieved with improved symptoms	MCID with worsening symptoms	No symptom changes		
Mobility	45 (59%)	8 (11%)	23 (30%)	26 (30%)	28 (32%)	34 (38%)	1.53 (1.09–2.14)	0.57 (0.29–1.10)
Self-care	30 (39%)	11 (14%)	35 (47%)	21 (24%)	39 (44%)	28 (32%)	1.07 (0.72–1.64)	0.42 (0.23–0.72)
Usual activities	46 (61%)	7 (9%)	23 (30%)	26 (30%)	39 (44%)	23 (26%)	1.26 (0.91–1.72)	0.38 (019–0.73)
Pain/ discomfort	46 (60%)	5 (7%)	25 (33%)	23 (26%)	38 (43%)	27 (31%)	1.40 (0.99–1.99)	0.29 (0.13–0.66)
Anxiety/depression	43 (57%)	9 (11%)	24 (32%)	20 (22%)	34 (39%)	34 (39%)	1.74 (1.18–2.56)	0.55 (0.29–0.99)
Overall QoL	59 (78%)	9 (11%)	8 (11%)	27 (31%)	51 (58%)	10 (11%)	1.20 (0.97–1.49)	0.64 (0.39–1.00)
Health	53 (70%)	15 (19%)	8 (11%)	22 (25%)	47 (53%)	19 (22%)	1.62 (1.2–2.19)	0.91 (0.65–1.3)

EQ-5D-5L, EuroQol 5-Dimensions 5-Levels; MCID, minimally clinically important difference.

The control group was used as the reference for calculating risk ratios.

### Effect on ACP Preferences

#### Illness-Information Preferences

The intervention group showed significant improvement in preferences for information on illness severity, treatment options, future symptoms, and survival estimates at all follow-up points (*P* < 0.05; Table 6). The control group showed no changes.

**Table 6:** Changes in illness-information preferences of patients of kidney failure in the intervention and control groups at different timepoints

ACP- questionnaire illness-information preferences	Intervention				Control			
	2 mo (*N* = 81)	4 mo (*N* = 79)	6 mo (*N* = 76)	*P*-value	2 mo (*N* = 89)	4 month (*N* = 88)	6 month (*N* = 88)	*P*-value
A.1.1. Do you prefer to know about your illness?	67 (83%)	67 (84%)	72 (95%)	0.04^a^	75 (84%)	69 (78%)	72 (82%)	0.70
A.1.2. Do you prefer to know about the severity of your illness?	61 (75%)	59 (75%)	69 (91%)	< 0.01^a^	66 (74%)	62 (70%)	63 (72%)	0.37
A.1.3. Do you prefer to know about the future course of your illness?	65 (80%)	61 (77%)	66 (87%)	0.04^a^	62 (70%)	59 (67%)	60 (68%)	0.50
A.1.4. Do you prefer to know about your treatment options?	57 (70%)	56 (71%)	68 (89%)	< 0.01^a^	66 (74%)	60 (68%)	66 (75%)	0.30
A.1.5. Do you prefer to know the success/side-effects of your treatment options?	55 (68%)	59 (75%)	66 (87%)	< 0.01^a^	64 (72%)	56 (64%)	63 (72%)	0.10
A.1.6. Do you prefer to know about the future symptoms?	53 (65%)	50 (63%)	67 (88%)	< 0.01^a^	54 (61%)	49 (56%)	58 (66%)	0.11
A.1.7. Do you prefer to know about the future complications because of illness or because of its treatment?	50 (61%)	46 (58%)	68 (89%)	< 0.01^a^	53 (60%)	46 (52%)	58 (66%)	0.06
A.1.8. Do you prefer to know about your expected length of survival?	50 (61%)	46 (58%)	66 (87%)	< 0.01^a^	43 (48%)	40 (45%)	49 (56%)	0.08

a *P <* 0.05 was considered statistically significant.

#### ACP Decision-Making Preferences

The patients’ willingness to engage in ACP discussions and documentation increased significantly in the intervention group over 6 months (*P* < 0.01). By 6 months, 54 participants expressed readiness to complete an ACP document, whereas minimal change was observed in the control group (Table 7).

**Table 7:** Changes in ACP decision-making in patients of kidney failure for intervention and control across different time points

ACP- questionnaire decision making	Intervention				Control			
	2 mo *N* = 81	4 mo *N* = 79	6 mo *N* = 76	*P* value	2 mo *N* = 89	4 mo *N* = 88	6 mo *N* = 88	*P* value
A.2.1. Future health care preferences and options should be discussed and planned	26 (32%)	24 (30%)	56 (74%)	< 0.01^a^	25 (28%)	22 (25%)	14 (16%)	0.46
A.2.2. I prefer my future health care preferences to be documented as an advance care plan	20 (25%)	19 (24%)	54 (71%)	< 0.01^a^	16 (18%)	19 (21%)	17 (19%)	0.56
A.2.3. I prefer to be part of all decision-making discussions concerning my current and future health care options	42 (51%)	35 (45%)	55 (72%)	< 0.01^a^	52 (58%)	45 (51%)	50 (57%)	0.27
A.2.4. I would prefer to make all the health care decisions myself	72 (89%)	71 (90%)	62 (82%)	0.13	62 (70%)	61 (69%)	67 (76%)	0.13
A.2.5. I would prefer family to make all the health care decisions	67 (83%)	66 (84%)	65 (86%)	0.68	59 (66%)	57 (65%)	66 (75%)	0.06
A.2.6. I would prefer health care provider (doctors/dialysis team) to make all the health care decisions	74 (91%)	65 (82%)	60 (79%)	0.68	54 (61%)	51 (58%)	60 (68%)	0.08
A.2.7. I would prefer myself, my family, and health care providers (doctors/dialysis team) are involved in the decision-making process	46 (57%)	57 (72%)	68 (89%)	< 0.01^a^	52 (58%)	47 (53%)	55 (63%)	0.14
A.2.8. I like my preferences, decisions and wishes made about my future treatment and general care process is respected and implemented	44 (54%)	34 (43%)	62 (82%)	< 0.01^a^	51 (57%)	44 (50%)	51 (58%)	0.19

a *P* < 0.05 was considered statistically significant.

Participation in shared decision-making increased from 51% to 72% in the intervention group (*P* < 0.01), with many shifting toward patient–family–clinician joint decision-making. No adverse events related to the intervention occurred.

#### Demographic Associations

Regression analyses revealed no demographic predictors for symptom burden or QoL. Male gender was associated with greater patients’ willingness to engage in ACP discussions and documentation (OR: 0.31; *P* = 0.027; Table 8), though this may reflect higher male representation. No other predictors were significant.

**Table 8:** Demographic factors associated with overall symptom burden, overall QoL, and patients’ willingness for ACP documentation

Overall symptom burden (*R*^2^ = 0.35)						Overall QoL (*R*^2^ = 0.24)					Willingness for ACP documentation (*R*^2^ = 0.40)				
Predictors	B	SE	Odd ratio	95 CI (β adjusted)	*P*-value	B	SE	Odd ratio	95 CI (β adjusted)	*P*-value	B	SE	Odd ratio	95 CI (β adjusted)	*P*-value
Age	Age > 60 years- Reference														
Below 60 yr	-0.86	0.54	0.42	0.14–1.2	0.10	0.31	0.46	1.36	0.55–3.43	0.50	-0.53	0.53	0.58	0.20–1.68	0.32
Gender	Female reference														
Male	-0.97	0.51	0.37	0.13–1.0	0.058	0.47	0.45	1.60	0.66–4.0	0.30	-1.17	0.52	0.31	0.10–0.87	0.027∗
Education status	Graduate - Reference														
Secondary education (high school)	-0.535	0.58	0.58	0.18–1.8	0.361	0.54	0.52	1.70	0.61–4.8	0.30	0.23	0.59	1.26	0.4–3.74	0.71
Primary education	-0.79	1.5	0.45	0.02–7.33	0.57	0.87	1.07	2.39	0.2–21.5	0.41	3.77	1.40	43.6	2.86–66.5	0.07
No formal education	-0.07	0.62	0.93	0.28–3.16	0.90	-0.17	0.57	0.83	0.21–2.5	0.90	1.27	0.75	3.57	0.80–15.80	0.09
Financial status	Affluent – Reference														
Middle-income	1.32	0.97	3.7	0.56–25.4	0.17	-0.08	0.75	0.91	0.20–3.9	0.90	0.32	0.79	1.38	0.28–6.61	0.68
Below Poverty Line (BPL)	1.54	0.99	4.70	0.66–33.3	0.12	-0.49	0.79	0.61	0.12–2.8	0.53	0.20	0.87	1.22	0.21–6.87	0.81
Family support	Good family support -Reference														
Poor family support	1.03	1.60	2.80	0.12–64.7	0.519	-2.12	1.19	0.120	0.009–1.2	0.076	1.18	1.4	3.28	0.17–61.5	0.42
Dialysis vintage	Vintage above 5 yr -Reference														
Vintage 1–5 yr	0.625	0.65	1.86	0.52–6.6	0.33	-0.07	0.534	0.92	0.32–2.66	0.88	0.36	0.60	1.44	0.44–4.68	0.54
Vintage below 1 yr	-0.04	0.81	0.95	0.19–4.7	0.95	0.35	0.65	1.43	0.39–5.28	0.58	-0.07	0.78	0.92	0.19–4.34	0.92
No dialysis	1.16	0.81	3.20	0.65–15.7	0.15	0.99	0.67	2.69	0.73–10.4	1.41	0.12	0.79	1.12	0.23–5.38	0.88

*P* < 0.05 was considered statistically significant.

### ACP Interview Findings

In the intervention group, 76 participants received the intervention. Among these, 62 agreed to participate in the interviews and completed both rounds.

The findings across the 2 interview rounds are as follows:

• Willingness to discuss crises and death: > 60% were open to these discussions.
• Place of care: Preference for hospital care increased from 46 to 53 participants over time.
• ICU and/or ventilator support: 51 participants declined these interventions.
• Cardiopulmonary resuscitation: Acceptance decreased from 23 to 16; refusals increased from 25 to 29.
• Dialysis: 51 participants wished to continue dialysis even during severe illness.
• Comfort versus longevity: Preference for comfort increased from 40 to 53 participants.
• Surrogate decision-making: Family members or family-plus-doctors were preferred.
• ACP documentation: 57 participants agreed after interview 1 and 54 after interview 2.

These findings show the high feasibility and acceptance of ACP when supported by structured KSC discussions.

## Discussion

In this randomized controlled trial, we evaluated integrated KSC in patients with kidney failure, representing the first such trial conducted in India (based on our knowledge). These findings align with Western evidence supporting the need for holistic care models for kidney failure.^23^^,^^37^^,^^38^ KSC significantly reduced the overall symptom burden. All ESAS-r Renal symptoms improved in the intervention group compared with the control group, which is consistent with previous supportive-care studies reporting similar benefits.^11^^,^^23^^,^^39^ The relatively low baseline ESAS-r Renal scores may reflect variability across symptom domains within this population and are comparable to international hemodialysis populations, where moderate overall symptom burden has been reported using median global scores.^40^ The lower mean in our cohort may be explained by the inclusion of patients with at least 1 moderately severe symptom alongside several mild symptoms, which reduced the overall average. In addition, contextual factors such as cultural differences in symptom reporting, the provision of structured care, and the relatively younger age of patients, which is consistent with Indian studies reporting a median age of around 53 years^41^ may have contributed to these findings.

QoL improved across all domains in the intervention group, whereas earlier pilot trials reported improvements mainly in the physical components.^23^

Patients in the intervention group, unlike controls, showed increased preferences for illness-related information over 6 months. KSC structured communication may enhance resilience, reduce anxiety, and strengthen trust.^42^, ^43^, ^44^ Although Western studies have reported frequent discussions of life expectancy,^45^, ^46^, ^47^, ^48^ Indian patients traditionally avoid such conversations because of cultural taboos surrounding death.^49^ Our previous findings also revealed low interest,^19^ but the KSC intervention increased the patients’ willingness to discuss life expectancy, suggesting greater openness to ACP.

KSC increased the preference for future health care planning and documentation, which is consistent with earlier pilot trials that integrated nephrology and palliative care.^23^ Indian patients typically prefer physician-led decision-making with strong family involvement, differing from Western patterns.^50^ Consistent with systematic reviews, decision-making is influenced by patient, family, and health care provider factors,^51^ and our findings align with these observations.

Many intervention-group participants were ready to engage in ACP discussions, including those involving end-of-life topics, paralleling improved communication and documentation observed in other studies.^52^ Most preferred hospital-based care, unlike Western populations that favor home or hospice care,^53^ which is consistent with Indian data showing higher rates of hospital death.^54^ In our setting, dialysis expenditure was largely borne by patients, which may have influenced decisions regarding continuation and intensity of treatment, including preferences for ICU care and life-sustaining interventions, in the context of ACP discussions in India. Many declined ICU care, ventilation, and half of the participants declined cardiopulmonary resuscitation, indicating the influence of KSC on critical-care preferences. Previous studies note low do-not-resuscitate acceptance because of limited ACP awareness.^55^

Despite this, participants wished to continue dialysis even during life-threatening events, reflecting misconceptions about its curative potential.^56^^,^^57^ The KSC helps patients prioritize comfort and QoL through personalized discussions and shared decision-making.^37^^,^^58^ Many patients expressed a willingness to engage in ACP discussions, complete an ACP document, and preferred shared decision-making involving family members and clinicians.^16^^,^^42^ Ongoing ACP conversations remain essential.

In our multivariable analysis, socioeconomic variables such as financial status, education, and family support were not statistically significant predictors of study outcomes. However, these factors may still be relevant in shaping access to kidney care and health care decision-making in the Indian context.^19^^,^^59^^,^^60^ As this is the first known study evaluating an integrated KSC model in India, these findings provide important preliminary insights into the potential influence of socioeconomic and cultural factors.

The effectiveness of the intervention may be attributed to its multicomponent approach, including symptom management, structured communication, family involvement, and psychosocial support, which likely improved symptom control, understanding, decision-making, coping, and overall well-being. These components may have enhanced acceptability and impact within the study context. The intervention was feasible because of the presence of a dedicated KSC coordinator, who played a central role in monitoring and coordinating care. With such a structured approach, the KSC model can be effectively adapted and streamlined for routine clinical practice, particularly in tertiary care settings with trained personnel. In peripheral centers, implementation remains feasible but may need to be tailored based on available resources, and the intensity of the intervention can be adjusted according to local logistical constraints. Future research should include multicenter studies to improve generalizability and evaluate specific training programs, protocols, and context-appropriate approaches to inform implementation and scale-up.

### Strengths and Limitations

A major strength of this study is that it represents the first known randomized controlled trial in India to implement a KSC model within the routine management of patients with kidney failure. This demonstrates the feasibility and clinical value of integrating KSC into standard nephrology practice and highlights the crucial role of multidisciplinary care in addressing the complex needs of patients with kidney failure. Nevertheless, certain limitations should be acknowledged. Its single-center design may limit applicability to other populations. Additionally, the inclusion of patients with poorer functional status (Karnofsky score < 70) may have influenced the observed magnitude of effect and limits applicability to less symptomatic populations. ACP outcomes were measured as patients’ willingness rather than completion of legal documentation. The intervention was unblinded, as blinding was not feasible, which may have introduced bias. Missing data in the intention-to-treat analysis were handled using the last observation carried forward method, which may introduce bias due to its assumption of no change over time. Furthermore, reliance on per-protocol analysis may limit the generalizability of the findings. The repeated-measures approach may not fully account for within-subject variability over time, potentially weakening statistical associations. Additionally, exploratory analyses without adjustment for multiple comparisons may increase the risk of type I error and limit interpretation. minimally clinically important difference-based analysis may overlook clinically important changes between symptom states despite similar score differences. Finally, subgroup analysis between dialysis and conservative care patients was not performed, limiting assessment of differences in ACP perspectives. Each participant was followed for 6 months; although a longer follow-up would better assess sustainability, this was not feasible because of the intensive nature of the intervention.

## Conclusion

The KSC intervention provided reassurance to patients with kidney failure and improved care coordination for individuals with medical needs. In this randomized controlled trial, patients receiving KSC demonstrated significant reductions in symptom burden (*P* < 0.01) compared with standard care, with improvements observed across multiple symptom domains. QoL also improved significantly in the intervention group (*P* < 0.01). Additionally, patients in the intervention group showed a greater willingness to engage in ACP, including higher rates of CPR refusal and lower preference for intensive interventions (*P* < 0.01). These findings suggest that integrated KSC can improve symptom outcomes and support informed decision-making in patients with kidney failure. It also highlights the importance of regular discussions on ACP, which involves considering individuals’ preferences regarding the place of care, do-not-resuscitate orders, and advance care directives.

## Disclosure

All the authors declared no competing interests.

## Supplementary Material

Supplementary file (PDF) **Supplementary 1.** Clinical trial details. **Supplementary 2.** Instruments used. **Supplementary 3.** ESAS-r Renal Permission Letter. **Supplementary 4.** Eq-5D -5L Permission Letter. **Supplementary 5.** Advance care planning questionnaire (English). **Supplementary 6.** Symptoms algortihms. **Supplementary 7.** Interview guide for advance care planning discussion in ESKD setting (English). **Supplementary 8.** Pairwise comparison of Symptom burden, quality of life and illness-information preferences and ACP- decision-making preferences between intervention and control groups in patients with kidney failure. **Supplementary 9.** CONSORT 2025 checklist of information to include when reporting a randomised trial.

## Footnotes

Supplementary file (PDF)
**Supplementary 1.** Clinical trial details. **Supplementary 2.** Instruments used. **Supplementary 3.** ESAS-r Renal Permission Letter. **Supplementary 4.** Eq-5D -5L Permission Letter. **Supplementary 5.** Advance care planning questionnaire (English). **Supplementary 6.** Symptoms algortihms. **Supplementary 7.** Interview guide for advance care planning discussion in ESKD setting (English). **Supplementary 8.** Pairwise comparison of Symptom burden, quality of life and illness-information preferences and ACP- decision-making preferences between intervention and control groups in patients with kidney failure. **Supplementary 9.** CONSORT 2025 checklist of information to include when reporting a randomised trial.
