# Supplementary material for: Randomized Controlled Trial of Integrated Kidney Supportive Care in End-Stage Kidney Disease
Source: Kidney Int Rep. 2026 Jul 8;11(9):106696. doi: 10.1016/j.ekir.2026.106696 (PMC13474389; doi:10.1016/j.ekir.2026.106696)
Supplement: Supplementary file 1 [file mmc1.pdf]

Supplementary 1

Clinical Trial Details

**CTRI Number**	CTRI/2021/06/034179 [Registered on: 11/06/2021] - **Trial Registered Prospectively**
**Last Modified On**	19/06/2025
**Post Graduate Thesis**	Yes
**Type of Trial**	Interventional
**Type of Study**	Other (Specify) [Integrated Renal Supportive Care]
**Study Design**	Randomized, Parallel Group Trial
**Public Title of Study**	Effect of integrated focused renal supportive care on quality of life of kidney disease patients on hemodialysis.
**Scientific Title of Study**	Effect of Integrated Focused Renal Supportive Care on Symptom Burden, Quality of Life and Advance care planning in End stage Renal disease Patients: A Randomized Controlled Trial

Secondary IDs if Any

Secondary ID	Identifier
NIL	NIL

Details of Principal Investigator

Details of Principal Investigator	
**Name**	DrShankar Prasad N
**Designation**	Professor
**Affiliation**	Kasturba Medical College Manipal
**Address**	Department of Nephrology Kasturba hospital Manipal Tiger circle road Madhav Nagar Manipal 576104 Karnataka<br>Udupi<br>KARNATAKA<br>576104<br>India
**Phone**	9739905443
**Fax**	
**Email**	shankar.prasad@manipal.edu

Details Contact Person (Scientific Query)

Details Contact Person (Scientific Query)	
**Name**	DrShankar Prasad N
**Designation**	Professor
**Affiliation**	Kasturba Medical College Manipal
**Address**	Department of Nephrology Kasturba hospital Manipal Tiger circle road Madhav Nagar Manipal 576104 Karnataka<br>Udupi<br>KARNATAKA<br>576104<br>India
**Phone**	9739905443
**Fax**	
**Email**	shankar.prasad@manipal.edu

Details Contact Person (Public Query)

Details Contact Person (Public Query)	
**Name**	DrShankar Prasad N

**Designation**	Professor
**Affiliation**	Kasturba Medical College Manipal
**Address**	Department of Nephrology Kasturba hospital Manipal Tiger circle road Madhav Nagar Manipal 576104 Karnataka<br>Udupi<br>KARNATAKA<br>576104<br>India
**Phone**	9739905443
**Fax**	
**Email**	shankar.prasad@manipal.edu

Source of Monetary or Material Support

Source
nil

Primary Sponsor

Primary Sponsor Details	
**Name**	ICMR
**Address**	Indian Council of Medical Research New Delhi
**Type of Sponsor**	Government funding agency

Details of Secondary Sponsor

Name	Address
NIL	NIL

Countries of Recruitment

List of Countries
India

Sites of Study

Principal Investigator	Site Name	Address	Contact
Bharathi	Kasturba Hospital, Manipal	Dialysis Unit, Nephrology OPD, Department of Nephrology<br>Udupi, KARNATAKA	9008168494

Details of Ethics Committee

Name	Approval Status
Kasturba Medical college and kasturba Hospital Institutional Ethics Committee	Approved

Regulatory Clearance Status from DCGI

Status
Not Applicable

Health Condition / Problems Studied

Health Type	Condition
Patients	End stage renal disease

Intervention / Comparator Agent

Type	Name	Details
Intervention	Integrated Renal Supportive care	Patients in this arm with receive additional integrated renal supportive care services (intervention under study) by palliative care team. Patient in this arm will also receive ongoing care by Nephrologist and renal replacement therapy teams. Intervention will consist of total 06 month duration including follow ups
Comparator Agent	Standard of care Arm	Patient in control group will receive standard of care by the

		Nephrologist and Renal replacement teams.

Inclusion Criteria

Inclusion Criteria	
**Age From**	18.00 Year(s)
**Age To**	99.00 Year(s)
**Gender**	Both
**Details**	For phase 1<br>All ESRD patients who are more than 18 years old visiting the hospital<br><br>Patient Caregiver<br>Inclusion criteria Who are older than 18 years<br>Primary caregiver cared for at least 6 month<br><br>Nephrology Team<br>Inclusion Criteria Dialysis technicians dialysis nurses clinical faculties nephrologists who are involved in the care of ESRD patients for at least 12 months<br><br>For phase 2<br>Patients fulfilling the following criteria will be eligible for Phase II<br>Patient who are more than 18 years with a score <70 in modified Karnofsky performance Scale

Exclusion Criteria

Exclusion Criteria	
**Details**	For phase 1<br>Acute kidney injury patient in a tertiary care hospital<br>A patient who is unable to complete questionnaires<br><br>A patient caregiver who is unable to complete questionnaires<br>Those who are not willing to give consent

Method of Generating Random Sequence

Computer generated randomization

Method of Concealment

On-site computer system

Blinding/Masking

Not Applicable

Primary Outcome

Outcome	Timepoints
Quality of life and Symptom burden among End stage renal disease patients.<br>The number of patients and caregivers with knowledge of advanced care planning.<br>The factors related to acceptance or unwillingness for ACP discussions.	3 year

Secondary Outcome

Outcome	Timepoints
Change in quality of life measured using EQ -5D -5L 06 months after randomization.<br>Change in quality of life measured using EQ 5D 5L 02 and 04 months after randomization.<br>Changes in symptom burden using the Edmonton symptom assessment scale- renal version (ESAS-r)- renal 2, 4, and 6 months after randomization.<br>The difference in the number of a patient completing advanced care planning discussion and preparedness for ACP using dyad congruence.	3 years

Target Sample Size

Total Sample Size: **542**
Sample Size from India: **542**
Final Enrollment (Total): **Applicable only for Completed/Terminated trials**
Final Enrollment (India): **Applicable only for Completed/Terminated trials**

Phase of Trial

N/A

**Date of First Enrollment (India)**	15/07/2021
**Date of First Enrollment (Global)**	No Date Specified
**Date of Study Completion (India)**	No Date Specified
**Date of Study Completion (Global)**	No Date Specified
**Estimated Duration of Trial**	Years: 3, Months: 0, Days: 0
**Recruitment Status (Global)**	Not Applicable
**Recruitment Status (India)**	Open to Recruitment
**Publication Details**	3
**Brief Summary**	ESRD patient required Renal Replacement Therapies but these procedures impose technical & economic burden along with mental health & physical health deterioration, which affects patients quality of life & increase symptom burden.<br><br>Even though the burden of ESRD is high in the Indian scenario the role of focused integrated renal supportive care remains unexplored.<br><br>Family discussion and advance care planning may help in realistic intervention better quality of life care & family satisfaction.<br><br>There is a dearth of literature on effect of focused quality of life interventions like renal supportive care among the Indian population.

Acknowledgement Number	REF/2021/04/043294

CTRI Clarifications

CTRI Query Date	CTRI Query	Registrant Reply Date	Registrant Reply Details	Attached File
07/06/2021	Unlocked as per request	09/06/2021	Target Sample Size :<br>Phase-1<br><br>246- ESRD patients<br>246- Caregiver<br>50- Nephrology Team(Health care worker)<br><br>Total : 542<br><br>Phase-2<br>90 - samples in each group<br>Total : 180	No File Uploaded

Latest Data

Target Sample Size (last updated on:- 2021-06-08 15:01:36)	**Global Sample Size=**"542"<br>**Sample Size from India=**"542"

History of changes

Target Sample Size	Date of Change	Total Sample Size	Sample Size from India	Action Done
	08/06/2021	246	246	Record Modification

Objective :-: To study the effect of integrated focused renal supportive care on symptom burden, quality of life and advance care planning in end stage renal disease patients.

Primary objective

To evaluate the effect of focused renal supportive care on quality of life in ESRD patients.

Secondary objectives

To evaluate the effect of focused renal supportive care on symptom control in ESRD patients

To evaluate the effect of focused renal supportive care intervention on the willingness to document advance care planning.

Project Summary : End Stage Renal Disease(ESRD) is a life-threatening condition. End stage renal disease patients have physical and psychological symptom burdens similar to or more than that of advanced cancer patients. Dialysis can extend patient lives, but it limits the quality of survival time, due to high symptom burden, both physical and psychological, with low socioeconomic status. Having advance care planning (ACP) is a process that involves patients and their family can discuss with clinicians regarding current and future health status and treatment options and they can identify their health priorities and end of the life decision. It also helps to improve the end of life by reducing financial expenses associated with unwanted aggressive treatment near to death. So incorporating renal supportive care & advance care plan in the ESRD population can significantly improve quality of life and patient care.

The integrated renal supportive care is not developed in India. So we plan to conduct this study to identify the quality of life, symptom burden, knowledge, and acceptance of ACP in the ESRD population. Determine the impact of renal supportive care on symptoms burden and quality of life compared with usual care in the ESRD population.

Keywords : palliative care, end stage renal disease, Hemodialysis, advance care planning, Renal supportive care, quality of life

Methodology :

Study Design: Randomized Controlled Trial

Study Site: Renal Supportive Care Unit at Dialysis Unit Kasturba Hospital, Kasturba Medical College, Manipal,

Inclusion Criteria

Patients fulfilling the following criteria will be eligible

Patient who are more than 18 years with a score <70 in modified Karnofsky performance Scale

Patient with a score ≥of 4 for any symptom on ESAS.(Moderate-, severe symptoms)

Patient indicating level ≥3 on any of 5 dimensions of EQ- 5D- 5L.(Moderate/ severe/ extreme problems in any dimension of quality of life).

Exclusion Criteria

A patient who is unable to complete questionnaires due to physical and cognitive limitation.

Sample Size calculation:

$$n = \frac{2(Z_{1-\alpha/2} + Z_{1-\beta})^2}{\Phi d^2}$$

Φd is cohen's d effect size = 0.5

$$= \frac{2(1.96 + 0.84)^2}{(0.5)^2}$$

$$= 63$$

30% drop out

$$n = \frac{63}{1-0.3}$$

$$n = \frac{63}{0.7}$$

$$= 90$$

90 samples in each group with a 30 % drop out rate. Based on Cohen's d effect size

Sample size computation based on repeated measures is always less than sample size based on independent sample T-test or Cohen's d effect size

Procedure :

Patients fulfilling inclusion criteria will be asked to participate after providing written informed consent, over 24 months. Immediately after randomization using computer-generated random

numbers, patients in the intervention arm will be referred to the Renal supportive care unit and will receive integrated focused renal supportive care services. Patients in both arms will be evaluated at baseline (0), 2 monthly thereafter for 06 months. The evaluation will consist of filling ESAS-r and EQ 5D 5L questionnaire, and ACP documentation. Patients who are unable to return to the hospital will be contacted by telephone for completing questionnaires.

INTERVENTION

Name: Focused Kidney Supportive Care Interventions:

Rationale: According to the KDIGO guidelines, kidney supportive care can enhance the quality of life for individuals with end-stage kidney disease.

These patients received the following services:

- A detailed weekly medical assessment and focused symptomatic management were provided by a palliative care physician.
- The medical assessment included the documentation of comorbidities, past medical history, current complaints, and ongoing treatments. Following this comprehensive evaluation, all patients received a weekly symptom burden assessment. Patients with moderate to severe symptoms were reviewed weekly by a palliative care physician until their symptoms were adequately managed. An evidence-based, peer-reviewed algorithm for symptom management was developed to guide this process. Effective symptom control was expected to enhance both the physical and the psychological aspects of quality of life in the intervention group.
- The medical social worker provided two monthly psychological and social assessments, along with psychosocial support, including support group meetings.
    - ➢ A medical social worker assesses a patient's psychological and social needs through face-to-face meetings and the use of standardised questionnaires, including the Patient Health Questionnaire-9 (PHQ-9), Multidimensional Scale of Perceived Social Support (MSPSS), and Generalised Anxiety Disorder-7 (GAD-7). This included assessing the knowledge of the disease and its treatment options, patients’ and caregivers’ social support systems, financial stability, and any psycho-socio-spiritual distress.
- For Non-pharmacological management for symptom control and caregiver empowerment, patient leaflets were given in the local language.
- Two Family meetings, which include goal-of-care discussions with patients and carers, were held. A family meeting helps raise awareness of advance care planning.
    - ➢ The family meeting lasted for 45 minutes and included both the patient and their primary carers. The patient was asked about awareness of the diagnosis and prognosis. The patients’ chosen treatment options for continuing and future treatment were explored sensitively. If the patient agreed to consider advance care planning, they were asked to choose a healthcare proxy if they were unable to make a decision. The patient's preference for aggressive measures such as resuscitation, mechanical breathing, intensive care unit (ICU) admission, and dialysis was recorded.
    - ➢ One to two weeks after the initial family meeting, a second 15-minute follow-up meeting was scheduled to address any necessary clarifications.

Intervention provider: The kidney supportive care team consisted of nephrologists, palliative care physicians, a kidney supportive care coordinator and medical social workers with work experience in nephrology and palliative care.

Mode of delivery: Face-to-face interviews and assessments, printed materials, and telephone calls to ensure adherence to the intervention.

Location: Dialysis room, kidney supportive care clinic

When and how much: Patients were assessed weekly for symptom control and two monthly for psychological assessment, and twice during the study period for discussion of goals of care.

Tailoring: Interventions were tailored based on ESAS-r scores and quality-of-life domains using standardised symptom management algorithms and medical interventions

Adherence to the intervention: Two monthly audits and reviews of clinical notes and documents were conducted by independent team members from the Departments of Palliative Medicine and Nephrology.

Control Arm

Control group patients will receive existing standard renal care by primary nephrologist.

Follow-up Assessments

Patients in both arms will be assessed for QoL scores and symptom burden using EQ 5D 5L and ESAS-r renal scales at 2 months, 4 months, and 06 months. Patients who are unable to return to the hospital will be contacted by telephone for completing questionnaires. Patients will be asked about knowledge of the disease and their preferences for the treatment and documentation regarding ACP.

Expected Outcome

Proposed Outcomes:

Primary Outcome:

•Change in quality of life measured using EQ -5D -5L 06 months after randomization.

Secondary Outcome:

•Change in quality of life measured using EQ 5D 5L 02 and 04 months after randomization.

•Changes in symptom burden using the Edmonton symptom assessment scale- renal version (ESAS-r)- renal 2, 4, and 6 months after randomization.

•The difference in the number of patients' willingness to advance care planning .

Supplementary – 2

Instruments Used

EuroQol 5-Dimension 5-Level(EQ-5D-5L): It measures quality of life across five domains—mobility, self-care, usual activities, pain/discomfort, and anxiety/depression—each rated on five levels from 1 (no problems) to 5 (extreme problems), with higher levels indicating poorer health status.

Ref: Herdman M, Gudex C, Lloyd A, et al. Development and preliminary testing of the five-level version of EQ-5D (EQ-5D-5L). Qual Life Res. 2011;20(10):1727–1736.

Edmonton Symptom Assessment System–Revised, Renal(ESAS-r Renal): It consists of 11 items, including 10 standard symptom domains and one patient-identified symptom, each scored from 0 to 10; higher scores indicate greater symptom burden, categorised as mild (1–3), moderate (4–6), and severe (7–10).

Ref: Naik B, Nagaraju SP, Guddattu V, Salins N, Prabhu R, Damani A. Kannada translation and validation of the ESAS-r Renal for symptom burden in end-stage kidney disease. Indian J Palliat Care. 2023;29(2):195–199.

Davison SN, Jhangri GS, Johnson JA. Longitudinal validation of a modified Edmonton Symptom Assessment System in haemodialysis patients. Nephrol Dial Transplant. 2006;21(11):3189–3195

Advance Care Planning (ACP) Questionnaire: This tool assesses patients' willingness to engage in ACP discussions and document, including domains on illness-information preferences and decision-making; responses are measured on Likert scales, with higher scores indicating greater engagement.

Ref: Naik B, Singhai P, Damani A, et al. Development and validation of a questionnaire to explore advance care planning preferences in end-stage kidney disease. Palliat Support Care. 2024 Oct;22(5):1232-1236

Patient Health Questionnaire-9 (PHQ-9): It is a 9-item scale assessing depressive symptoms, with each item scored from 0 (not at all) to 3 (nearly every day), giving a total score range of 0–27; higher scores indicate greater depression severity.

Ref: Kroenke K, Spitzer RL, Williams JBW. The PHQ-9: validity of a brief depression severity measure. J Gen Intern Med. 2001;16(9):606–613.

Generalised Anxiety Disorder-7(GAD-7): It is a 7-item scale with total scores ranging from 0 to 21, where higher scores indicate greater anxiety severity, with cut-offs of 5, 10, and 15 for mild, moderate, and severe anxiety.

Ref: Spitzer RL, Kroenke K, Williams JBW, Löwe B. A brief measure for assessing generalized anxiety disorder: the GAD-7. Arch Intern Med. 2006;166(10):1092–1097.

Multidimensional Scale of Perceived Social Support (MSPSS): It consists of 12 items assessing support from family, friends, and significant others, scored on a 7-point Likert scale, with higher scores indicating greater perceived social support 30,31.

Ref: Zimet, G. D., Dahlem, N. W., Zimet, S. G., & Farley, G. K. (1988).
The multidimensional scale of perceived social support. *Journal of Personality Assessment, 52*(1), 30–41

Australia-modified Karnofsky Performance Status (AKPS) scale

The Australia-modified Karnofsky Performance Status (AKPS) scale (score range 0–100) rates functional status in 10-point increments, with higher scores indicating better independence and lower scores indicating greater functional impairment. Based on the score obtained, functional status can be interpreted as follows:

- 100–80: Normal functional capacity or mild symptoms; patients are independent and able to carry out routine activities.
- 70–60: Moderate functional limitation; patients require occasional assistance but can perform most self-care activities.
- 50–40: Significant impairment; patients require considerable assistance and spend increased time in bed.
- 30–10: Severe functional impairment; patients are predominantly bedbound and dependent on others for care.
- 0: Death

Ref: Abernethy AP, Shelby-James T, Fazekas BS, Woods D, Currow DC. The Australia-modified Karnofsky Performance Status (AKPS) scale: a revised scale for contemporary palliative care clinical practice.BMC Palliat Care. 2005;4:7

Supplementary 3

ESAS-r Renal Permission Letter

Re: Permission to use ESAS-r for research purpose

Sara Davison <sara.davison@ualberta.ca>

Sat 04/05/2019 13:31

To: Pankaj Singhai [MAHE-KMC] <pankaj.singhai@manipal.edu>;

I am happy for you to use the ESAS-r:Renal for your research.

Best wishes

Sara

On Sat., May 4, 2019, 1:51 a.m. Pankaj Singhai [MAHE-KMC], <pankaj.singhai@manipal.edu> wrote:

> Dear Dr Davidson,
> Greetings from Manipal, India.
> I am interested in doing a translation and validation of IPOS renal in Indian population. I wanted to use ESAS renal tool for this purpose. I request you to kindly permit us to use this tool for our research. This research is for academic and patient care and not for any monetary benefit. Your contribution will be appropriately aknowledged in any publication/ presentation arising out of this study.
> Thank you and regards
>
> Dr Pankaj Singhai
>
> Assistant Professor
>
> Department of Palliative Medicine and Supportive Care
>
> KMC Manipal
>
> +91 9920828452
>
> 0820-29-22026
>
> ***"No act of kindness, no matter how small, is ever wasted."***

ESAS-r: Renal -English Version

Edmonton Symptom Assessment System Revised: Renal (ESAS-r: Renal)

Please circle the number that best describes how you feel NOW:

No Pain	**0**	**1**	**2**	**3**	**4**	**5**	**6**	**7**	**8**	**9**	**10**	Worst Possible Pain
No Tiredness<br>*(Tiredness = lack of energy)*	**0**	**1**	**2**	**3**	**4**	**5**	**6**	**7**	**8**	**9**	**10**	Worst Possible Tiredness
No Drowsiness<br>*(Drowsiness = feeling sleepy)*	**0**	**1**	**2**	**3**	**4**	**5**	**6**	**7**	**8**	**9**	**10**	Worst Possible Drowsiness
No Nausea	**0**	**1**	**2**	**3**	**4**	**5**	**6**	**7**	**8**	**9**	**10**	Worst Possible Nausea
No Lack of Appetite	**0**	**1**	**2**	**3**	**4**	**5**	**6**	**7**	**8**	**9**	**10**	Worst Possible Lack of Appetite
No Shortness of Breath	**0**	**1**	**2**	**3**	**4**	**5**	**6**	**7**	**8**	**9**	**10**	Worst Possible of Shortness of Breath
No Depression<br>*(Depression = feeling sad)*	**0**	**1**	**2**	**3**	**4**	**5**	**6**	**7**	**8**	**9**	**10**	Worst Possible Depression
No Anxiety<br>*(Anxiety = feeling nervous)*	**0**	**1**	**2**	**3**	**4**	**5**	**6**	**7**	**8**	**9**	**10**	Worst Possible Anxiety
Best Wellbeing<br>*(Wellbeing = how you feel overall)*	**0**	**1**	**2**	**3**	**4**	**5**	**6**	**7**	**8**	**9**	**10**	Worst Possible Wellbeing
No Itching	**0**	**1**	**2**	**3**	**4**	**5**	**6**	**7**	**8**	**9**	**10**	Worst Possible Itching
No Problem Sleeping	**0**	**1**	**2**	**3**	**4**	**5**	**6**	**7**	**8**	**9**	**10**	Worst Possible Problem Sleeping
No ___________<br>Other Problem *(for example constipation)*	**0**	**1**	**2**	**3**	**4**	**5**	**6**	**7**	**8**	**9**	**10**	Worst Possible<br>________________

Patient's Name ____

Date______________________Time _____________________

Completed by (check one):

- ☐ Patient
- ☐ Family caregiver
- ☐ Health care professional caregiver
- ☐ Caregiver-assisted

Please mark on these pictures where it is that you hurt:

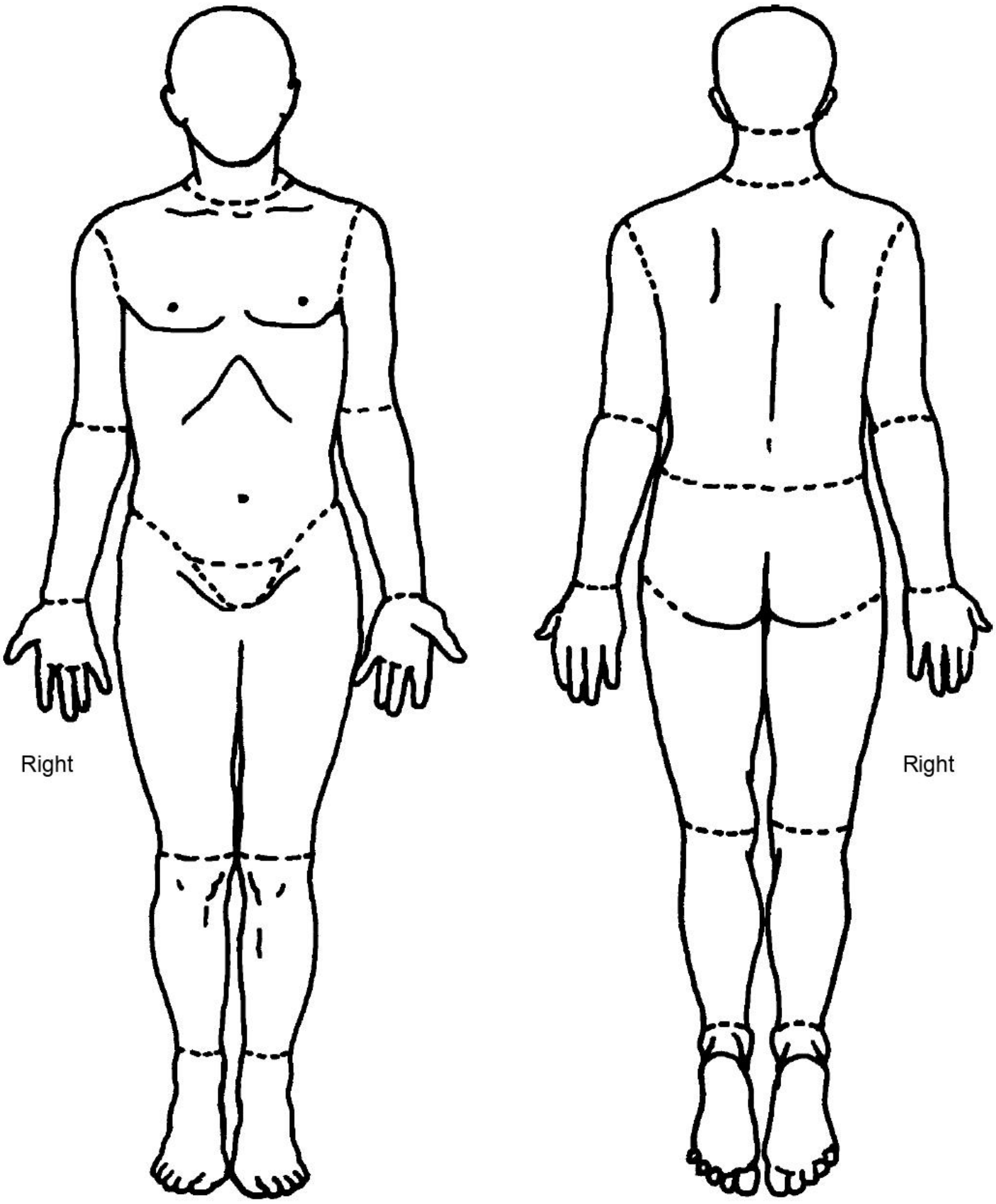

ESAS-r Renal – Kannada Version

ಎಡ್ಮಂಟನ್ ಸಿಂಪ್ಟಮ್ ಅಸೆಸ್ಮೆಂಟ್ ಸಿಸ್ಟಮ್
ರಿವೈಸ್ : ರಿನಲ್ [ಇಎಸ್ಎಎಸ್-ರ್: ರಿನಲ್]

ನಿಮಗೆ ಈಗ ಹೇಗೆ ಭಾಸವಾಗುವುದೆಂದು ಉತ್ತಮವಾಗಿ ವಿವರಿಸುವ ಸಂಖ್ಯೆಯನ್ನು ದಯವಿಟ್ಟು ವೃತ್ತಿಸಿ

ನೋವು ಇಲ್ಲ	0	1	2	3	4	5	6	7	8	9	10	ಕೆಟ್ಟದಾದ ನೋವು ಇದೆ
ಆಯಾಸ ಇಲ್ಲ	0	1	2	3	4	5	6	7	8	9	10	ಕೆಟ್ಟದಾದ ಆಯಾಸ ಇದೆ
ತೂಕಡಿಕೆಯ ಭಾವನೆ ಇಲ್ಲ	0	1	2	3	4	5	6	7	8	9	10	ಕೆಟ್ಟದಾದ ತೂಕಡಿಕೆಯ ಭಾವನೆ ಇದೆ
ವಾಕರಿಕೆ ಇಲ್ಲ	0	1	2	3	4	5	6	7	8	9	10	ಕೆಟ್ಟದಾದ ವಾಕರಿಕೆ ಇದೆ
ಹಸಿವು ಇದೆ	0	1	2	3	4	5	6	7	8	9	10	ಹಸಿವು ಇಲ್ಲ
ಉಸಿರಾಟದ ತೊಂದರೆ ಇಲ್ಲ	0	1	2	3	4	5	6	7	8	9	10	ಕೆಟ್ಟದಾದ ಉಸಿರಾಟದ ತೊಂದರೆ ಇದೆ
ಖಿನ್ನತೆ ಇಲ್ಲ	0	1	2	3	4	5	6	7	8	9	10	ಕೆಟ್ಟದಾದ ಖಿನ್ನತೆ ಇದೆ
ಆತಂಕ ಇಲ್ಲ	0	1	2	3	4	5	6	7	8	9	10	ಕೆಟ್ಟದಾದ ಆತಂಕ ಇದೆ
ಉತ್ತಮ ಯೋಗಕ್ಷೇಮ	0	1	2	3	4	5	6	7	8	9	10	ಕೆಟ್ಟದಾದ ಯೋಗಕ್ಷೇಮ
ತುರಿಕೆ ಇಲ್ಲ	0	1	2	3	4	5	6	7	8	9	10	ಕೆಟ್ಟದಾದ ತುರಿಕೆ ಇದೆ
ನಿದ್ರಾಹೀನತೆ ಇಲ್ಲ	0	1	2	3	4	5	6	7	8	9	10	ಕೆಟ್ಟದಾದ ನಿದ್ರಾಹೀನತೆ ಇದೆ
ಇತರೆ ಸಮಸ್ಯೆಗಳು____ (ಉದಾ: ಮಲಬದ್ಧತೆ.........)	0	1	2	3	4	5	6	7	8	9	10	ಕೆಟ್ಟದಾದ ತೊಂದರೆ ಇದೆ ____________

ರೋಗಿಯ ಹೆಸರು :

ದಿನಾಂಕ____________________________ಸಮಯ__________________________

(ಇವರಿಂದ ಪೂರ್ಣಗೊಂಡಿದೆ)

☐ ರೋಗಿ
☐ ಆರೈಕೆದಾರ
☐ ವೈದ್ಯಕೀಯ ವೃತ್ತಿಪರ ಸಹಾಯಕ
☐ ಆರೈಕೆದಾರ ಸಹಾಯ

ನಿಮಗೆ ನೋವು ಆದ ಸ್ಥಳವನ್ನು ದಯವಿಟ್ಟು ಈ ಚಿತ್ರದಲ್ಲಿ ಗುರುತಿಸಿ:[ಹಿಮ್ಮುಖ ಭಾಗದಲ್ಲಿ ದೇಹದ ರೇಖಾಚಿತ್ರವಿದೆ]

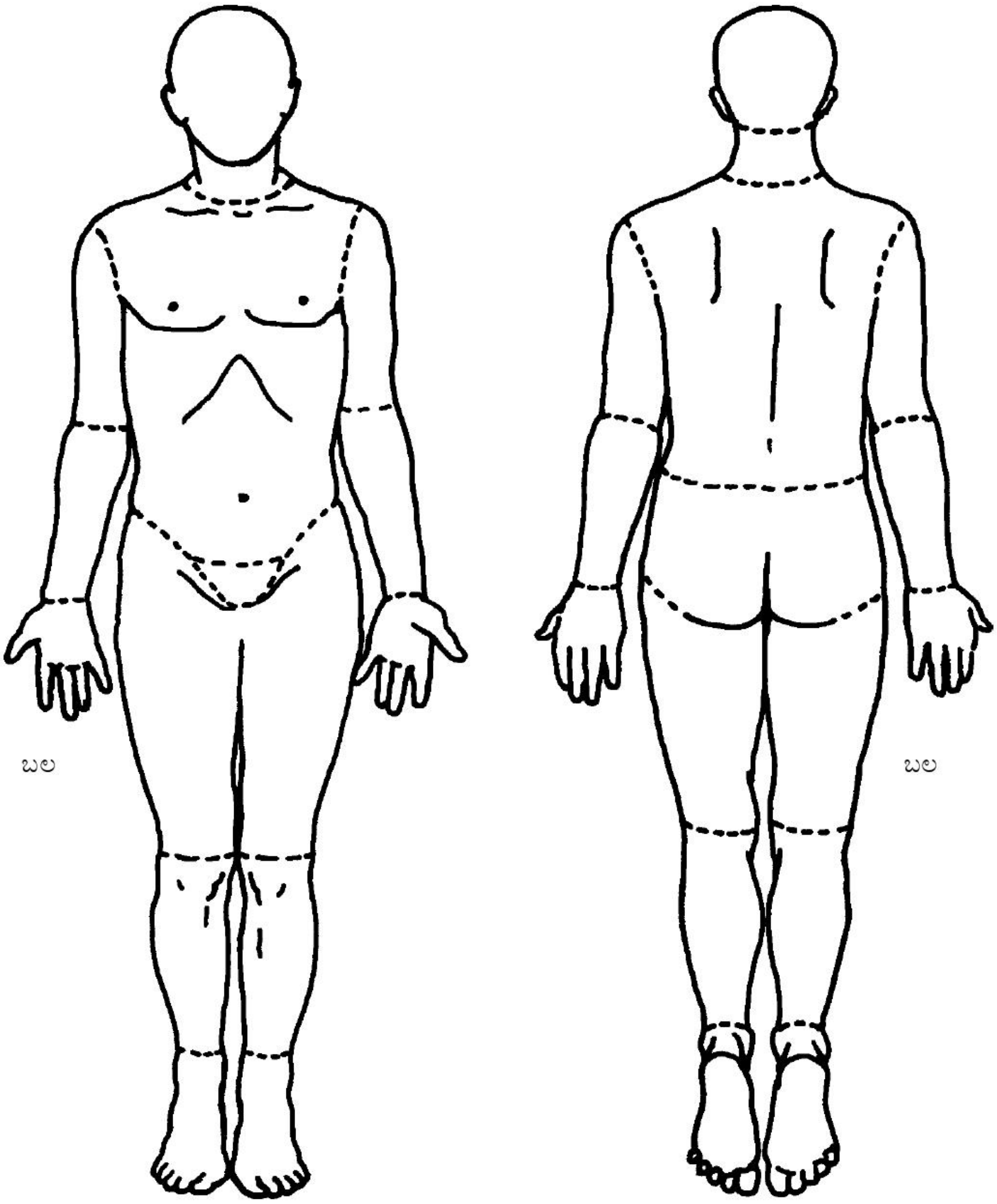

Supplementary 4

Eq-5D -5L Permission Letter

Reply all | Delete | Not junk | Block | ···

Version(s) Requested for the Opportunity MAHE, Manipal-TOU with Tracking Number: 38769

This message was identified as junk. It's not junk | Show blocked content

AV

Anameli Varela <varela@euroqol.org>
Thu 1/7/2021 6:34 PM
To: Bharathi [MAHE-MCHP]
Cc: registration@euroqol.org

euroqol logo

Dear Bharathi Naik ,

Thank you for your registration.

Please find below a link which gives you access to the requested Language Versions and the related available Translation Certificate(s).

Please be informed that it may take up to 10 minutes before you are able to access the requested Language Version(s). If after 10 minutes you still cannot access to Language Version(s), please reply to this email and we will assist you.

Click here to access your requested version(s)

Password to access the link: tzMuayFc

Best regards,

Anameli Varela

EuroQol Research Foundation

euroqol logo

T | E varela@euroqol.org | [www.euroqol.org]www.euroqol.org | Marten Meesweg 107 | 3068 AV Rotterdam The Netherlands

EQ-5D-5L Paper Self-Complete v1.0- English

Health Questionnaire

English version for India

India (English) © 2010 EuroQol Group EQ-5D™ is a trade mark of the EuroQol Group

Under each heading, please tick the ONE box that best describes your health TODAY.

MOBILITY

I have no problems in walking about ❑
I have slight problems in walking about ❑
I have moderate problems in walking about ❑
I have severe problems in walking about ❑
I am unable to walk about ❑

SELF-CARE

I have no problems in bathing or dressing myself ❑
I have slight problems in bathing or dressing myself ❑
I have moderate problems in bathing or dressing myself ❑
I have severe problems in bathing or dressing myself ❑
I am unable to bathe or dress myself ❑

USUAL ACTIVITIES *(e.g. work, study, housework, family or leisure activities)*

I have no problems doing my usual activities ❑
I have slight problems doing my usual activities ❑
I have moderate problems doing my usual activities ❑
I have severe problems doing my usual activities ❑
I am unable to do my usual activities ❑

PAIN / DISCOMFORT

I have no pain or discomfort ❑
I have slight pain or discomfort ❑
I have moderate pain or discomfort ❑
I have severe pain or discomfort ❑
I have extreme pain or discomfort ❑

ANXIETY / DEPRESSION

I am not anxious or depressed ❑
I am slightly anxious or depressed ❑
I am moderately anxious or depressed ❑
I am severely anxious or depressed ❑
I am extremely anxious or depressed ❑

India (English) © 2010 EuroQol Group EQ-5D™ is a trade mark of the EuroQol Group

- We would like to know how good or bad your health is TODAY.
- This scale is numbered from 0 to 100.
- 100 means the best health you can imagine.
  0 means the worst health you can imagine.
- Mark an X on the scale to indicate how your health is TODAY.
- Now, please write the number you marked on the scale in the box below.

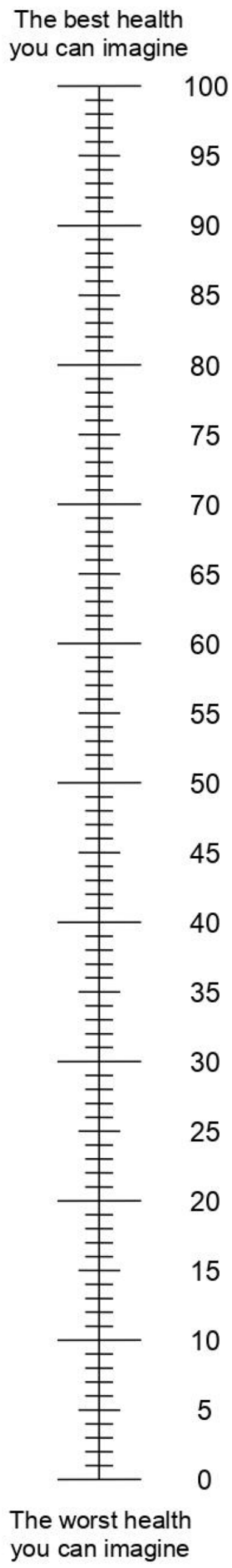

YOUR HEALTH TODAY = ☐

India (English) © 2010 EuroQol Group EQ-5D™ is a trade mark of the EuroQol Group

EQ-5D-5L Paper Self-Complete v1.0 - Kannada

ಆರೋಗ್ಯ ಪ್ರಶ್ನಾವಳಿ

□

(Kannada version for India)

India (Kannada) © 2010 EuroQol Group EQ-5D™ is a trade mark of the EuroQol Group

ಕೆಳಗಿನ ಪ್ರತಿಯೊಂದು ಶೀರ್ಷಿಕೆಯಡಿ, **ಇಂದಿನ** ನಿಮ್ಮ ಆರೋಗ್ಯವನ್ನು ಅತ್ಯುತ್ತಮವಾಗಿ ವಿವರಿಸುವ **ಒಂದು** ಬಾಕ್ಸ್ ಗೆ ಗುರುತು ಮಾಡಿ.

ಚಲನ ಶಕ್ತಿ

ಅತ್ತಿತ್ತ ನಡೆದಾಡುವಾಗ ನನಗೆ ಯಾವುದೇ ಸಮಸ್ಯೆಗಳು ಬರುವುದಿಲ್ಲ ❑
ಅತ್ತಿತ್ತ ನಡೆದಾಡುವಾಗ ನನಗೆ ಸ್ವಲ್ಪ ಸಮಸ್ಯೆಗಳು ಆಗುತ್ತವೆ ❑
ಅತ್ತಿತ್ತ ನಡೆದಾಡುವಾಗ ನನಗೆ ಸಾಧಾರಣವಾದ ಸಮಸ್ಯೆಗಳು ಆಗುತ್ತವೆ ❑
ಅತ್ತಿತ್ತ ನಡೆದಾಡುವಾಗ ನನಗೆ ತೀವ್ರವಾದ ಸಮಸ್ಯೆಗಳು ಆಗುತ್ತವೆ ❑
ನನಗೆ ಅತ್ತಿತ್ತ ನಡೆದಾಡಲು ಸಾಧ್ಯವಾಗುವುದಿಲ್ಲ ❑

ಸ್ವಯಂಜಾಗ್ರತೆ

ನನ್ನ ಮೈತೊಳೆದುಕೊಳ್ಳುವಾಗ ಅಥವಾ ಉಡುಪು ತೊಟ್ಟುಕೊಳ್ಳುವಾಗ ನನಗೆ ಯಾವುದೇ ಸಮಸ್ಯೆಗಳು ಇಲ್ಲ ❑
ನನ್ನ ಮೈತೊಳೆದುಕೊಳ್ಳುವಾಗ ಅಥವಾ ಉಡುಪು ತೊಟ್ಟುಕೊಳ್ಳುವಾಗ ನನಗೆ ಸ್ವಲ್ಪ ಸಮಸ್ಯೆಗಳು ಆಗುತ್ತವೆ ❑
ನನ್ನ ಮೈತೊಳೆದುಕೊಳ್ಳುವಾಗ ಅಥವಾ ಉಡುಪು ತೊಟ್ಟುಕೊಳ್ಳುವಾಗ ನನಗೆ ಸಾಧಾರಣವಾದ ಸಮಸ್ಯೆಗಳು ಆಗುತ್ತವೆ ❑
ನನ್ನ ಮೈತೊಳೆದುಕೊಳ್ಳುವಾಗ ಅಥವಾ ಉಡುಪು ತೊಟ್ಟುಕೊಳ್ಳುವಾಗ ನನಗೆ ತೀವ್ರವಾದ ಸಮಸ್ಯೆಗಳು ಆಗುತ್ತವೆ ❑
ನನಗೆ ನಾನೇ ಮೈ ತೊಳೆದುಕೊಳ್ಳಲು ಅಥವಾ ಉಡುಪು ತೊಟ್ಟುಕೊಳ್ಳಲು ಸಾಧ್ಯವಾಗುವುದಿಲ್ಲ ❑

ವಾಡಿಕೆಯ ಚಟುವಟಿಕೆಗಳು *(ಉದಾ: ಕೆಲಸ, ಅಧ್ಯಯನ, ಮನೆಕೆಲಸ, ಕೌಟುಂಬಿಕ ಅಥವಾ ಬಿಡುವಿನ ಚಟುವಟಿಕೆಗಳು)*

ನನ್ನ ವಾಡಿಕೆಯ ಚಟುವಟಿಕೆಗಳನ್ನು ಮಾಡುವುದರಲ್ಲಿ ನನಗೆ ಯಾವುದೇ ಸಮಸ್ಯೆಗಳು ಇಲ್ಲ ❑
ನನ್ನ ವಾಡಿಕೆಯ ಚಟುವಟಿಕೆಗಳನ್ನು ಮಾಡುವಲ್ಲಿ ನನಗೆ ಸ್ವಲ್ಪ ಸಮಸ್ಯೆಗಳು ಆಗುತ್ತವೆ ❑
ನನ್ನ ವಾಡಿಕೆಯ ಚಟುವಟಿಕೆಗಳನ್ನು ಮಾಡುವಲ್ಲಿ ನನಗೆ ಸಾಧಾರಣವಾದ ಸಮಸ್ಯೆಗಳು ಆಗುತ್ತವೆ ❑
ನನ್ನ ವಾಡಿಕೆಯ ಚಟುವಟಿಕೆಗಳನ್ನು ಮಾಡುವಲ್ಲಿ ನನಗೆ ತೀವ್ರವಾದ ಸಮಸ್ಯೆಗಳು ಆಗುತ್ತವೆ ❑
ನನಗೆ ನನ್ನ ವಾಡಿಕೆಯ ಚಟುವಟಿಕೆಗಳನ್ನು ಮಾಡಲು ಸಾಧ್ಯವಾಗುವುದಿಲ್ಲ ❑

ನೋವು / ಅಸೌಖ್ಯ

ನನಗೆ ಯಾವುದೇರೀತಿಯ ನೋವು ಅಥವಾ ಅಸೌಖ್ಯ ಇಲ್ಲ ❑
ನನಗೆ ಸ್ವಲ್ಪ ನೋವು ಅಥವಾ ಅಸೌಖ್ಯ ಇದೆ ❑
ನನಗೆ ಸಾಧಾರಣವಾದ ನೋವು ಅಥವಾ ಅಸೌಖ್ಯ ಇದೆ ❑
ನನಗೆ ತೀವ್ರ ನೋವು ಅಥವಾ ಅಸೌಖ್ಯ ಇದೆ ❑
ನನಗೆ ವಿಪರೀತವಾದ ನೋವು ಅಥವಾ ಅಸೌಖ್ಯ ಇದೆ ❑

ಗಾಬರಿ / ಬೇಜಾರು

India (Kannada) © 2010 EuroQol Group EQ-5D™ is a trade mark of the EuroQol Group

ನಾನು ಗಾಬರಿ ಅಥವಾ ಬೇಜಾರು ಹೊಂದಿಲ್ಲ ❑
ನಾನು ಸ್ವಲ್ಪ ಗಾಬರಿ ಅಥವಾ ಬೇಜಾರು ಹೊಂದಿದ್ದೇನೆ ❑
ನಾನು ಸಾಧಾರಣ ಮಟ್ಟಿಗೆ ಗಾಬರಿ ಅಥವಾ ಬೇಜಾರು ಹೊಂದಿದ್ದೇನೆ ❑
ನಾನು ತೀವ್ರ ಗಾಬರಿ ಅಥವಾ ಬೇಜಾರು ಹೊಂದಿದ್ದೇನೆ ❑
ನಾನು ವಿಪರೀತ ಗಾಬರಿ ಅಥವಾ ಬೇಜಾರು ಹೊಂದಿದ್ದೇನೆ ❑

India (Kannada) © 2010 EuroQol Group EQ-5D™ is a trade mark of the EuroQol Group

ಇಂದಿನ ನಿಮ್ಮ ಆರೋಗ್ಯ = [ ]

- **ಇಂದು** ನಿಮ್ಮ ಆರೋಗ್ಯ ಎಷ್ಟು ಉತ್ತಮ ಅಥವಾ ಕೆಟ್ಟದಾಗಿದೆ ಎಂದು ತಿಳಿಯಲು ನಾವು ಇಚ್ಛಿಸುತ್ತೇವೆ.

- ಈ ಮಾಪಕವು 0 ಯಿಂದ 100 ರವರೆಗಿನ ಸಂಖ್ಯೆಗಳನ್ನು ಒಳಗೊಂಡಿದೆ.

- 100 ಎಂದರೆ ನೀವು ಕಲ್ಪಿಸಿಕೊಳ್ಳಬಹುದಾದ ಅತ್ಯುತ್ತಮ ಆರೋಗ್ಯ.
  0 ಎಂದರೆ ಎಂದರೆ ನೀವು ಕಲ್ಪಿಸಿಕೊಳ್ಳಬಹುದಾದ ಅತ್ಯಂತ ಕೆಟ್ಟ ಆರೋಗ್ಯ.

- **ಇಂದು** ನಿಮ್ಮ ಆರೋಗ್ಯ ಹೇಗಿದೆ ಎಂದು ಸೂಚಿಸಲು ಮಾಪಕದ ಮೇಲೆ X ಗುರುತು ಮಾಡಿ.

- ಈಗ, ನೀವು ಮಾಪಕದಲ್ಲಿ ಗುರುತಿಸಿದ ಸಂಖ್ಯೆಯನ್ನು ದಯವಿಟ್ಟು ಕೆಳಗಿನ ಬಾಕ್ಸಿನಲ್ಲಿ ಬರೆಯಿರಿ.

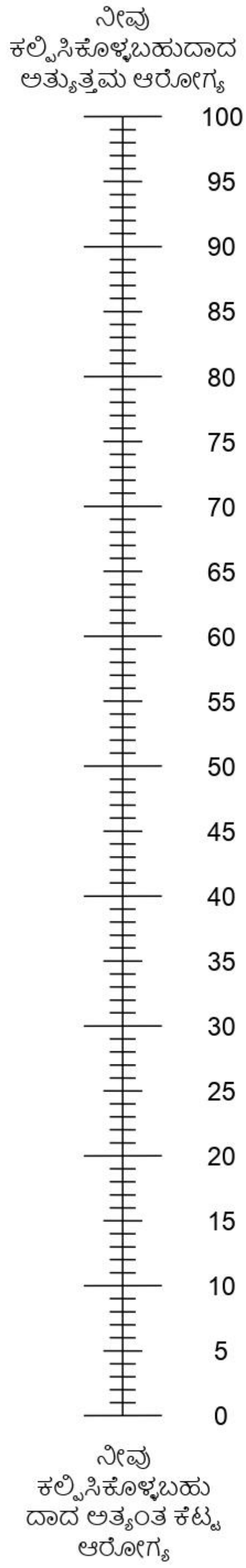

India (Kannada) © 2010 EuroQol Group EQ-5D™ is a trade mark of the EuroQol Group

Supplementary 5

Advance care planning questionnaire (English)

A. Patient Questionnaire

A1. Illness-information preferences

	Never	Rarely	Sometimes	Often	Always
A.1.1. Do you prefer to know about your illness?					
A.1.2. Do you prefer to know about the severity of your illness?					
A.1.3. Do you prefer to know about the future course of your illness?					
A.1.4. Do you prefer to know about your treatment options?					
A.1.5. Do you prefer to know the success/side-effects of your treatment options?					
A.1.6. Do you prefer to know about the future symptoms?					
A.1.7. Do you prefer to know about the future complications due to illness or due to its treatment?					
A.1.8. Do you prefer to know about your expected length of survival?					

A2. Decision-making preferences

	Strongly Disagree	Disagree	Undecided	Agree	Strongly Agree
A.2.1. Future health care preferences and options should be discussed and planned					
A.2.2. I prefer my future health care preferences to be documented as an advanced care plan					
A.2.3. I prefer to be part of all decision-making discussions concerning my current and future healthcare options					
A.2.4. I would prefer to make all the health care decisions myself					
A.2.5. I would prefer family to make all the health care decisions					
A.2.6. I would prefer health care provider (doctors/dialysis team) to make all the health care decisions					
A.2.7. I would prefer myself, my family, and healthcare providers (doctors/dialysis team) are involved in the decision-making process					
A.2.8. I like my preferences, decisions and wishes made about my future treatment and general care process is respected and implemented					

B. Caregiver Questionnaire

B1. Illness-information preferences

	Never	Rarely	Sometimes	Often	Always
B.1.1. Do you prefer for your patient/your relative to know about his/her illness?					
B.1.2. Do you prefer for your patient/your relative to know about the severity of his/her illness?					
B.1.3. Do you prefer your patient/your relative to know about the future course of his/her illness?					
B.1.4. Do you prefer your patient/your relative to know about his/her treatment options?					
B.1.5. Do you prefer your patient/your relative to know the success/side-effects of his/her treatment options?					
B.1.6. Do you prefer your patient/your relative to know about his/her future symptoms?					
B.1.7. Do you prefer your patient/your relative to know about his/her future complications due to illness and its treatment?					
B.1.8. Do you prefer your patient/your relative to know about his/her expected length of survival?					

B2. Decision-making preferences

	Strongly Disagree	Disagree	Undecided	Agree	Strongly Agree
B.2.1. Future health care preferences and options should be discussed and planned					
B.2.2. I prefer my patient's/ relative's future health care preferences should be documented as an advanced care plan					
B.2.3. I prefer my patient/relative to be part of all discussions concerning decision-making about his/her current and future healthcare options					
B.2.4. I would prefer the patient/my relative to make all the health care decisions					
B.2.5. I would prefer the family to make all the health care decisions					
B.2.6. I would prefer health care provider to make all the health care decisions					
B.2.7. I would prefer a shared decision-making process where patient(my relative), family, healthcare providers are involved in the decision-making process					
B.2.8. I like my patient's(relative's) preferences, decisions and wishes made about his/her future treatment and the general care process is respected and implemented					

Patient & Caregiver Advance care planning questionnaire in Kannada

A. ರೋಗಿಯ ಪ್ರಶ್ನಾವಳಿ:

A1. ಆನಾರೋಗ್ಯದ ಮಾಹಿತಿ ಆದ್ಯತೆಗಳು

	ಎಂದಿಗೂ ಇಲ್ಲ	ವಿರಳವಾಗಿ	ಕೆಲವೊಮ್ಮೆ	ಆಗಾಗ	ಯಾವಾಗಲೂ
A1.1. ನೀವು ನಿಮ್ಮ ಅನಾರೋಗ್ಯದ ಬಗ್ಗೆ ತಿಳಿದುಕೊಳ್ಳಲು ಬಯಸುತ್ತೀರಾ?					
A1.2. ನೀವು ನಿಮ್ಮ ಆನಾರೋಗ್ಯದ ಗಂಭೀರತೆತ ಬಗ್ಗೆ ತಿಳಿಯಾ ಬಯಸುತ್ತೀರಾ?					
A1.3. ನೀವು ನಿಮ್ಮ ಭವಿಷ್ಯದ ಆನಾರೋಗ್ಯದ ಬಗ್ಗೆ ತಿಳಿಯಾ ಬಯಸುತ್ತೀರಾ?					
A1.4. ನೀವು ನಿಮ್ಮ ರೋಗದ ಚಿಕಿತ್ಸೆಯ ಆಯ್ಕೆಗಳ ಬಗ್ಗೆ ತಿಳಿದುಕೊಳ್ಳಲು ಬಯಸುತ್ತೀರಾ?					
A1.5. ನೀವು ನಿಮ್ಮ ರೋಗದ ಚಿಕಿತ್ಸೆಯ ಆಯ್ಕೆಗಳ ಯಶಸ್ಸು/ ಅಡ್ಡಪರಿಣಾಮಗಳನ್ನು ತಿಳಿದುಕೊಳ್ಳಲು ಬಯಸುತ್ತೀರಾ?					
A1.6. ನೀವು ನಿಮ್ಮ ರೋಗದ ಭವಿಷ್ಯದ ಲಕ್ಷಣಗಳ ಬಗ್ಗೆ ತಿಳಿದುಕೊಳ್ಳಲು ಬಯಸುತ್ತಿರಾ?					
A1.7. ನೀವು ನಿಮ್ಮಅನಾರೋಗ್ಯದ ಅಥವಾ ಅದರ ಚಿಕಿತ್ಸೆಯ ಕಾರಣದಿಂದಾಗಿ ಭವಿಷ್ಯದಲ್ಲಾಗುವ ತೊಡಕುಗಳ ಬಗ್ಗೆ ತಿಳಿದುಕೊಳ್ಳಲು ಬಯಸುತ್ತೀರಾ?					
A1.8. ನಿಮ್ಮ ನಿರೀಕ್ಷಿತ ಜೀವಿತಾವಧಿ ಬಗ್ಗೆ ತಿಳಿದುಕೊಳ್ಳಲು ನೀವು ಬಯಸುತ್ತೀರಾ?					

A2. ನಿರ್ಧಾರ ತೆಗೆದುಕೊಳ್ಳುವ ಆದ್ಯತೆಗಳು

	ಖಂಡಿತವಾಗಿ ಒಪ್ಪುದಿಲ್ಲ	ಒಪ್ಪುದಿಲ್ಲ	ನಿರ್ಧರಿಸಿಲ್ಲ	ಒಪ್ಪುತ್ತೇನೆ	ಖಂಡಿತ ಒಪ್ಪುತ್ತೇನೆ
A2.1. ಭವಿಷ್ಯದ ಆರೋಗ್ಯದ ಕಾಳಜಿಯ ಆದ್ಯತೆಗಳು ಮತ್ತು ಆಯ್ಕೆಯನ್ನು ಚರ್ಚಿಸಬೇಕು ಮತ್ತು ಯೋಜಿಸಬೇಕು					
A2.2. ನನ್ನ ಭವಿಷ್ಯದ ಆರೋಗ್ಯ ಕಾಳಜಿ ಆದ್ಯತೆಗಳನ್ನು ಮುಂಗಡ ಆರೈಕೆ ಯೋಜನೆಯಂತೆ ದಾಖಲಿಸಬೇಕು ಎಂದು ನಾನು ಬಯಸುತ್ತೇನೆ					
A2.3. ನನ್ನ ಪ್ರಸ್ತುತ ಮತ್ತು ಭವಿಷ್ಯದ ಆರೋಗ್ಯದ ರಕ್ಷಣೆಯ ಆಯ್ಕೆಗಳ ಬಗ್ಗೆ ನಿರ್ಧಾರ ತೆಗೆದುಕೊಳ್ಳುವ ಎಲ್ಲಾ ಚರ್ಚೆಗಳಲ್ಲಿ ಭಾಗವಹಿಸಲು ನಾನು ಬಯಸುತ್ತೇನೆ					
A2.4. ನನ್ನ ಎಲ್ಲಾ ಆರೋಗ್ಯ ರಕ್ಷಣೆಯ ನಿರ್ಧಾರಗಳನ್ನು ನಾನು ತೆಗೆದುಕೊಳ್ಳಲು ಬಯಸುತ್ತೇನೆ					
A2.5. ನನ್ನ ಎಲ್ಲಾ ಆರೋಗ್ಯ ರಕ್ಷಣೆಯ ನಿರ್ಧಾರಗಳನ್ನು ತೆಗೆದುಕೊಳ್ಳಲು ನಾನು ಕುಟುಂಬಕ್ಕೆ ಆದ್ಯತೆ ನೀಡುತ್ತೇನೆ					
A2.6. ನನ್ನ ಎಲ್ಲಾ ಆರೋಗ್ಯ ರಕ್ಷಣೆಯ ನಿರ್ಧಾರ ತೆಗೆದುಕೊಳ್ಳಲು ಆರೋಗ್ಯ ಕಾರ್ಯಕರ್ತರಿಗೆ [ಡಯಾಲಿಸಿಸ್ ವೈದರು, ನರ್ಸ್ ಮತ್ತು ಟೆಕ್ನಿಷೀಯನ್] ಆದ್ಯತೆ ನೀಡುತ್ತೇನೆ.					
A2.7. ನಾನು ನನ್ನ ಕುಟುಂಬ ಹಾಗೂ ಆರೋಗ್ಯ ಕಾರ್ಯಕರ್ತರು ಎಲ್ಲಾರೂ ಕೂಡಿ ಆರೋಗ್ಯ ರಕ್ಷಣೆಯ ಬಗ್ಗೆ ನಿರ್ಧಾರ ತೆಗೆದುಕೊಳ್ಳಲು ಬಯಸುತ್ತೇನೆ					
A2.8. ಭವಿಷ್ಯದ ಆರೈಕೆ ಮತ್ತು ಸಾಮಾನ್ಯ ಆರೈಕೆಯ ಪ್ರತಿಕ್ರಿಯೆಯಲ್ಲಿ ನನ್ನ ಆದ್ಯತೆಗಳು, ನಿರ್ಧಾರಗಳು, ಆಸೆಗಳನ್ನು ಗೌರವಿಸಬೇಕು ಮತ್ತು ಕಾರ್ಯಗತಗೊಳಿಸಬೇಕು ಎಂದು ನಾನು ಬಯಸುತ್ತೇನೆ					

B. ಆರೈಕೆದಾರರ ಪ್ರಶ್ನಾವಳಿ:

B1. ಅನಾರೋಗ್ಯದ ಮಾಹಿತಿ ಆದ್ಯತೆಗಳು

	ಎಂದಿಗೂ ಇಲ್ಲ	ವಿರಳವಾಗಿ	ಕೆಲವೊಮ್ಮೆ	ಆಗಾಗ	ಯಾವಾಗಲೂ
B1.1. ನಿಮ್ಮ ರೋಗಿಯು ತನ್ನ ಅನಾರೋಗ್ಯದ ಬಗ್ಗೆ ತಿಳಿದುಕೊಳ್ಳಲು ಬಯಸುತ್ತೀರಾ?					
B1.2. ನಿಮ್ಮ ರೋಗಿಗೆ ಅವನ/ಅವಳ ಆನಾರೋಗ್ಯದ ಗಂಭೀರತೆಯ ಬಗ್ಗೆ ತಿಳಿಯಲು ನೀವು ಬಯಸುತ್ತೀರಾ?					
B1.3ನಿಮ್ಮ ರೋಗಿಯು ತನ್ನ ಆನಾರೋಗ್ಯದ ಭವಿಷ್ಯದ ಬಗ್ಗೆ ತಿಳಿದುಕೊಳ್ಳಲು ನೀವು ಬಯಸುತ್ತೀರಾ?					
B1.4 ನಿಮ್ಮ ರೋಗಿಗೆ ಅವನ/ಅವಳ ಚಿಕಿತ್ಸೆಯ ಆಯ್ಕೆಯ ಬಗ್ಗೆ ತಿಳಿಯಲು ನೀವು ಬಯಸುತ್ತೀರಾ?					
B1.5 ನಿಮ್ಮ ರೋಗಿಯು ಅವನ ಅಥವಾ ಅವಳ ಚಿಕಿತ್ಸೆಯ ಆಯ್ಕೆಗಳ ಯಶಸ್ಸು/ ಅಡ್ಡಪರಿಣಾಮಗಳನ್ನು ತಿಳಿಯಲು ನೀವು ಬಯಸುತ್ತೀರಾ?					
B1.6 ನಿಮ್ಮ ರೋಗಿಯು ತನ್ನ ರೋಗದ ಭವಿಷ್ಯದ ಲಕ್ಷಣಗಳ ಬಗ್ಗೆ ತಿಳಿದುಕೊಳ್ಳಲು ನೀವು ಬಯಸುತ್ತಿರಾ?					
B1.7. ನಿಮ್ಮ ರೋಗಿಯ ಅನಾರೋಗ್ಯ ಅಥವಾ ಅದರ ಚಿಕಿತ್ಸೆಯ ಕಾರಣದಿಂದಾಗಿ ಅವನ/ಅವಳ ಭವಿಷ್ಯದಲ್ಲಾಗುವ ತೊಡಕುಗಳ ಬಗ್ಗೆ ತಿಳಿದುಕೊಳ್ಳಲು ನೀವು ಬಯಸುತ್ತೀರಾ?					
B1.8. ನಿಮ್ಮ ರೋಗಿಯ ಅವನ/ಅವಳ ನಿರೀಕ್ಷಿತ ಜೀವಿತಾವಧಿ ಬಗ್ಗೆ ತಿಳಿದುಕೊಳ್ಳಲು ನೀವು ಬಯಸುತ್ತೀರಾ?					

B2. ನಿರ್ಧಾರ ತೆಗೆದುಕೊಳ್ಳುವ ಆದ್ಯತೆಗಳು

	ಖಂಡಿತವಾಗಿ ಒಪ್ಪುದಿಲ್ಲ	ಒಪ್ಪುದಿಲ್ಲ	ನಿರ್ಧರಿಸಿಲ್ಲ	ಒಪ್ಪುತ್ತೇನೆ	ಖಂಡಿತ ಒಪ್ಪುತ್ತೇನೆ
B2.1. ಭವಿಷ್ಯದ ಆರೋಗ್ಯ ಕಾಳಜಿ ಆದ್ಯತೆಗಳು ಮತ್ತು ಆಯ್ಕೆಗಳನ್ನು ಚರ್ಚಿಸಬೇಕು ಮತ್ತು ಯೋಜಿಸಬೇಕು					
B2.2. ನನ್ನ ರೋಗಿಯ ಭವಿಷ್ಯದ ಆರೋಗ್ಯ ಕಾಳಜಿ ಆದ್ಯತೆಗಳನ್ನು ಮುಂಗಡ ಆರೈಕೆ ಯೋಜನೆಯಂತೆ ದಾಖಲಿಸಬೇಕು ಎಂದು ನಾನು ಬಯಸುತ್ತೇನೆ					
B2.3. ನನ್ನ ರೋಗಿಯು ತನ್ನ ಪ್ರಸ್ತುತ ಮತ್ತು ಭವಿಷ್ಯದ ಆರೋಗ್ಯದ ರಕ್ಷಣೆಯ ಆಯ್ಕೆಗಳ ಬಗ್ಗೆ ನಿರ್ಧಾರ ತೆಗೆದುಕೊಳ್ಳುವ ಎಲ್ಲಾ ಚರ್ಚೆಗಳಲ್ಲಿ ಭಾಗವಹಿಸಲು ನಾನು ಬಯಸುತ್ತೇನೆ					
B2.4. ಎಲ್ಲಾ ಆರೋಗ್ಯ ರಕ್ಷಣೆಯ ನಿರ್ಧಾರಗಳನ್ನು ತೆಗೆದುಕೊಳ್ಳಲು ನಾನು ರೋಗಿಗೆ ಆದ್ಯತೆ ನೀಡುತ್ತೇನೆ					
B2.5. ಎಲ್ಲಾ ಆರೋಗ್ಯ ರಕ್ಷಣೆಯ ನಿರ್ಧಾರಗಳನ್ನು ತೆಗೆದುಕೊಳ್ಳಲು ನಾನು ಕುಟುಂಬಕ್ಕೆ ಆದ್ಯತೆ ನೀಡುತ್ತೇನೆ					
B2.6. ಎಲ್ಲಾ ಆರೋಗ್ಯ ರಕ್ಷಣೆಯ ನಿರ್ಧಾರಗಳನ್ನು ತೆಗೆದುಕೊಳ್ಳಲು ನಾನು ಆರೋಗ್ಯ ಕಾರ್ಯಕರ್ತರಿಗೆ [ಡಯಾಲಿಸಿಸ್ ವೈದರು, ನರ್ಸ್ ಮತ್ತು ಟೆಕ್ನಿಷೀಯನ್] ಆದ್ಯತೆ ನೀಡುತ್ತೇನೆ					
B2.7. ರೋಗಿ, ಕುಟುಂಬ ಹಾಗೂ ಆರೋಗ್ಯ ಕಾರ್ಯಕರ್ತರು ಎಲ್ಲರೂ ಕೂಡ ಆರೋಗ್ಯ ರಕ್ಷಣೆಯ ಬಗ್ಗೆ ನಿರ್ಧಾರ ತೆಗೆದುಕೊಳ್ಳುವ ಪ್ರಕ್ರಿಯೆಯಲ್ಲಿ ಭಾಗವಹಿಸಿ, ನಿರ್ಧಾರ ತೆಗೆದುಕೊಳ್ಳಲು ನಾನು ಬಯಸುತ್ತೇನೆ.					
B2.8. ನನ್ನ ರೋಗಿಯ ಭವಿಷ್ಯದ ಆರೈಕೆ ಮತ್ತು ಸಾಮಾನ್ಯ ಆರೈಕೆಯ ಪ್ರಕ್ರಿಯೆಯಲ್ಲಿ ಅವನ/ಅವಳ ಆದ್ಯತೆಗಳು, ನಿರ್ಧಾರಗಳು, ಆಸೆಗಳನ್ನು ಗೌರವಿಸಲಾಗುತ್ತದೆ ಮತ್ತು ಕಾರ್ಯಗತಗೊಳಿಸಲಾಗುತ್ತದೆ ಎಂದು ನಾನು ಬಯಸುತ್ತೇನೆ					

Supplementary -6

Symptoms Algorithms

Contents

Approach to Symptom Management in ESRD patients 2

Uraemic Pruritus 3

Restless Leg Syndrome.......... 5

Nausea /Vomiting 7

Nociceptive Pain 9

Bone Pain 10

Neuropathic Pain 11

Breathlessness 12

Sleeplessness 14

Fatigue 17

Nutritional concerns 18

Generalised Edema 19

Depression 20

Approach to Symptom Management in ESRD patients

- Empathetic communications, active listening
- Assess proactively
- Document symptoms with scores – monitor until relief
- Encourage questions and clarify
- Assess the symptom; acknowledge the distress and address both
- Routine use of validated Tools – iPOS Renal
- Evaluate and explain the contributors thoroughly
- Discuss & agree upon goals of care with the patient E.g. improvement in function
- Correct the correctable contributors
- Integrate non-pharmacological options early
- Employ MDT expertise
- Assist, arrange for support devices, aids for symptom relief, where indicated
- Consider availability, accessibility and pharmaco-economics of prescribed drugs
- Improve compliance by using the simplest prescription regime
- Monitor relief, and improved functions, prescription adherence, compliance, adverse effects

Uremic Pruritus

Ref: https://www.ncbi.nlm.nih.gov/pmc/articles/PMC5497472/; www.ckmcare.com; Am J Kidney Dis. 2017;70(5):638-655,

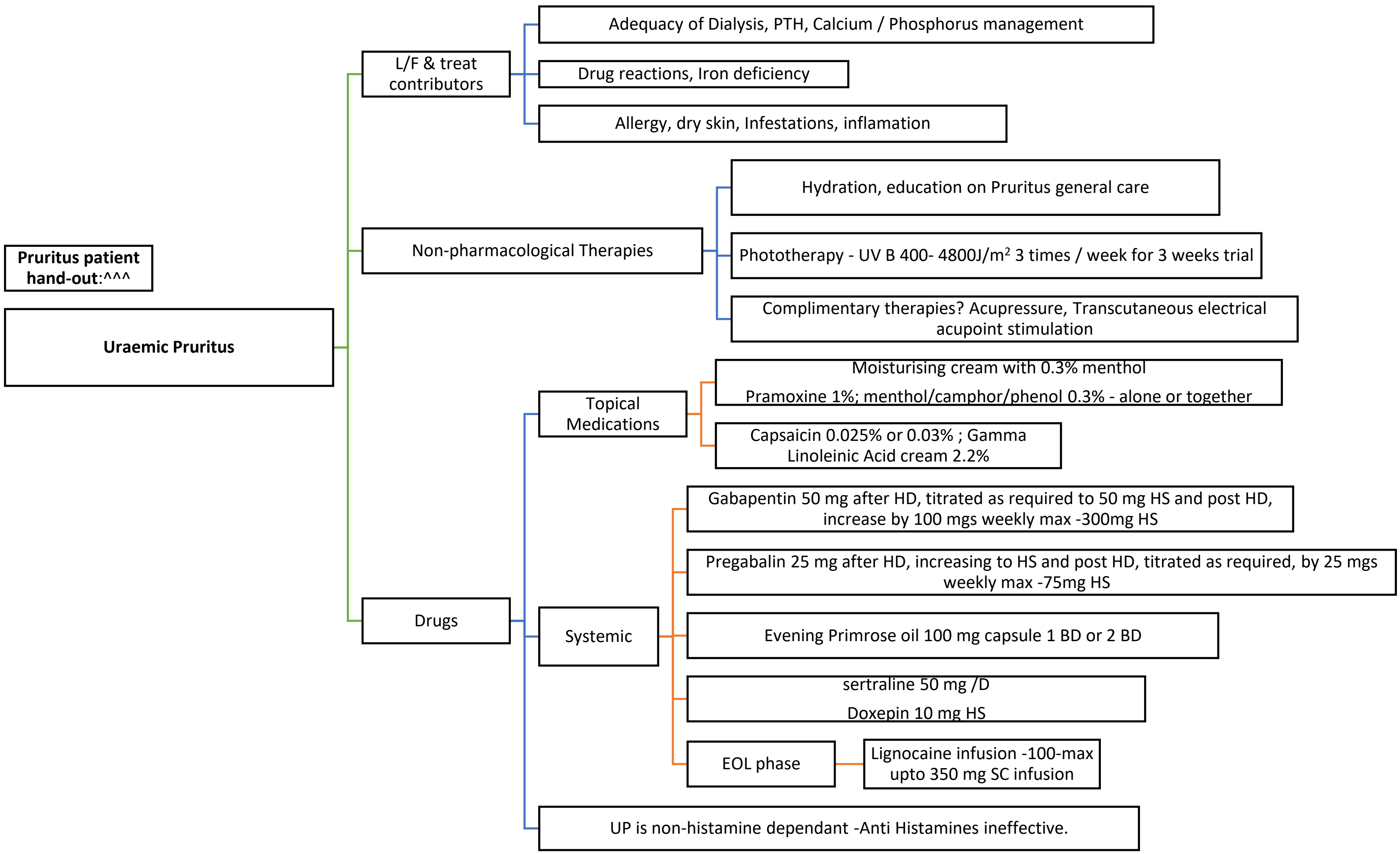

Pruritus Patient Hand-Out[1]

- Minimize scratching. Keep short fingernails. Try patting the skin instead of scratching. Try to avoid situations where you tend to scratch by observing habits.
- Avoid hot water baths for long periods as hot water may remove the natural oils from your skin. Bathe or shower quickly in tepid (not hot) water no more than once daily. Use soak baths in cool water.
- Use gentle soaps, limit soaping to axilla, groin. Avoid using soaps or foaming body washes even if they claim to be good for dry itchy skin. Many of these contain detergents which remove natural oil from the skin
- Pat dry the skin, quickly moisturize while still damp. Use airy, cool cottons to wear
- Keep your bedroom cool and do not sleep with heavy or heat-retaining bedclothes. Room moisturiser is helpful during dry seasons e.g. winter. If you are hot and itchy during the day, a fan can help to cool the skin down
- Aggressive moisturisation - People with pruritus usually have dry skin, and therefore moisturisers may help to soothe the dry skin. They ease itching, reduce scaling, soften cracked areas and help the penetration of other topical treatments. Use moisturisers which do not have perfumes, preservatives. Hydrate orally, as per urine out-put.
- For quick relief - You may find that laying a cool towel soaked in moisturising cream on the skin can reduce the feeling of itch. A cooled moisturiser which has been kept in the refrigerator may also help

[1] British Association of Dermatologists

Restless Leg Syndrome

Ref: CKMCare.com, International Restless Legs Syndrome Study Group

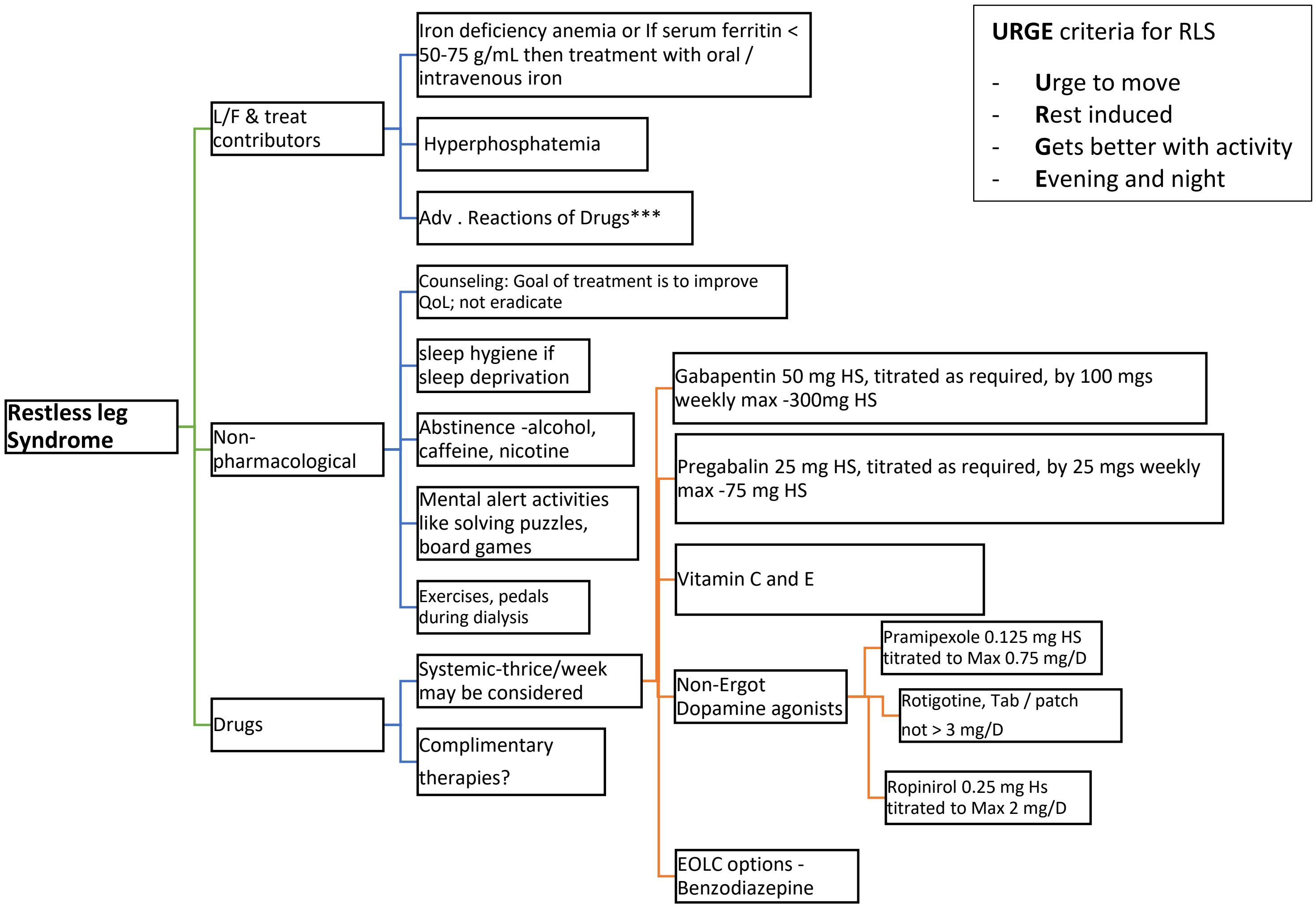

***Dopamine antagonists – Haloperidol, metoclopramide, respiridone, quetiapine, olanzepine, anti depressants – SSRIs, SNRIs, TCAs, opioids, Ca blockers, Carbamazepine, Lithium

Restless Leg Hand-out-Ref: https://www.thoracic.org/patients/patient-resources/resources/rls.pdf; https://my.clevelandclinic.org/ccf/media/files/Sleep_Disorders_Center/Restless_Legs.pdf

- Life style
    - Avoid substances that can trigger symptoms, such as alcohol, tobacco. Limit caffeine intake
    - Show all the drugs that you take, to the doctor.
    - Moderate aerobic exercise during the day can also help.
    - Sleep Hygiene (vide infra)
    - If you need to travel for long periods of time, consider traveling at a time when your symptoms are least severe (usually in the morning). Try to plan for breaks or periods of time when you can walk around and stretch
- Activities that can reduce symptoms
    - walking, stretching, massaging the affected area
    - taking a warm or cool bath, applying hot or cold packs, soaking in a warm bath can be helpful
    - relaxation techniques
    - mentally distracting activity, such as crossword puzzles, crochet, or talking to someone

Nausea /Vomiting

- **Nausea / Vomiting**
  - L/F & treat contributors
    - Control Uraemia, dyselectrolemia
    - Adv . Reactions of Drugs- opioids, SSRIs
    - Delayed Gastric Emptying, due to uraemia or diabetic autonomic peripheral neuropathy
      Constipation
  - Non-pharmacological
    - **Constipation hand-out**:@@@
    - **Nausea /Vomiting Hand-out** +++
    - Relaxation, imagery, Acupressure
  - Drugs
    - Stable disease, mild/moderate N/V
      - 1. Metoclopramide 2.5 mg P.O or S/C q4H if gastric paresis suspected ***
        2. Cyclizine 25-50 mg upto TDS; Levomepromazine 12.5 to 25 mg q 8hr
        3. Ondansetron 4 mg TDS upto 8 mg TDs (avoid if constipated)
      - If predominant Nausea
        1. Haloperidol 0.5 mg q 12H to upto 2 mg q4H ***
        2. Olanzepine 2.5 mg q8H upto q4H ***
    - Deteriorating, persisting, pre-terminal N/V and previous options ineffective
      - Haloperidol 1 mg S/C q8H & PRN, upto Max 5 mg /24 H
      - Methotrimeprazine 6.25 mg S/C (5 mg P.O)
    - N/V with RLS -Avoid Metoclopramide/Halperidol and use Motilium 10mg half hour before meals
    - Complimentary therapies - Ginger

***L/F Extra pyramidal Symptoms; can worsen Restless leg syndrome

Nausea Vomiting Hand-out

1. Oral hygiene
2. Small volume, frequent meals, chewed slowly
3. No Alcohol
4. Advice to drink water before / after, but not with meals
5. Minimize aromas
6. Use ginger
7. Use Loose fitting clothes
8. Avoid spicy, greasy, excessive sweet foods, patient choice
9. Relax in up-right position after meals, to facilitate gastric phase of the digestion
10. Apply cool damp cloth on forehead/nape of the neck

Nociceptive Pain

Ref: Safe and Effective Management of Pain in People with CKD. Sara N. Davison et al; CJASN 14: 1551–1553, 2019

The risks of NSAIDs: progression of CKD, GI bleeding, and cardiovascular morbidity and mortality
Use only if most essential, & only in anuric patients

Agree on the goal of therapy- to relieve the pain to allow function and QOL

Nociceptive Pain

- Step 1 - Mild Pain NRS<4 /10
  - Paracetamol 500 mg q8H upto 3Gms/D. Adjuvants when indicated
  - Consider Topical preparations, / topical NSAIDs- Diclofenac 5% or 10% gel
  - If anuric, without APD /cardiovascular diseases - NSAIDs in short bursts ??
- Step 2: weak opioids - if Pain persisting, increasing despite Step 1
  - Step 1 + Tapentadol Not > 100mg/D or cautious use of Tramadol 50 mg -1/2 PNR upto & not > 100 mg/D (administered post dialysis). Use 37.5 mg if GFR < 30 ml/Min
- Step 3: Strong opioids - If Pain persisting, increasing despite Step 2
  - The dose of the Fentanyl patch after 24 Hours dose determination using a S/C syringe pump titrated by monitoring pain. Patches - 12.5 ug/H; 25ug/H; 50ug/H; 75ug/H; 100ug/H
    - delay of 6-10 hours after application; Avoid hairy skin surface, direct heat, fever
  - Oral Morphine - start very low, cautious use - 2.5 mg q12H upto 10 mg q8H. Titrate up not earlier than every 3 days. Avoid in stage 5 CKD
  - Cautious monitored titration of Methadone (renal safe; unpredictable cumulation) - 1 to 2 mg /D P.O titrate to 2.5 mg q12H . Special training required to use Methadone
  - Hydromorphone (currently unavailable in India)
  - Buprenorphine patch 5ug q7Days; S/C injection 0.3 totrated to 0.6mg/ D
  - Opioid Risk assessment
    - H/O Smoking, alcohol, drug abuse; psychological morpbifities; family History of adictoons/psychological morbidities
  - L/F and manage opioid induced N/V with Metoclopramide or Haloperidol (Haloperidol 1 mg S/C q8H & PRN, upto Max 5 mg /24 H). Breakthrough dose = 10% of 24 H dose
  - Bowel regimen. Treat opioid induced constipation with stimulant laxatives
- Complimentary therapuies Relaxation, imagery, Acupressure
- Monitor 2 weekly - 4 As - Analgesia, Activity level, adverse effects, addiction potential

Non-Pharmacological therapy:

- Exercise, physiotherapy, Stretching, and massage;
- -behavioral therapies – CBT, relaxation techniques
- L/F and treat coexisting depression, anxiety, and sleep disorders

Bone Pain

Agree on the goal of therapy- to relieve the pain to allow function and QOL

Bone related Pain

- Step 1 - Mild Pain
  NRS<4 /10
  - Paracetamol 500 mg q8H upto 3Gms/D. Adjuvants when indicated
  - If anuric, without APD /cardiovascular diseases - NSAIDs in short bursts ??
- Step 2: weak opioids - if Pain persisting, increasing despite Step 1
  - Step 1 + Tapentadol Not > 100mg/D or cautious use of Tramadol 50 mg -1/2 PNR upto & not > 100 mg/D
- Step 3: Strong opioids - If Pain persisting, increasing despite Step 2
  - The dose of the Fentanyl patch after 24 Hours dose determination using a S/C syringe pump with titration based on pain. Available as 12.5 ug/H; 25ug/H; 50ug/H; 75ug/H; 100ug/H
    - delay of 6-10 hours after application; Avoid hairy skin surface, direct heat, fever
  - Oral Morphine - reduced frequency, low dose cautious use - 2.5 mg q12H upto 10 mg q8H. Titrate up not earlier than every 3 days.
  - Cautious monitored titration of Methadone (renal safe; unpredictable cumulation) - 1 to 2 mg /D P.O titrate to 2.5 mg q12H . Special training required to use Methadone
  - Hydromorphone (currently unavailable in India)
  - Buprenorphine patch 5ug q7Days
  - Opioid Risk assessment
    - H/O Smoking, alcohol, drug abuse; psychological morpbifities; family History of adictoons/psychological morbidities
  - L/F and manage opioid induced N/V with Metoclopramide or Haloperidol (Haloperidol 1 mg S/C q8H & PRN, upto Max 5 mg /24 H). Breakthrough dose = 10% of 24 H dose
  - Bowel regimen
- Complimentary therapuies
  Relaxation, imagery, Acupressure
- Monitor 2 weekly - 4 As - Analgesia, Activity level, adverse effects, addiction potential

Neuropathic Pain

Neuropathic Pain

- 1st line
  - Gabapentin 50 mg HS, titrated as required, by 100 mgs weekly max -300mg HS
  - Pregabalin 25 mg HS, titrated as required, by 25 mgs weekly max -75 mg HS
- 2nd line
  - TCA 10-25 mg/D titrate to maximum 75 mg /D (caution: conduction anomolies)
  - Paracetamol 500 mg q8H upto 3Gms/D and / OR weak opioid
- Step 3: Strong opioids - If Pain persisting, increasing despite 1st and 2nd line treatment
  Neuropathic pain is only partially responsive to opioids
  - The dose of the Fentanyl patch after 24 Hours dose determination using a S/C syringe pump with titration based on pain. Available as 12.5 ug/H; 25ug/H; 50ug/H; 75ug/H; 100ug/H
    - delay of 6-10 hours after application; Avoid hairy skin surface, direct heat, fever
  - Oral Morphine - reduced frequency, low dose cautious use - 2.5 mg q12H upto 10 mg q8H. Titrate up not earlier than every 3 days.
  - Cautious monitored titration of Methadone (renal safe; unpredictable cumulation) - 1 to 2 mg /D P.O titrate to 2.5 mg q12H . Special training required to use Methadone
  - Hydromorphone (currently unavailable in India)
  - Buprenorphine patch 5ug q7Days
  - Opioid Risk assessment
    - H/O Smoking, alcohol, drug abuse; psychological morpbifities; family History of adictoons/psychological morbidities
  - L/F and manage opioid induced N/V with Metoclopramide or Haloperidol (Haloperidol 1 mg S/C q8H & PRN, upto Max 5 mg /24 H) Breakthrough dose = 10% of 24 H dose
  - Bowel regimen
- Complimentary therapuies Relaxation, imagery, Acupressure
- Monitor 2 weekly - 4 As - Analgesia, Activity level, adverse effects, addiction potential (Pain assessment Tool?)

Breathlessness

- **Breathlessness**
  Treat when it affects QoL
  - L/F & treat contributors
    - Anxiety, anemia, infection
    - Respiratory secretions
      - 1st line - Hyoscine Butylbromide (Buscopan) 20 mg prn. If secretions do not resolve with repositioning and PRN Buscopan, start Buscopan 60 mg SC/24 h in a syringe driver. The dose can be titrated up to max 180 mg in 24 h as needed.
      - 2nd line: Glycopyrronium 0.2–0.4 mg s/c qds prn. May be used in a syringe driver: 1.2–2.4 mg/24 h
      - Hyoscine hydrobromide - Not recommended - crooses BBB, excitatory /sedative effect.
  - Non-pharmacological
    - **Restrict water intake = urine volume + 600 ml**
    - Propped up Position, Abdominal breathing, pursed lip breathing, exercise to capacity
    - When distressed with breathlessness - Open windows, Hand-held Fan, Shoulder/ back massage, Relaxation
    - Oxygen only if it helps relieve the distress
  - Complimentary therapies
  - Drugs
    - If Pulmonary edema
      - 1. Furosemide for pulmonary edema (balance with dehydration, hypotension)
        2. Metolazone 2.5 mg to 5 mg P.O + Furosamide upto 120 mg P.O. BD for 3-5 days
        3. SCUF (slow continuous ultrafiltration) to alleviate symptoms - ethically complex
      - Fentanyl 12.5 ug SC/SL / Intra-nasal (? OTFC)
        Morphine ? dose - 1 mg SC
      - ? Negotiation - dialysis if functions > 50%
    - If Anemia - erythropoietin, Fe^{++}
    - Deteriorating, persisting, pre-terminal breathlessness, and previous options ineffective
      - Opioids - very low doses titrted to effect
        lorazepam 0.5 to 1.0mg OR Midazolam 1.25 -2-.5 mg 6-8 hourly
        S/C infusion - Morphine sulphate 5-10 mg SC/24 h. with Midazolam 10 mg SC/24 h if distressed.

Breathlessness Hand-out

Breathlessness – Non-pharmacological therapies

1. Explore triggers and alleviators
2. Help experience awareness when symptom is under control
3. Educate belly breathing, pursed lip breathing
4. Music, meditation, mindfulness, Relaxation techniques
5. Diet review
6. During an attack
    a. Open windows, open space around patient, loosen clothes
    b. Reassurance, calmness in the responses
    c. hand-held Fan, ceiling fan – Trigeminal V 2 stimulation
    d. Oxygen – if it helps relieve symptom
    e. shoulder-back massage

Sleeplessness

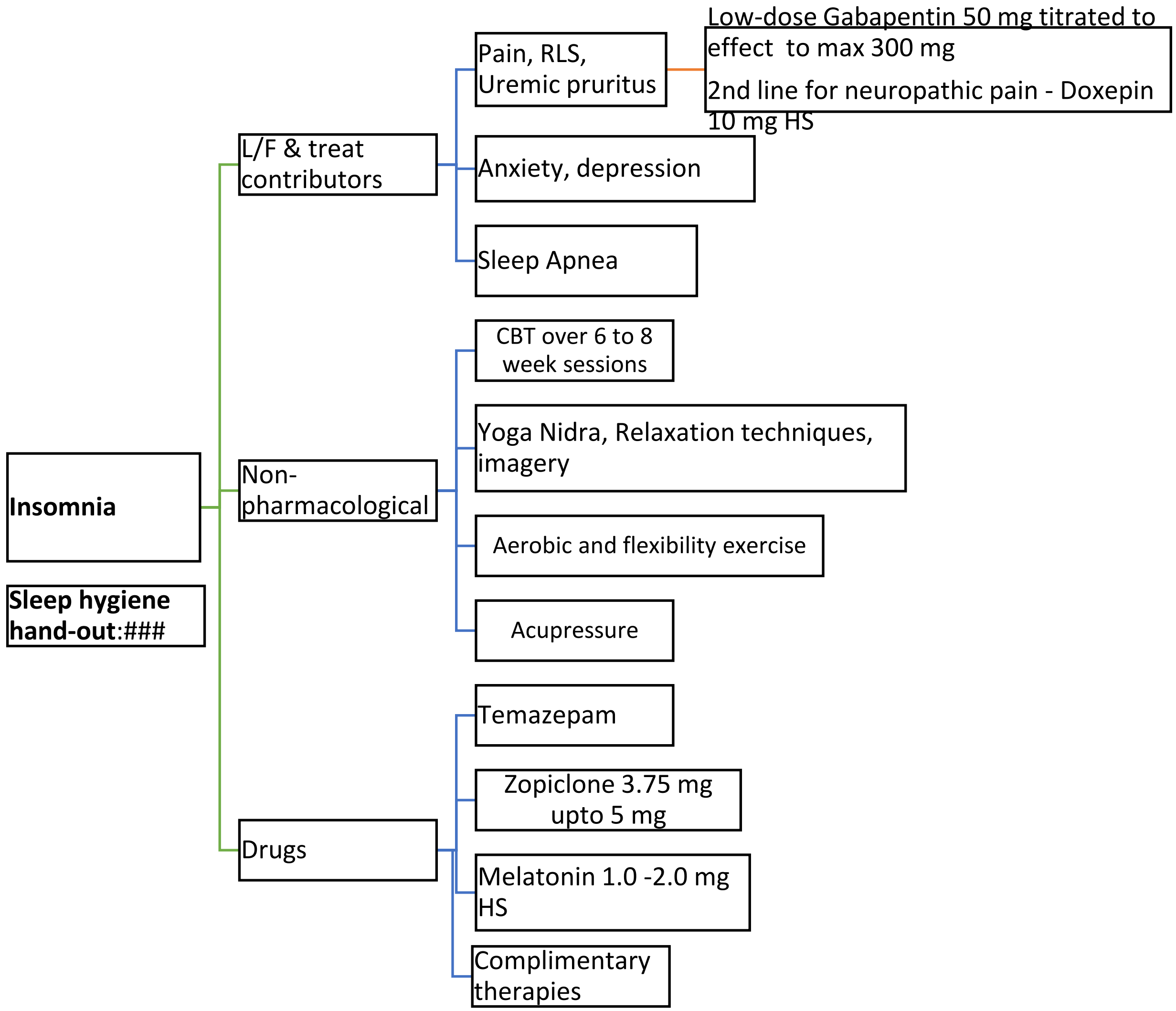

Sleep Hygiene Handout: Ref: www.cci.health.wa.gov.au ;

- **Get regular**. Train your body to sleep well by going to bed and getting up at more or less the same time every day, no matter how well or how poorly you have slept- even on weekends and days off!
- **Get Some Natural Light**. Try to spend some time outdoors or in natural light every day. Getting some sunlight early in the day can be helpful for setting your body's natural wake and sleep cycle.
- **Sleep when sleepy**. Only try to sleep when you actually feel tired or sleepy, rather than spending too much time awake in bed
- **Get out of bed when not sleeping & try again**. If you haven't been able to get to sleep after about 20 minutes or more, get up and do something calming or boring until you feel sleepy, then return to bed and try again. Sit quietly on the couch with the lights off (bright light will tell your brain that it is time to wake up), or read something boring like the phone book. Avoid doing anything that is too stimulating or interesting, as this will wake you up even more.
- **Avoid caffeine & nicotine**. It is best to avoid consuming any caffeine (in coffee, tea, cola drinks, chocolate, and some medications) or nicotine (cigarettes) for at least 4-6 hours before going to bed. These substances act as stimulants and interfere with the ability to fall asleep
- **Avoid alcohol**. It is also best to avoid alcohol for at least 4-6 hours before going to bed. Many people believe that alcohol is relaxing and helps them to get to sleep at first, but it actually interrupts the quality of sleep.
- **Use Bed for sleeping**. Try not to use your bed for anything other than sleeping and sex, so that your body comes to associate bed with sleep. If you use bed as a place to watch TV, eat, read, work on your laptop, pay bills, and other things, your body will not learn this connection.
- **No napping during the day**. This makes sure that you are tired at bedtime. If you can't make it through the day without a nap, make sure it is for less than an hour and before 3pm.
- **Sleep rituals / routines**. You can develop your own rituals of things to remind your body that it is time to sleep. establish a set routine that you follow every night. For example, have a hot bath, put on your pajamas, brush your teeth, and then listen to soft music and read on the couch until you start to feel sleepy and then go to bed. Some people find it useful to do relaxing stretches or breathing exercises for 15 minutes before bed each night, or sit calmly with a cup of caffeine-free tea.
- **Relax**. Try doing something to relax your body and mind before going to bed. Try taking a hot bath 90 minutes before you plan to go to bed. Or, try a relaxation exercise (see Calm Breathing and Progressive Muscle Relaxation), meditation, or listening to calming music.
- **Bathing**. Having a hot bath 1-2 hours before bedtime can be useful, as it will raise your body temperature, causing you to feel sleepy as your body temperature drops again.
- **No clock-watching**. Frequently checking the clock during the night can wake you up (especially if you turn on the light to read the time) and reinforces negative thoughts such as "Oh no, look how late it is, I'll never get to sleep" or "it's so early, I have only slept for 5 hours, this is terrible."
- **Don't Worry.** Leave your worries about work, school, health, relationships, etc. out of the bedroom. Try scheduling a "worry time" earlier in the evening to deal with your worries. Write a to-do list for the next day/week. Maintain a journal & jot down the events, thoughts of the

day, plans for next day is helpful. If you wake up in the middle of the night worrying, try writing down your worries and tell yourself that you will address them in the morning.

- **Exercise during the day**. Regular exercise is a good idea to help with good sleep, but try not to do strenuous exercise in the 4 hours before bedtime. Morning walks are a great way to start the day feeling refreshed!
- **Eat right**. A healthy, balanced diet will help you to sleep well, but timing is important. Some people find that a very empty stomach at bedtime is distracting, so it can be useful to have a light snack, but a heavy meal soon before bed can also interrupt sleep. Some people recommend a warm glass of milk, which contains tryptophan, which acts as a natural sleep inducer.
- **The right space**. It is very important that your bed and bedroom are quiet and comfortable for sleeping. A cooler room with enough blankets to stay warm is best, and make sure you have curtains or an eyemask to block out early morning light and earplugs if there is noise outside your room. Make sure that you have a supportive mattress and fresh, comfortable bedding. Also, try to ensure that your room is not too hot or cold, minimize noise, and block out light.
- **Keep daytime routine the same**. Even if you have a bad night sleep and are tired it is important that you try to keep your daytime activities the same as you had planned. That is, don't avoid activities because you feel tired. This can reinforce the insomnia.
- **Start Small**! Making small changes can have a large impact on your sleep. Don't try to do everything all at once. Instead, pick one or two strategies and try them consistently. When you're ready, try adding a new strategy. The goal is to slowly start increasing behaviours that can help you sleep, while reducing the things that are interfering with your sleep.
- **Be Consistent**. Pick a strategy and use it consistently. Try to do the same thing every night.
- **Be Patient**. These strategies can take time to improve your sleep. In fact, sometimes things can get worse before they get better. Hang in there and stick with it!
- **Chart Your Progress**. Use the Sleep Diary form to keep track of the strategies you're using and your weekly progress.

Fatigue

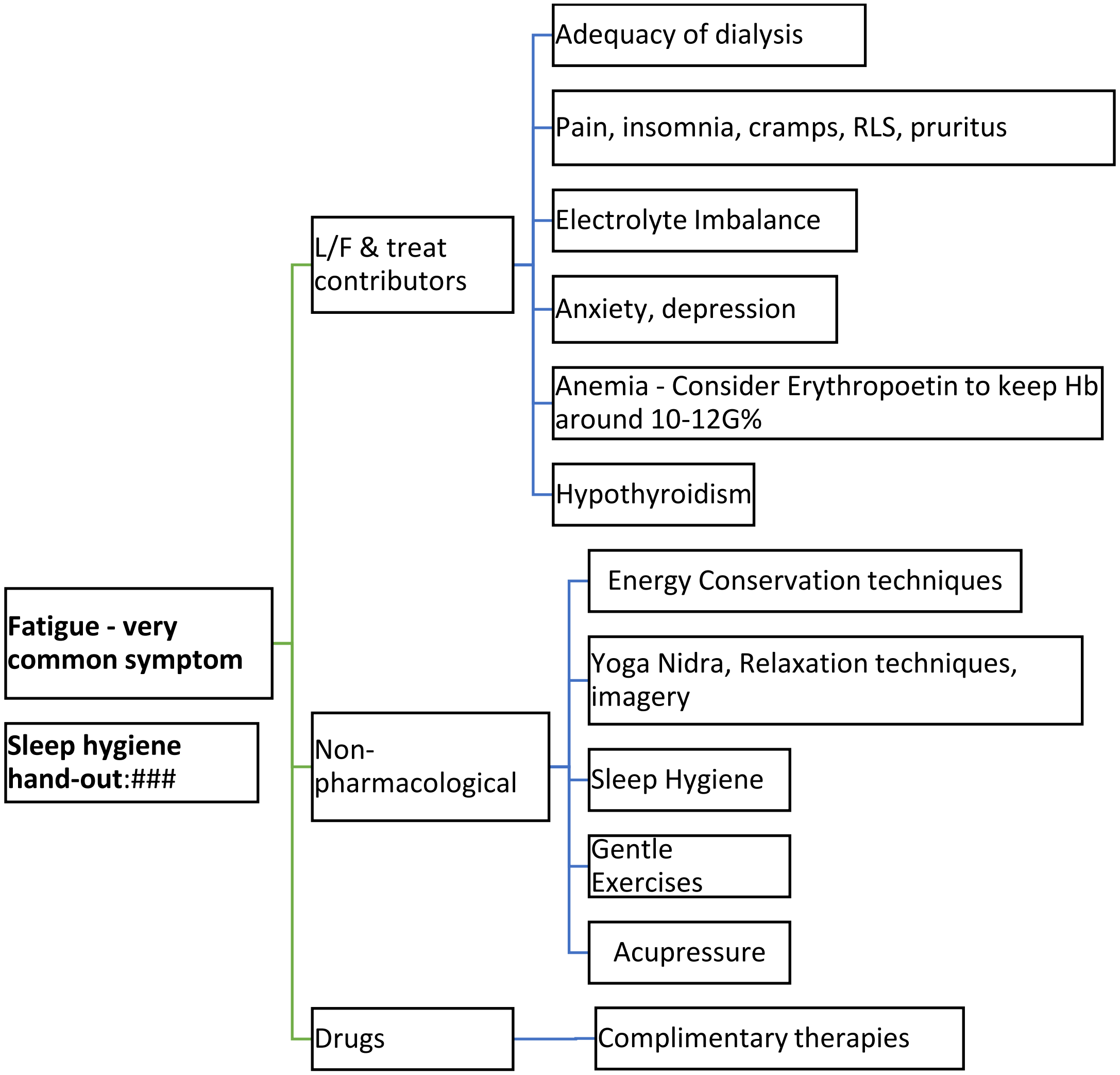

Fatigue - very common symptom
Sleep hygiene hand-out:###
L/F & treat contributors
Adequacy of dialysis
Pain, insomnia, cramps, RLS, pruritus
Electrolyte Imbalance
Anxiety, depression
Anemia - Consider Erythropoetin to keep Hb around 10-12G%
Hypothyroidism
Non-pharmacological
Energy Conservation techniques
Yoga Nidra, Relaxation techniques, imagery
Sleep Hygiene
Gentle Exercises
Acupressure
Drugs
Complimentary therapies

Nutritional concerns

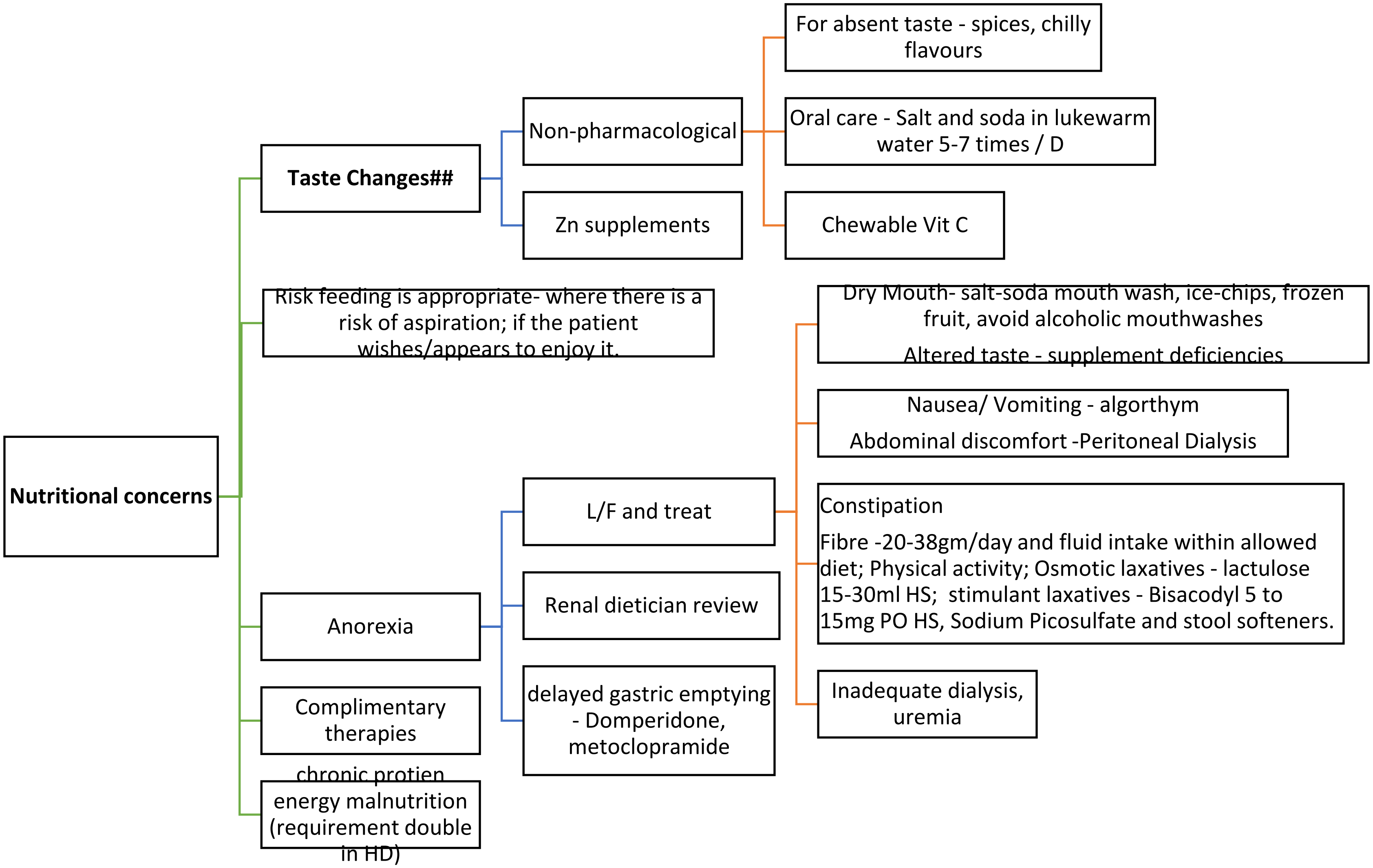

Urea→ bitter; Sodium→salty; Potassium→metallic; reduced Zn→ absence of taste

Generalised Edema

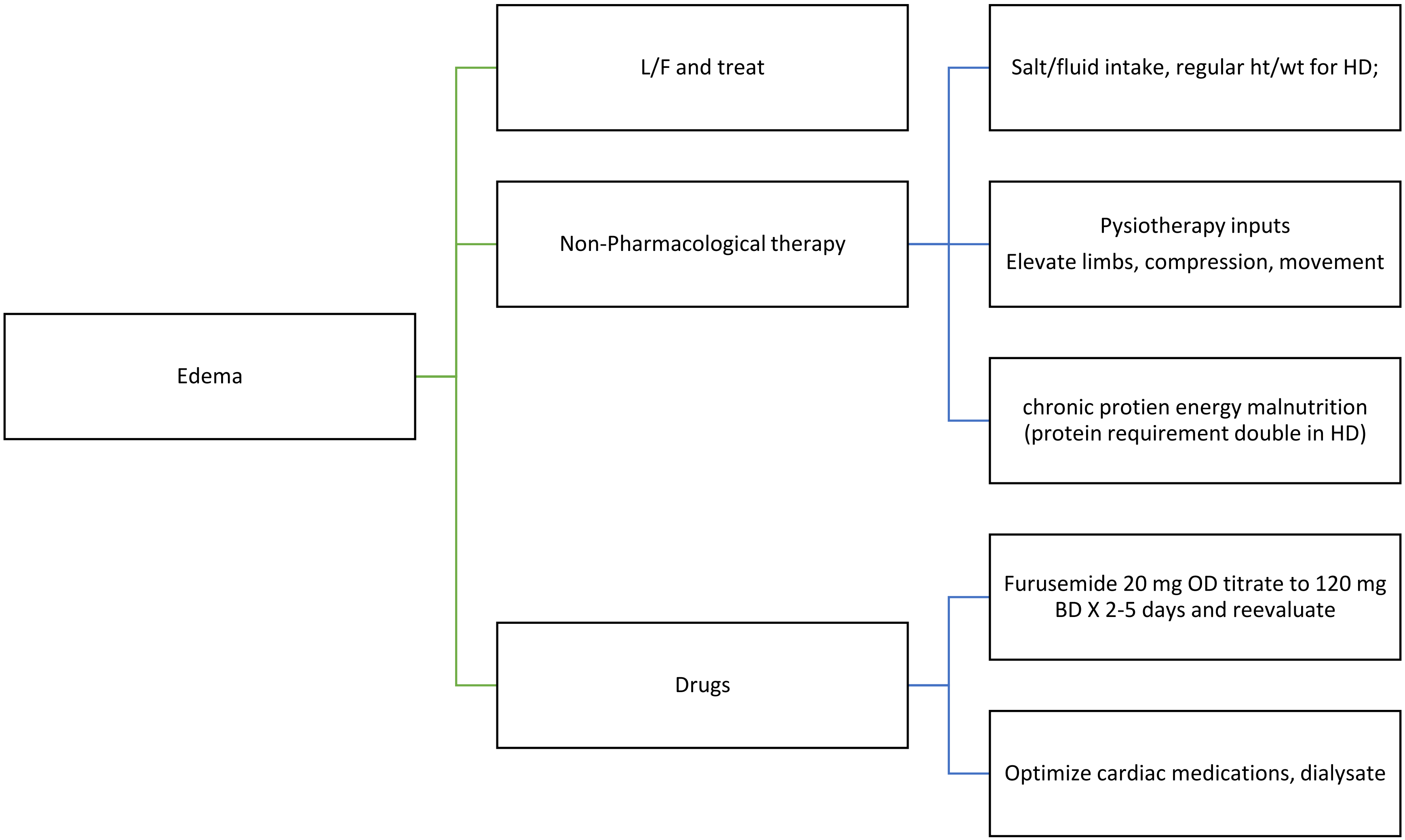

Depression

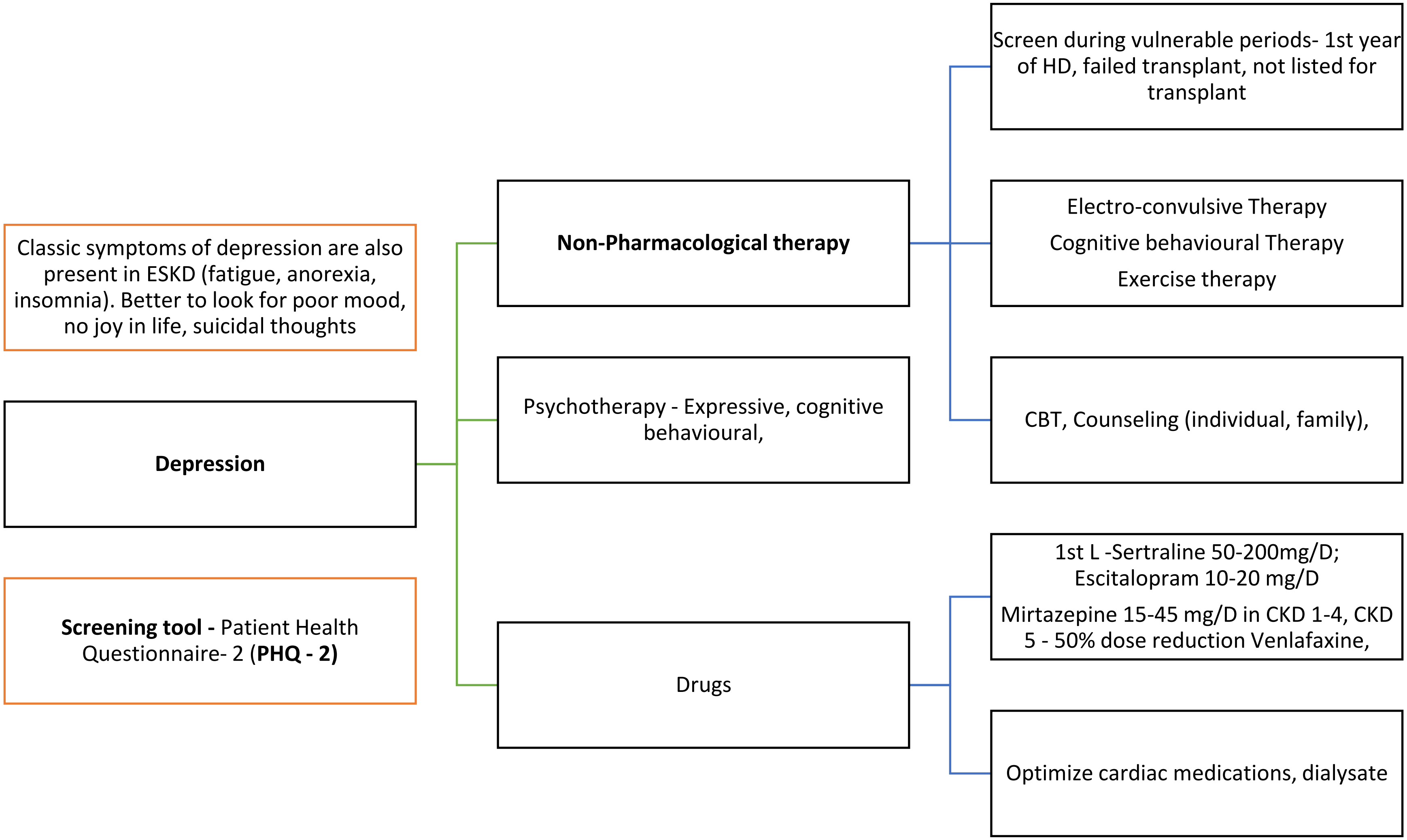
Classic symptoms of depression are also present in ESKD (fatigue, anorexia, insomnia). Better to look for poor mood, no joy in life, suicidal thoughts
Depression
Screening tool - Patient Health Questionnaire- 2 (PHQ - 2)
Non-Pharmacological therapy
Psychotherapy - Expressive, cognitive behavioural,
Drugs
Screen during vulnerable periods- 1st year of HD, failed transplant, not listed for transplant
Electro-convulsive Therapy
Cognitive behavioural Therapy
Exercise therapy
CBT, Counseling (individual, family),
1st L -Sertraline 50-200mg/D; Escitalopram 10-20 mg/D
Mirtazepine 15-45 mg/D in CKD 1-4, CKD 5 - 50% dose reduction Venlafaxine,
Optimize cardiac medications, dialysate

Supplementary 7

Interview guide for advance care planning discussion in ESKD setting (English)

Sometimes, it is difficult to make healthcare-related choices, especially in situations of crisis or worsening of health. Most of us have no idea what medical decisions it would be wise to discuss with our loved ones and plan ahead for.

This conversation is to help you think through the things that matter most to you in your life so that if you ever become seriously unwell, the people looking after you will understand what's important to you.

It may be helpful to think about what is most important to you and plan for future healthcare. This is similar to how we plan for other situations in life and prepare for any other life crises.

We are all different, and we may have different choices and healthcare preferences. So there is no right or wrong answer.

You may want to consider thinking about it and revising your choices over time as life situations change.

Would you want to talk about planning for a situation or health crisis if you become seriously unwell and you are not able to communicate your choices? For example, stroke, brain damage, cardiac problems, etc.?

Yes/ No.

If yes, to above then next question

Would you be okay talking about situations that might be so serious that there is a possibility of death?

Yes/ No.

If yes, to above then next question

Hospital/ home

If you developed such a serious illness, where would you want to be looked after?

a) I would prefer to go to hospital, whether or not they can save my life.
b) Being with my loved ones is the most important thing to me. I would prefer to stay at home, whether or not I become too sick to survive.
c) If I am struggling with symptoms, like pain/ breathing difficulties/ restlessness, I would prefer care to be provided in hospital, whether or not I become too sick to survive.
d) If I am struggling with symptoms, like pain/ breathing difficulties/ restlessness, I would prefer care to be provided in home, whether or not I become too sick to survive.

e) I'm not sure yet

ICU care

In situations of serious illness, would you want to be shifted to ICU settings, even if your doctor's feel that it may potentially not be beneficial or may not improve your quality of life or length of life?

a) Yes, I would want my doctors to shift me to ICU even if that is not very beneficial.
b) No, I don't want to be admitted in ICU even if that means my condition worsens.
c) I am not sure yet.

Ventilator

In situations of serious illness, would you want to be shifted to ventilator support, even if your doctor's feel that it may potentially not be beneficial or may not improve your quality of life or length of life?

a) I would accept the ventilator, in the hope of saving my life and accepting the risk that it may leave me with life-long after effects.
b) I would prefer not to have a ventilator, even if this means I don't survive. I want my doctors to treat my symptoms of discomfort.
c) I'm not sure yet

Cardiopulmonary resuscitation

In situations of serious illness, would you want to receive cardio pulmonary support, even if your doctor's feel that it may potentially not be beneficial or may not improve your quality of life or length of life?

a. I would accept CPR.
b. I would not like CPR to be attempted, even though I would die.
c. I'm not sure yet.

Dialysis

In situations of serious illness, would you want to continue dialysis?

a. Yes
b. No
c. Not sure yet.

Living longer or living comfortably

When considering treatment decisions that may impact my length of survival and my comfort, I would prefer to focus on -

a. living longer
b. living comfortably

c. Not sure yet

Surrogate decision maker

If you are not able to voice your choices or make your medical treatment related decisions, who according to you should be making medical decisions on your behalf?

a. My family.... Specify

b. My doctors

c. My family, after discussing with my doctors

d. Any others.... Specify

Thank you for your time. We have recorded your choices. You may want to discuss about your plans and choices with your family and / or loved ones and revisit these choices any number of times. Please feel free to discuss this with your doctors or other dialysis team members.

Would you wish to formally document your choices/ preferences, once you are sure about the above answers?

a. Yes
b. No

Interview guide for advance care planning discussion in ESKD setting (Kannada)

ಶಾಶ್ವತ ಮೂತ್ರಪಿಂಡ ರೋಗಿಗಳಿಗೆ ಮುಂಗಡ ಅರೋಗ್ಯ ಯೋಜನೆಯ ಚರ್ಚೆಗಾಗಿ ಸಂದರ್ಶನ ಮಾರ್ಗದರ್ಶಿ

ಕೆಲವೊಮ್ಮೆ ಆರೋಗ್ಯಕ್ಕೆ ಸಂಬಂಧಿಸಿದ ಆಯ್ಕೆಗಳನ್ನು ಮಾಡುವುದು ಕಷ್ಟಕರವಾಗಿರುತ್ತದೆ, ವಿಶೇಷವಾಗಿ ಬಿಕ್ಕಟ್ಟಿನ ಸಂದರ್ಭಗಳಲ್ಲಿ ಅಥವಾ ಆರೋಗ್ಯವು ಹದಗೆಟ್ಟಾಗ, ನಮ್ಮ ಪ್ರೀತಿಪಾತ್ರರ ಜೊತೆ ಚರ್ಚೆ ಮಾಡಿ ಮುಂದೆ ಯಾವ ವೈದ್ಯಕೀಯ ನಿರ್ಧಾರಗಳನ್ನು ತೆಗೆದುಕೊಳ್ಳಬೇಕು ಎಂದು ಬಗ್ಗೆ ನಮಗೆ ಹೆಚ್ಚಿನವರಿಗೆ ತಿಳಿದಿರುವುದಿಲ್ಲ.
ಈ ಸಂಭಾಷಣೆಯು ನಿಮ್ಮ ಜೀವನದಲ್ಲಿ ನಿಮಗೆ ಹೆಚ್ಚು ಮುಖ್ಯವಾಗಿದೆ. ಆರೋಗ್ಯ ಹದಗೆಟ್ಟಾಗ ಏನು ಮಾಡಬೇಕು ಎಂಬ ವಿಷಯಗಳ ಬಗ್ಗೆ ಯೋಚಿಸಲು ಸಹಾಯ ಮಾಡುತ್ತದೆ. ಇದರಿಂದ ನೀವು ಎಂದಾದರೂ ಗಂಭೀರವಾಗಿ ಅಸ್ವಸ್ಥರಾಗಿದ್ದರೆ, ನಿಮ್ಮನ್ನು ನೋಡಿಕೊಳ್ಳುವ ನಿಮ್ಮ ಮನೆಯವರಿಗೆ ನಿಮಗೆ ಮುಖ್ಯವಾಗಿ ಯಾವ ರೀತಿಯ ಚಿಕಿತ್ಸೆ ಇಷ್ಟವಿದೆ ಎಂಬುದರ ಬಗ್ಗೆ ಹೆಚ್ಚಿನ ಅರಿವು ನೀಡಲು ಸಹಾಯವಾಗುತ್ತದೆ.

ನಿಮಗೆ ಆರೋಗ್ಯ ಹದಗೆಟ್ಟಾಗ, ಯಾವ ರೀತಿಯ ಚಿಕಿತ್ಸೆ ಪ್ರಮುಖವಾದದ್ದು ಹಾಗೂ ಅದನ್ನು ಮುಂಚಿತವಾಗಿ ಯೋಜಿಸಲು ಈ ಸಂಭಾಷಣೆ ಸಹಾಯ ಮಾಡುತ್ತದೆ. ಇದು ನಾವು ಜೀವನದ ಇತರ ಸನ್ನಿವೇಶಗಳಿಗೆ ಹೇಗೆ ಯೋಚಿಸುತ್ತೇವೆ ಮತ್ತು ಯಾವುದೇ ಇತರ ಜೀವನದ ಕಷ್ಟಗಳಿಗೆ ಹೇಗೆ ತಯಾರಾಗಿರುತ್ತೇವೆ ಅದೇ ರೀತಿ ಇದು ಕೂಡ.
ನಾವೆಲ್ಲರೂ ವಿಭಿನ್ನವಾಗಿದ್ದೇವೆ ಮತ್ತು ನಮಗೆ ಬೇರೆ ಬೇರೆ ಆಯ್ಕೆಗಳು ಮತ್ತು ಆರೋಗ್ಯ ಆದ್ಯತೆಗಳು ಇರಬಹುದು. ಆದ್ದರಿಂದ ಇದೆ ಸರಿ ಅಥವಾ ತಪ್ಪು ಅಂತ ಹೇಳಲು ಸಾಧ್ಯವಿಲ್ಲ. ನಿಮಗೆ ಸೂಕ್ತವಾದ ಆಯ್ಕೆ ಮಾಡಬಹುದು.
ಜೀವನದ ಸನ್ನಿವೇಶಗಳು ಬದಲಾದಂತೆ ನಿಮ್ಮ ಆಯ್ಕೆಗಳು ಸ್ವಲ್ಪ ಕಾಲಾನಂತರದಲ್ಲಿ ಪರಿಷ್ಕರಿಸಲು ಅಥವಾ ಬದಲಾಯಿಸಲು ನೀವು ಬಯಸಬಹುದು. ಮತ್ತು ಅದನ್ನು ಪರಿಷ್ಕರಿಸಬಹುದು.

ನೀವು ಗಂಭೀರವಾಗಿ ಅಸ್ವಸ್ಥರಾಗಿದ್ದರೆ ಮತ್ತು ನಿಮ್ಮ ಆಯ್ಕೆಗಳನ್ನು ತಿಳಿಸಲು ಸಾಧ್ಯವಾಗದಿರುವ ಪರಿಸ್ಥಿತಿಯಲ್ಲಿದ್ದರೆ, ಅಂದರೆ ಪಾರ್ಶ್ವವಾಯು, ಮಿದುಳಿನ ಹಾನಿ, ಹೃದಯ ಸಮಸ್ಯೆಗಳು, ಇತ್ಯಾದಿ ಆ ಸಂಧರ್ಭದ ಬಗ್ಗೆಗಿನ ಆರೋಗ್ಯದ ಯೋಜನೆಯ ಬಗ್ಗೆ ಮಾತನಾಡಲು ನೀವೀಗ ಬಯಸುತ್ತೀರಾ.
ಹೌದು / ಇಲ್ಲ.
ಹೌದಾದರೆ, ...ಮುಂದಿನ ಪ್ರಶ್ನೆ

ಸಾವಿನ ಸಾಧ್ಯತೆ ಇರುವಷ್ಟು ಗಂಭೀರವಾದ ಸನ್ನಿವೇಶಗಳ ಬಗ್ಗೆ ನಿಮ್ಮ ಹತ್ತಿರ ಮಾತನಾಡಬಹುದೇ?
ಹೌದು/ ಇಲ್ಲ.
ಹೌದಾದರೆ, ...ಮುಂದಿನ ಪ್ರಶ್ನೆ

ಆಸ್ಪತ್ರೆ/ಮನೆ
ಒಂದು ವೇಳೆ ನೀವು ಅಂತಹ ಗಂಭೀರ ಕಾಯಿಲೆಯನ್ನು ಹೊಂದಿದ್ದರೆ, ನಿಮ್ಮನ್ನು ಎಲ್ಲಿ ನೋಡಿಕೊಳ್ಳಬೇಕು?
ಎ. ನಾನು ಆಸ್ಪತ್ರೆಗೆ ಹೋಗಲು ಬಯಸುತ್ತೇನೆ, ಅಲ್ಲಿ ಅವರು ನನ್ನ ಜೀವವನ್ನು ಉಳಿಸಬಹುದು ಅಥವಾ ಉಳಿಸಲು ಸಾಧ್ಯವಾಗದಿರಬಹುದು, ಆದರೂ ಆಸ್ಪತ್ರೆಗೆ ಹೋಗಲು ಬಯಸುತ್ತೇನೆ.
ಬಿ. ನನ್ನ ಪ್ರೀತಿಪಾತ್ರರ ಜೊತೆ ಇರುವುದು ನನಗೆ ಅತ್ಯಂತ ಮುಖ್ಯವಾದ ವಿಷಯ. ನಾನು ತುಂಬಾ ಅಸ್ವಸ್ಥರಾಗಿರಲಿ ಅಥವಾ ಇಲ್ಲದಿರಲಿ, ಮನೆಯಲ್ಲಿಯೇ ಇರಲು ನಾನು ಬಯಸುತ್ತೇನೆ.
ಸಿ. ನನಗೆ ನೋವು/ಉಸಿರಾಟದ ತೊಂದರೆಗಳು/ಚಡಪಡಿಕೆಯಂತಹ ರೋಗಲಕ್ಷಣಗಳು ಬಂದರೆ, ತುಂಬಾ ಅಸ್ವಸ್ಥನಾಗಿರಲಿ ಅಥವಾ ಇಲ್ಲದಿರಲಿ, ಆಸ್ಪತ್ರೆಯಲ್ಲಿ ಆರೈಕೆಯನ್ನು ಪಡೆದುಕೊಳ್ಳಲು ನಾನು ಬಯಸುತ್ತೇನೆ.
ಡಿ. ನನಗೆ ನೋವು/ಉಸಿರಾಟದ ತೊಂದರೆಗಳು/ಚಡಪಡಿಕೆಯಂತಹ ರೋಗಲಕ್ಷಣಗಳು ಬಂದರೆ ನಾನು ತುಂಬಾ ಅಸ್ವಸ್ಥನಾಗಿರಲಿ ಅಥವಾ ಇಲ್ಲದಿರಲಿ, ಮನೆಯಲ್ಲಿಯೇ ಆರೈಕೆಯನ್ನು ಪಡೆದುಕೊಳ್ಳಲು ನಾನು ಬಯಸುತ್ತೇನೆ.
ಇ. ನನಗೆ ಇನ್ನೂ ಖಚಿತವಿಲ್ಲ

ಐಸಿಯು ಆರೈಕೆ
ಗಂಭೀರವಾದ ಅನಾರೋಗ್ಯದ ಸಂದರ್ಭದಲ್ಲಿ ನಿಮ್ಮ ವೈದ್ಯರ ಪ್ರಕಾರ ಐಸಿಯುಗೆ ಸ್ಥಳಾಂತರಿಸುವುದರಿಂದ ಯಾವುದೇ ರೀತಿಯ ಜೀವನದ ಗುಣಮಟ್ಟ ಅಥವಾ ಜೀವಿತಾವಧಿಯಲ್ಲಿ ಸುಧಾರಣೆ ಆಗುವುದಿಲ್ಲ ಎಂದು ನಿಮ್ಮ ವೈದ್ಯರು ಭಾವಿಸಿದರೂ ಸಹ ನೀವು ಐಸಿಯುಗೆ ಸ್ಥಳಂತರಿಸಲು ಬಯಸುತ್ತೀರಾ?

ಎ. ಹೌದು, ಇದು ಹೆಚ್ಚು ಪ್ರಯೋಜನಕಾರಿಯಲ್ಲದಿದ್ದರೂ ನನ್ನ ವೈದ್ಯರು ನನ್ನನ್ನು ಐಸಿಯುಗೆ ಸ್ಥಳಾಂತರಿಸಬೇಕೆಂದು ನಾನು ಬಯಸುತ್ತೇನೆ.
ಬಿ. ಇಲ್ಲ, ನನ್ನ ಸ್ಥಿತಿಯು ಹದಗೆಟ್ಟಿದ್ದರೂ ಸಹ, ನಾನು ಐಸಿಯುನಲ್ಲಿ ದಾಖಲಾಗಲು ಬಯಸುವುದಿಲ್ಲ.
ಸಿ. ನನಗೆ ಇನ್ನೂ ಖಚಿತವಿಲ್ಲ.

ವೆಂಟಿಲೇಟರ್
ಗಂಭೀರವಾದ ಅನಾರೋಗ್ಯದ ಸಂದರ್ಭದಲ್ಲಿ ನಿಮ್ಮ ವೈದ್ಯರ ಪ್ರಕಾರ ವೆಂಟಿಲೇಟರಿಗೆ ಅಳವಡಿಸುವುದರಿಂದ ಯಾವುದೇ ರೀತಿಯ ಜೀವನದ ಗುಣಮಟ್ಟ ಅಥವಾ ಜೀವಿತಾವಧಿಯಲ್ಲಿ ಸುಧಾರಣೆ ಆಗುವುದಿಲ್ಲ ಎಂದು ನಿಮ್ಮ ವೈದ್ಯರು ಭಾವಿಸಿದರೂ ಸಹ ನೀವು ವೆಂಟಿಲೇಟರಿಗೆ ಹಾಕಲು ಬಯಸುತ್ತೀರಾ?

ಎ. ನನ್ನ ಜೀವವನ್ನು ಉಳಿಸುವ ಭರವಸೆಯಲ್ಲಿ, ನಾನು ವೆಂಟಿಲೇಟರ್ ಅನ್ನು ಸ್ವೀಕರಿಸುತ್ತೇನೆ ಮತ್ತು ಜೀವಿತಾವಧಿಯಲ್ಲಿ, ಅದರಿಂದಾಗುವ ಅಪಾಯವನ್ನು ಸ್ವೀಕರಿಸಲು ಸಿದ್ಧನಾಗಿದ್ದೇನೆ.
ಬಿ. ನಾನು ಬದುಕುಳಿಯುವುದಿಲ್ಲ ಎಂದು ತಿಳಿದರೂ ಸಹ ವೆಂಟಿಲೇಟರ್ ಅನ್ನು ಸ್ವೀಕರಿಸಲು ಬಯಸುದಿಲ್ಲ. ನನ್ನ ವೈದ್ಯರು ನನ್ನ ಉಳಿದ ಅಸ್ವಸ್ಥತೆಯ ಲಕ್ಷಣಗಳಿಗೆ ಚಿಕಿತ್ಸೆ ನೀಡಬೇಕೆಂದು ನಾನು ಬಯಸುತ್ತೇನೆ.
ಸಿ. ನನಗೆ ಇನ್ನೂ ಖಚಿತವಿಲ್ಲ

ಹೃದಯ ಮತ್ತು ಶ್ವಾಸಕೋಶ ಪ್ರಚೋದಕ
ಗಂಭೀರವಾದ ಅನಾರೋಗ್ಯದ ಸಂದರ್ಭದಲ್ಲಿ, ನಿಮ್ಮ ವೈದ್ಯರ ಪ್ರಕಾರ ಸಿಪಿಆರ್ ಅನ್ನು ಪಡೆದುಕೊಳ್ಳುವುದರಿಂದ ಯಾವುದೇ ರೀತಿಯ ಜೀವನದ ಗುಣಮಟ್ಟ ಅಥವಾ ಜೀವಿತಾವಧಿಯಲ್ಲಿ ಸುಧಾರಣೆ ಆಗುವುದಿಲ್ಲ ಎಂದು ನಿಮ್ಮ ವೈದ್ಯರು ಭಾವಿಸಿದರೂ ಸಹ ನೀವು ಸಿಪಿಆರ್ ಪಡೆದುಕೊಳ್ಳಲು ಬಯಸುತ್ತೀರಾ?
ಎ. ಸಿಪಿಆರ್ ಅನ್ನು ಸ್ವೀಕರಿಸುತ್ತೇನೆ
ಬಿ. ನಾನು ಸಾಯುವ ಪರಿಸ್ಥಿತಿಯಲ್ಲಿ ಇದ್ದರೂ ಕೂಡ ಸಿಪಿಆರ್ ಅನ್ನು ನನ್ನ ಮೇಲೆ ಪ್ರಯತ್ನಿಸಲು

ಬಯಸುವುದಿಲ್ಲ.
ಸಿ. ನನಗೆ ಇನ್ನೂ ಖಚಿತವಿಲ್ಲ.

ಡಯಾಲಿಸಿಸ್
ಗಂಭೀರ ಅನಾರೋಗ್ಯದ ಸಂದರ್ಭದಲ್ಲಿ, ನೀವು ಡಯಾಲಿಸಿಸ್ ಅನ್ನು ಮುಂದುವರಿಸಲು ಬಯಸುವಿರಾ?
ಎ. ಹೌದು
ಬಿ. ಇಲ್ಲ
ಸಿ. ಇನ್ನೂ ಖಚಿತವಿಲ್ಲ.

ಹೆಚ್ಚು ಕಾಲ ಬದುಕುವುದು ಅಥವಾ ಆರಾಮವಾಗಿ ಬದುಕುವುದು
ನನ್ನ ಜೀವಿತಾವಧಿ ವಿಸ್ತರಣೆ ಅಥವಾ ಆರಾಮದಾಯಕ ಗುಣಮಟ್ಟ ಜೀವನದಲ್ಲಿ ಒಂದನ್ನು ಆಯ್ಕೆ ಮಾಡಬೇಕಾದಲ್ಲಿ ಚಿಕಿತ್ಸಾ ನಿರ್ಧಾರಗಳನ್ನು ತೆಗೆದುಕೊಳ್ಳುವಾಗ ನಾನು ಈ ಆಯ್ಕೆಗಳನ್ನು ಬಯಸುತ್ತೇನೆ.
ಎ. ಜೀವಿತಾವಧಿ ವಿಸ್ತರಣೆ ಬಯಸುತ್ತೇನೆ
ಬಿ. ಜೀವನದ ಗುಣಮಟ್ಟ ಉತ್ತಮ ಅಥವಾ ಆರಾಮವಾಗಿರಬೇಕೆಂದು ಬಯಸುತ್ತೇನೆ
ಸಿ. ಇನ್ನೂ ಖಚಿತವಾಗಿಲ್ಲ

ಬದಲಿ ನಿರ್ಧಾರ ಮಾಡುವವರು
ನಿಮ್ಮ ಆಯ್ಕೆಗಳಿಗೆ ಧ್ವನಿ ನೀಡಲು ಅಥವಾ ನಿಮ್ಮ ವೈದ್ಯಕೀಯ ಚಿಕಿತ್ಸೆಗೆ ಸಂಬಂಧಿಸಿದ ನಿರ್ಧಾರಗಳನ್ನು ಮಾಡಲು ನಿಮಗೆ ಸಾಧ್ಯವಾಗದಿದ್ದರೆ, ನಿಮ್ಮ ಪ್ರಕಾರ ನಿಮ್ಮ ಪರವಾಗಿ ವೈದ್ಯಕೀಯ ನಿರ್ಧಾರಗಳನ್ನು ಯಾರು ತೆಗೆದುಕೊಳ್ಳಬೇಕು?
ಎ. ನನ್ನ ಕುಟುಂಬ.... (ಖಚಿತವಾಗಿ)
ಬಿ. ನನ್ನ ವೈದ್ಯರು
ಸಿ. ನನ್ನ ಕುಟುಂಬ, ನನ್ನ ವೈದ್ಯರೊಂದಿಗೆ ಚರ್ಚಿಸಿದ ನಂತರ
ಡಿ. ಯಾವುದೇ ಇತರೆ.... (ಸೂಚಿಸಿ)

ನಿಮ್ಮ ಸಮಯಕ್ಕಾಗಿ ಧನ್ಯವಾದಗಳು. ನಿಮ್ಮ ಆಯ್ಕೆಗಳನ್ನು ನಾವು ದಾಖಲಿಸಿದ್ದೇವೆ. ನಿಮ್ಮ ಕುಟುಂಬ/ ಅಥವಾ ಪ್ರೀತಿಪಾತ್ರರ ಜೊತೆಗೆ ನಿಮ್ಮ ಯೋಜನೆಗಳು ಮತ್ತು ಆಯ್ಕೆಗಳ ಬಗ್ಗೆ ಚರ್ಚಿಸಲು ನೀವು ಬಯಸಬಹುದು ಮತ್ತು ಈ ಆಯ್ಕೆಗಳನ್ನು ಎಷ್ಟು ಬಾರಿ ಬೇಕಾದರೂ ಮರುಪರಿಶೀಲಿಸಬಹುದು. ದಯವಿಟ್ಟು ನಿಮ್ಮ ವೈದ್ಯರು ಅಥವಾ ಇತರ ಡಯಾಲಿಸಿಸ್ ತಂಡದ ಸದಸ್ಯರೊಂದಿಗೆ ಇದನ್ನು ಚರ್ಚಿಸಲು ಹಿಂಜರಿಯಬೇಡಿ.

ಮೇಲಿನ ಉತ್ತರಗಳು ಖಚಿತವಾಗಿದ್ದಲ್ಲಿ ನೀವು ನಿಮ್ಮ ಆರೋಗ್ಯದ ಆದ್ಯತೆಗಳನ್ನು ದಾಖಲಾಯಿಸಲು ಬಯಸುತ್ತೀರಾ?
ಹೌದು/ ಇಲ್ಲ.

Supplementary -8

Pairwise comparison of Symptom burden, quality of life and illness-information preferences and ACP-decision-making preferences between intervention and control groups in kidney failure patients

Table S1 -Pairwise comparison of Symptom burden between intervention and control groups in ESKD patients

	Intervention (ITT- N=90, PP- N=76)										Control((ITT -N=90, PP- N=88)									
Outcome	Friedman χ^2(df)	p (overall)	Kendall's W=N×(k−1)χ2	Pairwise Comparison	Wilcoxon W	p (post-hoc; unadjusted)	Mean Difference (M1 – M2)	SE difference	95% CI (Lower, Upper)	Rank-biserial r	Friedman χ^2(df)	p (overall)	Kendall's W=N×(k−1)χ2	Pairwise Comparison	Wilcoxon W	p (post-hoc; unadjusted)	Mean Difference (M1 – M2)	SE difference	95% CI (Lower, Upper)	Effect Size Rank-biserial r
Pain	ITT- $\chi^2(3)=21.6$	<0.01	0.080								ITT- $\chi^2(3)=5.41$	0.15	0.020							
	PP- $\chi^2(3)=20.5$	<0.01	0.135								PP- $\chi^2(3)=5.28$	0.152	0.020							
				Baseline vs 2 months	1252	0.006	1.50	0.323	0.50, 2.50	0.414				Baseline vs 2 months	910	0.510	0.50	0.306	[−0.50, 1.00]	0.100
				Baseline vs 4 months	1155	< .001	2.00	0.329	1.00, 3.00	0.555				Baseline vs 4 months	933	0.928	0.00	0.315	[−1.00, 1.00]	−0.014
				Baseline vs 6 months	1195	< .001	2.00	0.327	1.00, 3.00	0.609				Baseline vs 6 months	930	0.188	−0.50	0.361	[−1.50, 0.00]	−0.184
Tiredness(Fatigue)	ITT- $\chi^2(3)=48.2$	<0.01	0.179								ITT- $\chi^2(3)=6.29$	0.099	0.023							
	PP- $\chi^2(3)=47.4$	<0.01	0.312								PP- $\chi^2(3)=6.29$	0.099	0.024							
				Baseline vs 2 months	1163	0.284	0.50	0.253	−0.50, 1.00	0.154				Baseline vs 2 months	1037	0.226	−0.50	0.261	−1.00, 0.00	−0.17
				Baseline vs 4 months	2059	0.002	1.50	0.332	0.50, 2.00	0.407				Baseline vs 4 months	1391	0.665	0.00	0.308	−0.50, 1.00	0.06
				Baseline vs 6 months	2109	< .001	2.00	0.324	1.00, 2.50	0.605				Baseline vs 6 months	1110	0.558	0.00	0.299	−1.00, 0.50	−0.08
Drowsiness	ITT- $\chi^2(3)=13.2$	<0.01	0.049								ITT- $\chi^2(3)=7.45$	0.07	0.028							
	PP- $\chi^2(3)=12.2$	<0.01	0.080								PP- $\chi^2(3)=6.62$	0.08	0.025							

	Intervention (ITT- N=90, PP- N=76)										Control((ITT -N=90, PP- N=88)									
Outcome	Friedman χ^2(df)	p (overall)	Kendall's W=N×(k−1)χ2	Pairwise Comparison	Wilcoxon W	p (post-hoc; unadjusted)	Mean Difference (M1 – M2)	SE difference	95% CI (Lower, Upper)	Rank-biserial r	Friedman χ^2(df)	p (overall)	Kendall's W=N×(k−1)χ2	Pairwise Comparison	Wilcoxon W	p (post-hoc; unadjusted)	Mean Difference (M1 – M2)	SE difference	95% CI (Lower, Upper)	Effect Size Rank-biserial r
				Baseline vs 2 months	493	0.032	1.00	0.180	0.000022, 2.00	0.401				Baseline vs 2 months	438	0.014	−1.50	0.305	−2.50, 0.00	−0.39
				Baseline vs 4 months	527	0.008	1.50	0.222	0.50, 2.00	0.498				Baseline vs 4 months	648	0.106	−1.00	0.290	−2.00, 0.00	−0.24
				Baseline vs 6 months	440	0.004	1.50	0.193	0.50, 2.00	0.569				Baseline vs 6 months	710	0.965	≈ 0.00	0.312	−1.00, 1.00	−0.01
Nausea	ITT- $\chi^2(3)=7.68$	<0.01	0.028								ITT- $\chi^2(3)=18.3$	<0.01	0.068							
	PP- $\chi^2(3)=7.07$	<0.01	0.047								PP- $\chi^2(3)=17.3$	<0.01	0.065							
				Baseline vs 2 months	423	0.451	0.50	0.204	−0.50, 1.00	0.140				Baseline vs 2 months	394	0.029	−1.00	0.240	−2.00, 0.00	−0.36
				Baseline vs 4 months	458	0.006	1.50	0.209	0.50, 2.50	0.539				Baseline vs 4 months	543	0.004	−1.50	0.268	−2.00, −0.50	−0.43
				Baseline vs 6 months	584	0.019	1.00	0.219	0.000018, 2.00	0.423				Baseline vs 6 months	453	0.003	−2.00	0.279	−2.50, −0.50	−0.45
Lack of appetite	ITT- $\chi^2(3)=18.5$	<0.01	0.069								ITT- $\chi^2(3)=4.4$	0.221	0.016							
	PP- $\chi^2(3)=20.6$	<0.01	0.136								PP-$\chi^2(3)=3.82$	0.282	0.014							
				Baseline vs 2 months	1007	0.023	1.00	0.291	0.000016, 2.00	0.356				Baseline vs 2 months	791	0.264	−0.50	0.316	−1.50, 0.50	−0.16
				Baseline vs 4 months	860	< .001	2.50	0.261	2.00, 3.50	0.737				Baseline vs 4 months	766	0.628	0.00	0.265	−1.50, 0.50	−0.07
				Baseline vs 6 months	1072	< .001	1.50	0.299	0.50, 2.50	0.555				Baseline vs 6 months	602	0.109	−0.89	0.277	−2.00, 0.00	−0.25
Shortness of breath	ITT- $\chi^2(3)=17.6$	<0.01	0.065								ITT- $\chi^2(3)=2.44$	0.486	0.009							
	PP- $\chi^2(3)=7.74$	0.05	0.051								PP- $\chi^2(3)=2.07$	0.558	0.008							
				Baseline vs 2 months	435	0.210	0.50	0.247	−0.50, 2.00	0.236				Baseline vs 2 months	652	0.434	−0.50	0.282	−1.50, 0.50	−0.12
				Baseline vs 4 months	546	0.132	0.50	0.244	−0.000012, 2.00	0.268				Baseline vs 4 months	603	0.430	−0.50	0.257	−1.50, 0.50	−0.13
				Baseline vs 6 months	502	0.002	2.00	0.246	0.50, 2.50	0.592				Baseline vs 6 months	668	0.392	−0.50	0.263	−1.50, 0.50	−0.13

	Intervention (ITT- N=90, PP- N=76)										Control((ITT -N=90, PP- N=88)									
Outcome	Friedman χ^2(df)	p (overall)	Kendall's W=N×(k−1)χ2	Pairwise Comparison	Wilcoxon W	p (post-hoc; unadjusted)	Mean Difference (M1 – M2)	SE difference	95% CI (Lower, Upper)	Rank-biserial r	Friedman χ^2(df)	p (overall)	Kendall's W=N×(k−1)χ2	Pairwise Comparison	Wilcoxon W	p (post-hoc; unadjusted)	Mean Difference (M1 – M2)	SE difference	95% CI (Lower, Upper)	Effect Size Rank-biserial r
Depression	ITT- $\chi^2(3)=8.28$	0.04	0.031								ITT- $\chi^2(3)=6.16$	0.104	0.023							
	PP- $\chi^2(3)=8.58$	0.035	0.056								PP- $\chi^2(3)=7$	0.072	0.026							
				Baseline vs 2 months	562	0.172	−0.50	0.275	−1.50, 4.92×10^{-5}	−0.215				Baseline vs 2 months	602	0.565	−0.50	0.316	−1.50, 0.50	−0.09
				Baseline vs 4 months	501	0.361	0.50	0.260	−0.50, 1.50	0.164				Baseline vs 4 months	697	0.303	−0.50	0.318	−2.00, 0.50	−0.16
				Baseline vs 6 months	673	0.146	0.50	0.250	-4.61×10^{-5}, 1.50	0.245				Baseline vs 6 months	684	0.087	−1.00	0.301	−1.50, 0.00	−0.25
	ITT- $\chi^2(3)=12.5$	0.006	0.046								ITT- $\chi^2(3)=5.41$	0.06	0.035							
Anxiety	PP- $\chi^2(3)=13.8$	0.003	0.091								$\chi^2(3)=5.4$	0.068	0.033							
				Baseline vs 2 months	504	0.526	−0.50	0.258	−1.00, 0.50	−0.106				Baseline vs 2 months	561	0.7	0.00	0.264	−1.00, 1.00	−0.05
				Baseline vs 4 months	478	0.544	≈0.00004	0.221	−0.50, 1.50	0.109				Baseline vs 4 months	532	0.06	−0.50	0.261	−1.50, 0.00	−0.28
				Baseline vs 6 months	390	0.005	2.00	0.222	0.50, 3.00	0.573				Baseline vs 6 months	452	0.060	−1.00	0.274	−2.00, 0.00	−0.34
Poor Wellbeing	ITT- $\chi^2(3)=17.9$	<0.01	0.066								ITT- $\chi^2(3)=3.57$	0.312	0.013							
	PP- $\chi^2(3)=24.1$	<0.01	0.159								PP- $\chi^2(3)=3.59$	0.309	0.013							
				Baseline vs 2 months	897	0.444	−0.50	0.271	−1.00, 0.50	−0.111				Baseline vs 2 months	972	0.389	0.00	0.211	−1.00, 0.00	−0.12
				Baseline vs 4 months	1425	0.192	0.50	0.293	−0.0000385, 1.00	0.180				Baseline vs 4 months	1250	0.969	0.00	0.252	−0.50, 1.00	0.01
				Baseline vs 6 months	1513	0.009	1.00	0.271	0.50, 2.00	0.369				Baseline vs 6 months	785	0.915	0.00	0.211	−1.00, 0.50	−0.02
Itching	ITT- $\chi^2(3)=9.76$	0.021	0.036								ITT- $\chi^2(3)=5.61$	0.06	0.036							
	PP- $\chi^2(3)=9.17$	0.03	0.060								PP- $\chi^2(3)=5.93$	0.06	0.033							
				Baseline vs 2 months	639	0.045	1.00	0.247	0.000084, 2.00	0.350				Baseline vs 2 months	571	0.676	0.00	0.258	−1.50, 0.50	−0.07

	Intervention (ITT- N=90, PP- N=76)										Control((ITT -N=90, PP- N=88)									
Outcome	Friedman χ^2(df)	p (overall)	Kendall's W=N×(k−1)χ2	Pairwise Comparison	Wilcoxon W	p (post-hoc; unadjusted)	Mean Difference (M1 − M2)	SE difference	95% CI (Lower, Upper)	Rank-biserial r	Friedman χ^2(df)	p (overall)	Kendall's W=N×(k−1)χ2	Pairwise Comparison	Wilcoxon W	p (post-hoc; unadjusted)	Mean Difference (M1 − M2)	SE difference	95% CI (Lower, Upper)	Effect Size Rank-biserial r
				Baseline vs 4 months	668	0.002	1.50	0.230	0.50, 2.50	0.551				Baseline vs 4 months	447	0.060	−1.00	0.241	−2.00, 0.00	−0.40
				Baseline vs 6 months	675	0.005	1.00	0.230	0.50, 2.00	0.495				Baseline vs 6 months	376	0.061	−1.50	0.292	−2.50, −0.50	−0.41
Sleeplessness	ITT-$\chi^2(3)=20.8$	<0.01	0.077								ITT-$\chi^2(3)=8$	0.056	0.029							
	PP-$\chi^2(3)=18.5$	<0.01	0.122								PP-$\chi^2(3)=7.4$	0.06	0.028							
				Baseline vs 2 months	1078	0.010	1.50	0.330	0.50, 2.50	0.399				Baseline vs 2 months	631	0.036	−1.00	0.311	−2.00, 0.00	−0.31
				Baseline vs 4 months	1256	0.002	1.50	0.317	0.50, 2.50	0.468				Baseline vs 4 months	710	0.129	−0.50	0.317	−2.00, 0.00	−0.23
				Baseline vs 6 months	1356	< .001	2.50	0.282	1.50, 3.00	0.760				Baseline vs 6 months	714	0.012	−1.00	0.313	−2.00, −0.50	−0.36
Others	ITT-$\chi^2(3)=18.8$	<0.01	0.070								ITT-$\chi^2(3)=5.31$	0.150	0.020							
	PP-$\chi^2(3)=14.8$	0.002	0.097								PP-$\chi^2(3)=5.31$	0.150	0.020							
				Baseline vs 2 months	830	< .001	2.00	0.334	1.00, 3.50	0.603				Baseline vs 2 months	534	0.072	−1.00	0.300	−2.00, 0.00	−0.28
				Baseline vs 4 months	796	< .001	2.50	0.334	1.50, 4.00	0.683				Baseline vs 4 months	683	0.662	0.00	0.275	−0.50, 1.00	0.07
				Baseline vs 6 months	627	< .001	3.00	0.317	2.00, 4.50	0.782				Baseline vs 6 months	557	0.861	0.00	0.300	−1.00, 1.50	0.03
Overall	ITT-$\chi^2(3)=45$	<0.01	0.167								ITT-$\chi^2(3)=7.65$	0.06	0.028							
	$\chi^2(3)=44.1$	<0.01	0.290								$\chi^2(3)=7$	0.07	0.026							
				Baseline vs 2 months	2131	0.003	4.50	1.44	1.50, 7.50	0.383				Baseline vs 2 months	1186	0.002	−4.00	1.27	−6.50, −1.50	−0.38
				Baseline vs 4 months	2686	< .001	9.00	1.49	6.00, 12.50	0.658				Baseline vs 4 months	1442	0.065	−3.00	1.44	−6.00, 0.00	−0.23

	Intervention (ITT- N=90, PP- N=76)										Control((ITT -N=90, PP- N=88)									
Outcome	Friedman χ^2(df)	p (overall)	Kendall's W=N×(k−1)χ2	Pairwise Comparison	Wilcoxon W	p (post-hoc; unadjusted)	Mean Difference (M1 − M2)	SE difference	95% CI (Lower, Upper)	Rank-biserial r	Friedman χ^2(df)	p (overall)	Kendall's W=N×(k−1)χ2	Pairwise Comparison	Wilcoxon W	p (post-hoc; unadjusted)	Mean Difference (M1 − M2)	SE difference	95% CI (Lower, Upper)	Effect Size Rank-biserial r
				Baseline vs 6 months	2523	< .001	11.50	1.55	8.50, 15.00	0.771				Baseline vs 6 months	1072	< .001	−5.50	1.42	−8.50, −2.50	−0.41

*ITT- Intention-to-Treat,*PP- Per-Protocol

Table S2 -Pairwise comparison of quality of life between intervention and control groups in ESKD patients

	Intervention (ITT- N=90, PP- N=76)										Control((ITT -N=90, PP- N=88)									
Outcome	Friedman χ^2(df)	p (overall)	Kendall's W=N×(k−1)χ 2	Pairwise Comparison	Wilcoxon W	p (post-hoc; unadjusted)	Mean Difference (M1 − M2)	SE difference	95% CI (Lower, Upper)	Rank-biserial r	Friedman χ^2(df)	p (overall)	Kendall's W=N×(k−1)χ 2	Pairwise Comparison	Wilcoxon W	p (post-hoc; unadjusted)	Mean Difference (M1 − M2)	SE difference	95% CI (Lower, Upper)	Effect Size Rank biserial r
Mobility	ITT- χ^2= 3(30.9)	<0.01	0.135								ITT- χ^2= 3(0.054)	0.99	0.0002							
	PP- χ^2= 3(33.3)	<0.01	0.219								PP-χ^2= 3(0.066)	0.99	0.0002							
				Baseline-2months	757	0.035	0.50	0.129	0.000056 , 1.00	0.341				Baseline-2months	721	0.564	1.05×10^{-5}	0.109	(-7.47×10^{-8}, 0.500)	0.0875
				Baseline-4months	824	0.005	1.00	0.145	0.000040 , 1.50	0.460				Baseline-4months	557	0.855	0.00	0.113	(−0.500, 0.500)	0.0305
				Baseline-6months	1215	< .001	1.50	0.146	1.00, 1.50	0.697				Baseline-6months	713	0.793	0.00	0.129	(−0.500, 0.500)	−0.0404
Self care	ITT- χ^2= 3(7.7)	0.05	0.034								ITT- χ^2= 3(7.73)	0.06	0.029							
	PP-χ^2= 3(12)	<0.01	0.079								PP-χ^2= 3(6.93)	0.074	0.0257							

	Intervention (ITT- N=90, PP- N=76)										Control((ITT -N=90, PP- N=88)									
Outcome	Friedman χ^2(df)	p (overall)	Kendall's W=N×(k−1)X 2	Pairwise Comparison	Wilcoxon W	p (post-hoc; unadjusted)	Mean Difference (M1 – M2)	SE difference	95% CI (Lower, Upper)	Rank-biserial r	Friedman χ^2(df)	p (overall)	Kendall's W=N×(k−1)X 2	Pairwise Comparison	Wilcoxon W	p (post-hoc; unadjusted)	Mean Difference (M1 – M2)	SE difference	95% CI (Lower, Upper)	Effect Size Rank biserial r
				Baseline-2months	612	0.799	≈0.000016	0.143	−0.50, 1.00	0.041				Baseline-2months	557	0.144	-7.47×10^{-5}	0.124	$(-1.000, 8.89 \times 10^{-6})$	−0.222
				Baseline-4months	640	0.268	≈0.000056	0.148	≈0.000013, 1.00	0.184				Baseline-4months	555	0.095	−0.500	0.128	$(-1.000, 3.11 \times 10^{-5})$	−0.253
				Baseline-6months	593	0.011	1.00	0.131	≈0.000029, 1.50	0.446				Baseline-6months	519	0.002	−0.500	0.123	$(-1.000, -1.89 \times 10^{-5})$	−0.433
Usual activities	ITT- χ^2= 3(41.5)	<0.01	0.183								ITT- χ^2= 3(4.28)	0.233	0.016							
	PP-χ^2= 3(47.9)	<0.01	0.315								PP-χ^2= 3(3.7)	0.296	0.014							
				Baseline-2months	747	0.045	0.50	0.136	≈0, 1.00	0.324				Baseline-2months	702	0.718	0.00	0.132	(−1.000, 0.500)	−0.0545
				Baseline-4months	1017	< .001	1.50	0.160	0.50, 1.50	0.595				Baseline-4months	733	0.446	-2.71×10^{-5}	0.143	$(-0.500, 1.19 \times 10^{-5})$	−0.1131
				Baseline-6months	1308	< .001	1.50	0.143	1.00, 2.00	0.827				Baseline-6months	885	0.199	-7.44×10^{-5}	0.129	$(-1.000, 3.20 \times 10^{-5})$	−0.1748
Pain / Discomfort	ITT- χ^2= 3(40.2)	<0.01	0.177								ITT- χ^2= 3(6.98)	0.08	0.0233							
	PP-χ^2= 3(44.8)	<0.01	0.295								PP-χ^2= 3(6.17)	0.104	0.0233							
				Baseline-2months	920	0.051	≈0.00004	0.108	≈0, 1.00	0.286				Baseline-2months	714	0.076	-9.34×10^{-5}	0.114	$(-1.000, 5.86 \times 10^{-5})$	−0.245
				Baseline-4months	1102	< .001	1.00	0.124	0.50, 1.00	0.539				Baseline-4months	673	0.038	-3.09×10^{-6}	0.116	$(-1.000, -6.11 \times 10^{-6})$	−0.288
				Baseline-6months	1183	< .001	1.00	0.115	1.00, 1.50	0.784				Baseline-6months	698	0.060	-1.84×10^{-6}	0.118	$(-1.000, 2.19 \times 10^{-5})$	−0.262
Anxiety / Depression	ITT- χ^2= 3(24.1)	<0.01	0.10								ITT- χ^2= 3(6.17)	0.104	0.023							
	PP-χ^2= 3(25.6)	<0.01	0.168								PP-χ^2= 3(5.58)	0.134	0.021							
				Baseline-2months	855	0.204	≈0.00001	0.146	≈0, 1.00	0.195				Baseline-2months	689	0.635	-4.98×10^{-5}	0.129	$(-1.000, 3.30 \times 10^{-5})$	−0.0721

	Intervention (ITT- N=90, PP- N=76)										Control((ITT -N=90, PP- N=88)									
Outcome	Friedman χ^2(df)	p (overall)	Kendall's W=N×(k−1)X 2	Pairwise Comparison	Wilcoxon W	p (post-hoc; unadjusted)	Mean Difference (M1 – M2)	SE difference	95% CI (Lower, Upper)	Rank-biserial r	Friedman χ^2(df)	p (overall)	Kendall's W=N×(k−1)X 2	Pairwise Comparison	Wilcoxon W	p (post-hoc; unadjusted)	Mean Difference (M1 – M2)	SE difference	95% CI (Lower, Upper)	Effect Size Rank biserial r
				Baseline-4months	1104	0.090	0.50	0.150	≈0, 1.00	0.247				Baseline-4months	681	0.580	0.000	0.117	(−0.500, 1.90 × 10^{-5})	−0.0828
				Baseline-6months	1215	< .001	1.50	0.128	1.00, 1.50	0.763				Baseline-6months	540	0.072	−0.500	0.129	(−1.0, 5.46 × 10^{-5})	−0.2734
Overall Qol	ITT- χ^2= 3(46.5)	<0.01	0.20								ITT- χ^2= 3(7.70)	0.06	0.029							
	PP-χ^2= 3(54.6)	<0.01	0.359								PP-χ^2= 3(8.52)	0.06	0.036							
				Baseline-2months	1735	0.034	1.00	0.446	≈0, 2.00	0.284				Baseline-2months	1106	0.128	−1.000	0.419	(−1.50, 8.03 × 10^{-6})	−0.203
				Baseline-4months	1881	< .001	2.00	0.548	1.00, 3.50	0.472				Baseline-4months	1124	0.110	−0.500	0.407	(−2.00, 3.92 × 10^{-5})	−0.212
				Baseline-6months	2238	< .001	4.00	0.485	3.00, 5.00	0.853				Baseline-6months	1104	0.029	−1.000	0.454	(−2.50, −5.10 × 10^{-5})	−0.284
Health	ITT- χ^2= 3(46.9)	<0.01	0.20								ITT- χ^2= 3(7.5)	0.06	0.028							
	PP-χ^2= 3(51.2)	<0.01	0.337								PP-χ^2= 3(7.6)	0.06	0.029							
				Baseline-2months	704	0.440	−2.50	1.50	−7.50, 2.50	−0.118				Baseline-2months	1865	0.03	7.50	1.79	(2.50, 12.50)	0.381
				Baseline-4months	624	0.013	−7.50	1.68	−10.00, ≈0.00	−0.361				Baseline-4months	1712	0.128	5.00	1.89	(~0, 7.50)	0.201
				Baseline-6months	389	< .001	−12.50	1.96	−17.50, −7.50	−0.669				Baseline-6months	1591	0.021	7.50	1.76	(~0, 10.00)	0.317

*ITT- Intention-to-Treat,*PP- Per-Protocol

Table S3 -Pairwise comparison of illness-information preferences between intervention and control groups in ESKD patients

Intervention (ITT- N=90, PP- N=76)											Control((ITT -N=90, PP- N=88)									
**Outcome**	**Friedman χ^2(df)**	**p (overall)**	**Kendall's W=N×(k−1)χ2**	**Pairwise Comparison**	**Wilcoxon W**	**p (post-hoc; unadjusted)**	**Mean Difference (M1 – M2)**	**SE difference**	**95% CI (Lower, Upper)**	**Rank-biserial r**	**Friedman χ^2(df)**	**p (overall)**	**Kendall's W=N×(k−1)χ2**	**Pairwise Comparison**	**Wilcoxon W**	**p (post-hoc; unadjusted)**	**Mean Difference (M1 – M2)**	**SE difference**	**95% CI (Lower, Upper)**	**Effect Size Rank-biserial r**
A1.1	ITT- $\chi^2(2)=6.2$	0.04	0.032								ITT- $\chi^2(2)=0.70$	0.702	0.003							
	PP- $\chi^2(2)=6.16$	0.04	0.041								PP- $\chi^2(2)=0.70$	0.702	0.004							
				2month-4months	387	0.04	−0.50	0.167	−1.50, 3.66×10^{-5}	−0.285				2month-4months	641	0.159	0.500	0.170	(-1.91×10^{-5}, 1.50)	0.238
				2month-6months	222	0.006	−1.000	0.153	−1.50, -7.38×10^{-5}	−0.484				2month-6months	342	0.448	3.23×10^{-5}	0.134	(−0.50, 1.00)	0.148
A1.2	ITT- $\chi^2(2)=13.4$	<0.01	0.074								ITT- $\chi^2(2)=1.99$	0.37	0.011							
	PP- $\chi^2(2)=15.3$	<0.01	0.101								PP- $\chi^2(2)=1.99$	0.37	0.0113							
				2month-4months	612	0.476	-6.25×10^{-5}	0.160	−1.0, 0.5	−0.112				2month-4months	810	0.003	1.00	0.173	(0.50, 1.50)	0.499
				2month-6months	257	< .001	−1.000	0.163	−1.500, −0.500	−0.642				2month-6months	322	0.461	~0 (2.15×10^{-5})	0.141	(−0.50, 1.00)	0.146
A1.3	ITT- $\chi^2(2)=6.07$	0.04	0.034								ITT- $\chi^2(2)=1.37$	0.504	0.0076							
	**PP-$\chi^2(2)$= 6.07**	0.04	0.041								**PP-$\chi^2(2)$= 1.37**	0.504	0.0077							
				2month-4months	702	0.04	4.84×10^{-5}	0.170	−0.500, 0.500	0.101				2month-4months	779	0.166	0.500	0.198	(-7.10×10^{-6}, 1.50)	0.222
				2month-6months	478	0.04	−0.500	0.165	−1.000, 1.16×10^{-5}	−0.306				2month-6months	328	0.602	~0 (6.36×10^{-5})	0.150	(−0.50, 1.00)	0.103
A1.4	ITT- $\chi^2(2)=15.7$	<0.01	0.087								ITT- $\chi^2(2)=2.39$	0.303	0.013							
	**PP- $\chi^2(2)=16.5$**	<0.01	0.109								**PP- $\chi^2(2)=2.39$**	0.303	0.013							
				2month-4months	576	0.297	−0.500	0.187	−1.000, 0.500	−0.164				2month-4months	625	0.063	0.771	0.178	(-7.04×10^{-5}, 1.50)	0.321
				2month-6months	308	< .001	−1.500	0.183	−2.0, −1.0	−0.640				2month-6months	215	0.534	~0 (7.05×10^{-6})	0.136	(−1.00, 1.50)	0.138
A1.5	ITT- $\chi^2(2)=18.5$	<0.01	0.10								ITT- $\chi^2(2)=4.56$	0.10	0.024							
	**χ^2 2=19.5**	<0.01	0.128								**PP-$\chi^2$2=4.56**	0.104	0.025							

Intervention (ITT- N=90, PP- N=76)											Control((ITT -N=90, PP- N=88)									
Outcome	Friedman χ^2(df)	p (overall)	Kendall's W=N×(k−1)χ2	Pairwise Comparison	Wilcoxon W	p (post-hoc; unadjusted)	Mean Difference (M1 – M2)	SE difference	95% CI (Lower, Upper)	Rank-biserial r	Friedman χ^2(df)	p (overall)	Kendall's W=N×(k−1)χ2	Pairwise Comparison	Wilcoxon W	p (post-hoc; unadjusted)	Mean Difference (M1 – M2)	SE difference	95% CI (Lower, Upper)	Effect Size Rank-biserial r
				2month-4months	566	0.123	−0.500	0.176	−1.50, 5.99×10^{-5}	−0.238				2month-4months	663	0.020	1.000	0.168	(4.46×10^{-5}, 1.50)	0.402
				2month-6months	245	< .001	−1.500	0.206	−2.00, −1.000	−0.670				2month-6months	331	0.099	0.500	0.131	(-4.14×10^{-5}, 1.50)	0.333
A1.6	ITT-$\chi^2(2)$=21.4	<0.01	0.12								ITT-$\chi^2(2)$=4.32	0.115	0.024							
	PP-χ^2 2=23.5	<0.01	0.155								PP-$\chi^2$2=4.32	0.115	0.025							
				2month-4months	486	0.198	−0.500	0.157	−1.00, ~0.00	−0.207				2month-4months	806	0.101	0.500	0.201	(-5.50×10^{-5}, 1.500)	0.2635
				2month-6months	265	< .001	−1.500	0.193	−2.00, −1.000	−0.701				2month-6months	298	0.783	0.000	0.148	(−1.000, 0.500)	0.540
A1.7	ITT-$\chi^2(2)$=20.5	<0.01	0.113								ITT-$\chi^2(2)$=5.91	0.06	0.034							
	PP-χ^2 2=22.2	<0.01	0.146								PP-$\chi^2$2=5.91	0.06	0.033							
				2month-4months	511	0.940	0.00	0.163	−0.500, 0.500	−0.0135				2month-4months	729	0.037	1.000	0.200	(3.82×10^{-5}, 1.500)	0.3488
				2month-6months	213	< .001	−1.50	0.191	−2.000, −1.000	−0.7138				2month-6months	272	0.113	~0.500	0.135	(−1.00, 1.500)	0.340
A1.8	ITT-$\chi^2(2)$=17	<0.01	0.09								ITT-$\chi^2(2)$=4.86	0.08	0.027							
	PP-χ^2=18.7	<0.01	0123								PP-$\chi^2$2=4.86	0.08	0.027							
				2month-4months	504	0.684	0.00	0.160	−0.500, 0.500	−0.0685				2month-4months	463	0.176	0.500	0.154	(-2.48×10^{-5}, 1.500)	0.248
				2month-6months	244	<.001	−1.0	0.153	−1.500, −1.000	−0.6590				2month-6months	142	0.392	- 0.500	0.118	(1.00, 0.500)	0.191

*A1.1- A1.8 Illness information of ACP questionaries(ANNEXURE-11) *ITT- Intention-to-Treat,*PP- Per-Protocol

Table S4 -Pairwise comparison of ACP- decision making preferences between intervention and control groups in ESKD patients

Intervention (ITT- N=90, PP- N=76)											Control((ITT -N=90, PP- N=88)										
Outcome	Friedman χ^2(df)	p (overall)	Kendall's W=N×(k−1)χ2	Pairwise Comparison	Wilcoxon W	p (post-hoc; unadjusted)	Mean Difference (M1 – M2)	SE difference	95% CI (Lower, Upper)	Effect Size Rank-biserial r	Friedman χ^2(df)	p (overall)	Kendall's W=N×(k−1)χ2	Pairwise Comparison	Wilcoxon W	p (post-hoc; unadjusted)	Mean Difference (M1 – M2)	SE difference	95% CI (Lower, Upper)	Effect Size Rank-biserial r
A2.1	ITT- $\chi^2(2)=31.5$	<0.01	0.176								ITT- $\chi^2(2)=1.54$	0.46	0.0086							
	PP- $\chi^2(2)=33.7$	<0.01	0.222								PP- $\chi^2(2)=1.54$	0.463	0.00875							
				2month-4months	531	0.672	7.59×10^{-6}	0.126	−0.500, 1.000	0.0717				2month-4months	323	0.648	3.06×10^{-5}	0.0847	(-2.79×10^{-5}, 0.500)	0.0857
				2month-6months	250	< .001	−1.000	0.125	−1.000, −1.000	−0.6372				2month-6months	422	0.062	0.500	0.0874	(-3.62×10^{-6}, 1.00)	0.3397
A2.2	ITT- $\chi^2(2)=35$	<0.01	0.195								ITT- $\chi^2(2)=1.36$	0.50	0.007							
	PP- $\chi^2(2)=36.1$	<0.01	0.238								PP- $\chi^2(2)=1.36$	0.56	0.0077							
				2month-4months	511	0.446	5.55×10^{-6}	0.118	-6.82×10^{-5}, 0.500	0.131				2month-4months	292	0.489	−2.53e−5	0.0814	(−0.500, 4.79e−5)	−0.1231
				2month-6months	180	< .001	−1.000	0.117	−1.000, −1.000	−0.695				2month-6months	364	0.846	2.44e−5	0.0907	(−0.500, 0.500)	0.0356
A2.3	ITT- $\chi^2(2)=18.4$	<0.01	0.102								ITT- $\chi^2(2)=2.61$	0.27	0.014							
	PP- $\chi^2(2)=19$	<0.01	0.125								PP- $\chi^2(2)= 2.61$	0.271	0.014							
				2month-4months	608	0.835	3.65×10^{-5}	0.125	−0.500, 0.500	0.0340				2month-4months	406	0.582	0.00	0.0807	(−4.67e−5, 0.500)	0.0945
A2.4	ITT- $\chi^2(2)=3.96$	0.138	0.022								ITT- $\chi^2(2)=4.08$	0.13	0.023							

Intervention (ITT- N=90, PP- N=76)											Control((ITT -N=90, PP- N=88)									
Outcome	Friedman χ^2(df)	p (overall)	Kendall's W=N×(k−1)χ2	Pairwise Comparison	Wilcoxon W	p (post-hoc; unadjusted)	Mean Difference (M1 – M2)	SE difference	95% CI (Lower, Upper)	Effect Size Rank-biserial r	Friedman χ^2(df)	p (overall)	Kendall's W=N×(k−1)χ2	Pairwise Comparison	Wilcoxon W	p (post-hoc; unadjusted)	Mean Difference (M1 – M2)	SE difference	95% CI (Lower, Upper)	Effect Size Rank-biserial r
	PP-$\chi^2(2)=3.96$	0.138	0.026								PP-$\chi^2(2)=4.08$	0.13	0.023							
				2month-4months	138	0.70	5.17×10^{-5}	0.073	-0.5 -1.0	0.09				2month-4months	214	0.682	−2.98e−6	0.0808	(−0.500, 3.64e−5)	−0.0817
				2month-6months	296	0.17	1.000	0.09	-2.07- 1.0	0.271				2month-6months	177	0.137	−0.500	0.0868	(−1.000, 4.91e−5)	−0.2883
A2.5	ITT-$\chi^2(2)=0.47$	0.787	0.002								ITT-$\chi^2(2)=5.84$	0.06	0.03							
	PP-χ^2= 2(0.754)	0.686	0.005								PP-$\chi^2$2=5.84	0.06	0.032							
				2month-4months	232	0.493	2.60×10^{-5}	0.0878	-8.59×10^{-6}, 1.000	0.1429				2month-4months	353	0.786	0.000	0.0885	(−0.500, 6.88×10^{-5})	−0.0472
				2month-6months	236	0.678	1.58×10^{-5}	0.0955	−0.500, 0.500	0.0851				2month-6months	242	0.078	−1.000	0.0959	(−1.000, 1.82×10^{-5})	−0.3115
A2.6	ITT-$\chi^2(2)=0.78$	0.68	0.004								ITT-$\chi^2(2)=5.08$	0.08	0.02							
	PP-$\chi^2(2)=0.778$	0.68	0.005								PP-χ^2= 2(5.08)	0.08	0.02							
				2month-4months	261	0.72	-2.9-5.00	0.09	-1.0-0.5	-0.06				2month-4months	372	0.749	6.33e−5	0.0862	(−3.08e−5, 0.500)	0.0569
				2month-6months	260	0.94	-3.13- 5.0	0.09	-1.0-0.5	-0.016				2month-6months	320	0.119	−8.04e−5	0.0917	(−1.000, 2.89e−6)	0.2567
	ITT-$\chi^2(2)=8.22$	0.010	0.051								ITT-$\chi^2(2)=3.92$	0.14	0.021							
A2.7	PP-$\chi^2(2)=8.21$	0.010	0.046								PP-$\chi^2$2=3.9	0.141	0.022							
				2month-4months	311	0.004	-0.00	0.137	-1.5-2.14	-0.472				2month-4months	418	0.678	4.67×10^{-6}	0.0862	(-1.77×10^{-5}, 0.500)	0.0718

Intervention (ITT- N=90, PP- N=76)											Control((ITT -N=90, PP- N=88)									
**Outcome**	**Friedman χ^2(df)**	**p (overall)**	**Kendall's W=N×(k−1)χ2**	**Pairwise Comparison**	**Wilcoxon W**	**p (post-hoc; unadjusted)**	**Mean Difference (M1 – M2)**	**SE difference**	**95% CI (Lower, Upper)**	**Effect Size Rank-biserial r**	**Friedman χ^2(df)**	**p (overall)**	**Kendall's W=N×(k−1)χ2**	**Pairwise Comparison**	**Wilcoxon W**	**p (post-hoc; unadjusted)**	**Mean Difference (M1 – M2)**	**SE difference**	**95% CI (Lower, Upper)**	**Effect Size Rank-biserial r**
				2month-6months	198	0.01	-1.00	0.115	−1.50, 0.50	-0.58				2month-6months	348	0.249	-4.29×10^{-5}	0.0906	$(-1.000, 5.10\times10^{-5})$	−0.1916
A2.8	ITT- $\chi^2(2)=27.9$	<0.01	0.155								ITT- $\chi^2(2)=3.31$	0.19	0.018							
	PP-χ^2= 2(28.9)	<0.01	0.190								PP-χ^2= 2(3.31)	0.19	0.018							
				2month-4months	344	0.635	1.38×10^{-5}	0.113	−0.500, 1.000	0.0905				2month-4months	417	0.459	4.96×10^{-5}	0.0781	$(-9.45\times10^{-6}, 0.500)$	0.126
				2month-6months	192	< .001	−1.00	0.133	−1.500, −0.500	−0.5941				2month-6months	435	0.443	-9.25×10^{-5}	0.0852	$(-1.000, 3.79\times10^{-5})$	−0.121

*A2.1- A2.8 Decision making preferences of ACP questionaries(ANNEXURE-11) *ITT- Intention-to-Treat,*PP- Per-Protocol

Supplementary -9

CONSORT 2025 checklist of information to include when reporting a randomised trial*

Section / Topic	No	CONSORT 2025 checklist item description	Reported on page no.
**Title and abstract**			
Title and structured abstract	1a	Identification as a randomised trial	1
	1b	Structured summary of the trial design, methods, results, and conclusions	4
**Open science**			
Trial registration	2	Name of trial registry, identifying number (with URL) and date of registration<br><br>Clinical Trials Registry of India (CTRI/2021/06/034179)<br>Date of registration-11/06/2021	6
Protocol and statistical analysis plan	3	Where the trial protocol and statistical analysis plan can be accessed	6(CTRI) Supplementary-1
Data sharing	4	Where and how the individual de-identified participant data (including data dictionary), statistical code and any other materials can be accessed	14
Funding and conflicts of interest	5a	Sources of funding and other support (e.g., supply of drugs), and role of funders in the design, conduct, analysis and reporting of the trial	14
	5b	Financial and other conflicts of interest of the manuscript authors	14
**Introduction**			
Background and rationale	6	Scientific background and rationale	5,6
Objectives	7	Specific objectives related to benefits and harms	5,6
**Methods**			
Patient and public involvement	8	Details of patient or public involvement in the design, conduct and reporting of the trial	6,7
Trial design	9	Description of trial design including type of trial (e.g., parallel group, crossover), allocation ratio, and framework (e.g., superiority, equivalence, non-inferiority, exploratory)	8
Changes to trial protocol	10	Important changes to the trial after it commenced including any outcomes or analyses that were not prespecified, with reason	-

Trial setting	11	Settings (e.g., community, hospital) and locations (e.g., countries, sites) where the trial was conducted	6
Eligibility criteria	12a	Eligibility criteria for participants	6,7
	12b	If applicable, eligibility criteria for sites and for individuals delivering the interventions (e.g., surgeons, physiotherapists)	6
Intervention and comparator	13	Intervention and comparator with sufficient details to allow replication. If relevant, where additional materials describing the intervention and comparator (e.g., intervention manual) can be accessed	7,8 (supplementary-1)
Outcomes	14	Pre-specified primary and secondary outcomes, including the specific measurement variable (e.g., systolic blood pressure), analysis metric (e.g., change from baseline, final value, time to event), method of aggregation (e.g., median, proportion), and time point for each outcome	8
Harms	15	How harms were defined and assessed (e.g., systematically, non-systematically)	-
Sample size	16a	How sample size was determined, including all assumptions supporting the sample size calculation	8,9
	16b	Explanation of any interim analyses and stopping guidelines	-
Randomisation:			
Sequence generation	17a	Who generated the random allocation sequence and the method used	9
	17b	Type of randomisation and details of any restriction (e.g., stratification, blocking and block size)	9
Allocation concealment mechanism	18	Mechanism used to implement the random allocation sequence (e.g., central computer/telephone; sequentially numbered, opaque, sealed containers), describing any steps to conceal the sequence until interventions were assigned	9
Implementation	19	Whether the personnel who enrolled and those who assigned participants to the interventions had access to the random allocation sequence	9
Blinding	20a	Who was blinded after assignment to interventions (e.g., participants, care providers, outcome assessors, data analysts)	9
	20b	If blinded, how blinding was achieved and description of the similarity of interventions	9
Statistical methods	21a	Statistical methods used to compare groups for primary and secondary outcomes, including harms	9
	21b	Definition of who is included in each analysis (e.g., all randomised participants), and in which group	9
	21c	How missing data were handled in the analysis	9
	21d	Methods for any additional analyses (e.g., subgroup and sensitivity analyses), distinguishing prespecified from post-hoc	9
**Results**			

Participant flow, including flow diagram	22a	For each group, the numbers of participants who were randomly assigned, received intended intervention, and were analysed for the primary outcome	9,10
	22b	For each group, losses and exclusions after randomisation, together with reasons	9,10
Recruitment	23a	Dates defining the periods of recruitment and follow-up for outcomes of benefits and harms	9
	23b	If relevant, why the trial ended or was stopped	-
Intervention and comparator delivery	24a	Intervention and comparator as they were actually administered (e.g., where appropriate, who delivered the intervention/comparator, how participants adhered, whether they were delivered as intended [fidelity])	10
	24b	Concomitant care received during the trial for each group	10
Baseline data	25	A table showing baseline demographic and clinical characteristics for each group	10
Numbers analysed, outcomes and estimation	26	For each primary and secondary outcome, by group:<br>● the number of participants included in the analysis<br>● the number of participants with available data at the outcome time point<br>● result for each group, and the estimated effect size and its precision (such as 95% confidence interval)<br>● for binary outcomes, presentation of both absolute and relative effect size	10,11
Harms	27	All harms or unintended events in each group	-
Ancillary analyses	28	Any other analyses performed, including subgroup and sensitivity analyses, distinguishing pre-specified from post-hoc	10(Supplementary -5)
**Discussion**			
Interpretation	29	Interpretation consistent with results, balancing benefits and harms, and considering other relevant evidence	11,12
Limitations	30	Trial limitations, addressing sources of potential bias, imprecision, generalisability, and, if relevant, multiplicity of analyses	13

*We strongly recommend reading this statement in conjunction with the CONSORT 2025 Explanation and Elaboration and/or the CONSORT 2025 Expanded Checklist for important clarifications on all the items. We also recommend reading relevant CONSORT extensions. See www.consort-spirit.org.

Citation: Hopewell S, Chan AW, Collins GS, Hróbjartsson A, Moher D, Schulz KF, et al. CONSORT 2025 Statement: updated guideline for reporting randomised trials. BMJ. 2025; 388:e081123. https://dx.doi.org/10.1136/bmj-2024-081123.

© 2025 Hopewell et al. This is an Open Access article distributed under the terms of the Creative Commons Attribution License

(https://creativecommons.org/licenses/by/4.0/), which permits unrestricted use, distribution, and reproduction in any medium, provided the original work is properly cited.
